# Synthesis of highly functionalized 2,2'-bipyridines by cyclocondensation of β-ketoenamides – scope and limitations

**DOI:** 10.3762/bjoc.12.112

**Published:** 2016-06-09

**Authors:** Paul Hommes, Hans-Ulrich Reissig

**Affiliations:** 1 Freie Universität Berlin, Institut für Chemie und Biochemie, Takustrasse 3, D-14195 Berlin, Germany

**Keywords:** 2,2-bipyridines, cross couplings, cyclocondensation, β-ketoenamides, nonaflates

## Abstract

The scope of a flexible route to unsymmetrically functionalized bipyridines is described. Starting from 1,3-diketones **1a**–**e**, the corresponding β-ketoenamines **2a**–**e** were converted into different β-ketoenamides **3a**–**g** by *N*-acylation with 2-pyridinecarboxylic acid derivatives. These β-ketoenamides were treated with a mixture of TMSOTf and Hünig’s base to promote the cyclocondensation to 4-hydroxypyridine derivatives. Their immediate *O*-nonaflation employing nonafluorobutanesulfonyl fluoride provided the expected 4-nonafloxy-substituted bipyridine derivatives **5a**–**g** in moderate to good overall yields. The bipyridyl nonaflates are excellent precursors for palladium-catalyzed reactions as demonstrated by representative Suzuki and Sonogashira couplings. Thus, a library of specifically substituted bipyridine derivatives was generated, showing the versatility of the simple 1,3-diketone-based approach to this important class of ligands.

## Introduction

**Scheme 1:**
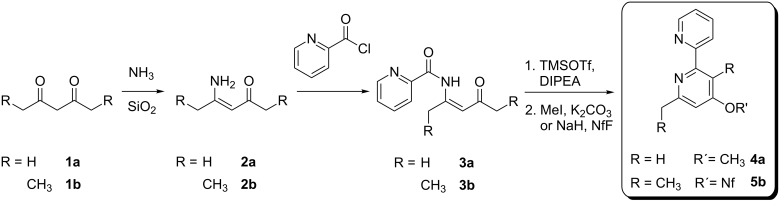
Synthesis of highly functionalized 2,2'-bipyridines **4a** and **5b** from symmetrical 1,3-diketones **1a** and **1b**; TMSOTf = trimethylsilyl trifluoromethanesulfonate; DIPEA = *N*,*N*-diisopropylethylamine; NfF = nonafluorobutanesulfonyl fluoride.

In 2009 we reported on a new pyridine synthesis starting from symmetrically substituted 1,3-diketones **1a** and **1b**, respectively, that were converted into the corresponding β-ketoenamines and subsequently by *N*-acylation into β-ketoenamides such as **3a** or **3b** [1]. Their cyclocondensation followed by *O*-alkylation or *O*-nonaflation led to functionalized pyridine derivatives. Scheme 1 illustrates this sequence with the synthesis of two 2,2´-bipyridine derivatives **4a** or **5b** that were obtained by employing picolinic acid chloride for the *N*-acylations followed by *O*-methylation with methyl iodide or by *O*-nonaflation using nonafluorobutanesulfonyl fluoride (NfF) as sulfonylating reagent. In subsequent publications we could demonstrate that this method can be used to synthesize a variety of functionalized pyridine derivatives, 2,2´-bipyridines or terpyridines [2–4]. An alternative entry to the crucial β-ketoenamides via a Blaise reaction was also developed [5], however, only a limited number of examples for the preparation of bipyridine derivatives have been reported so far. Hence we describe here additional experiments leading to this important class of compounds, in order to define scope and limitations of this reaction sequence. For this purpose we prepared other β-ketoenamides, studied their cyclocondensation reactions and used the present functionalities for subsequent reactions, in particular palladium-catalyzed coupling reactions.

2,2´-Bipyridines are a very important class of ligands employed in catalysis, supramolecular chemistry or materials science and hence a large number of specifically substituted derivatives is known (for reviews and selected recent syntheses or applications see [6–20]). Due to the flexible and modular nature of our approach to this compound class the design of new derivatives is possible thus supplementing the existing collection of available 2,2´-bipyridines.

## Results and Discussion

**Scheme 2:**
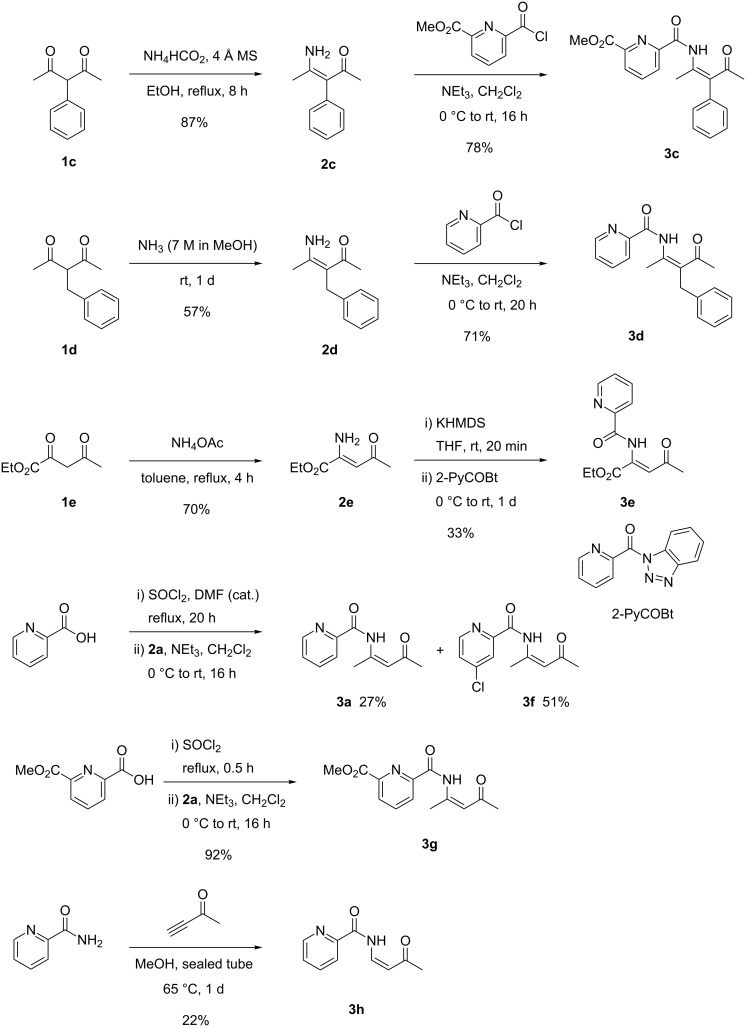
Synthesis of β-ketoenamines **2c**–**e** and of β-ketoenamides **3c–h**.

First we prepared the corresponding β-ketoenamines **2** required for the *N*-acylation step (Scheme 2). The known compounds **2a** and **2b** are available in excellent yields from the 1,3-diketones **1a** and **1b** employing aqueous ammonia in the presence of silica gel [21]. Compound **2c** was prepared from **1c** in 87% yield with ammonium formate [22–23]. Interestingly, treatment of **1c** with aqueous ammonia/silica gel failed to give **2c**, deacetylation to 1-phenylpropan-2-one (76% yield) was observed instead. For the amination of benzyl-substituted diketone **1d** both of the above utilized methods failed to provide the desired compound **2d**. While treatment with ammonium formate exclusively leads to deacetylation of the starting material to 4-phenylbutan-2-one, the reaction with aqueous ammonia/silica gel provided a mixture of the desired compound **2d** and the deacetylation product. In both methods slightly acidic reagents are employed, in contrast strictly basic conditions provided by the reaction of **1d** with a 7 M solution of ammonia in methanol allowed preparing the desired β-ketoenamine **2d** in a reasonable yield. The reaction of compound **1e** with NH_3_/SiO_2_ also mainly induced decomposition by deacetylation but the desired β-ketoenamine **2e** could be prepared in 70% yield using ammonium acetate in toluene [24]. The reaction of **1e** with ammonium formate merely provided **2e** in 42% yield.

The corresponding β-ketoenamides containing pyridine moieties at the acylated nitrogen were prepared by standard methods with the respective acid chlorides that furnished compounds **3c** and **3d** in good yields. For the synthesis of compound **3e** it was advantageous to employ the benzotriazole derivative 2-PyCOBt [25–26]. During an attempt to prepare β-ketoenamide **3a** by in situ generation of picolinic acid chloride with thionyl chloride under heating and by amide formation of the resulting product with **2a** we isolated not only the expected compound **3a** but as major component the *meta*-chlorinated compound **3f**. This subsequent chlorination of picolinic acid chloride is known from the literature to proceed under enforced conditions [27–28]. On the other hand, a selective synthesis of **3a** is possible in 73% yield by coupling of β-enaminoketone **2a** with picolinic acid under assistance of BOP as reagent (see Supporting Information File 1). The in situ generation of the acid chloride with thionyl chloride was possible without the undesired chlorination with the more electron-deficient 6-methoxycarbonyl-substituted 2-pyridine carboxylic acid delivering with **2a** the desired β-ketoenamide **3g** with excellent efficacy.

We also examined an alternative approach to β-ketoenamides by the addition of carboxylic acid amides to alkynones. This approach can potentially lead to products that are formally derived from unsymmetrically substituted 1,3-dicarbonyl compounds. The best result was obtained with picolinic acid amide and but-3-yn-2-one (as an equivalent of 3-oxobutanal) using methanol as solvent in a sealed tube [29]. Disappointingly, the maximum yield for compound **3h** was only 22%. Many attempts to improve this result – including gold catalysis [30] – either led to lower yields or no conversion at all [31]. We have to conclude that this approach to β-ketoenamides is not efficient.

With *N*-pyridyl-substituted β-ketoenamides **3a**–**h** in hand, we investigated their cyclocondensations to the corresponding 2,2´-bipyridine derivatives **5a**–**h** (Table 1). As in our preliminary experiments the use of trimethylsilyl trifluoromethanesulfonate and Hünig’s base (DIPEA) gave the best results if the mixture was heated for 3 d in 1,2-dichloroethane at 90 °C under fairly high dilution (ca. 0.04 M). Higher concentrations as reported earlier [1] gave inferior results, probably due to increased amounts of intermolecular reactions leading to oligomeric side products. The resulting primary condensation products contain a 4-hydroxy group in the newly formed pyridine ring that also exists as 4-pyridone tautomer making the identification and purification at this stage inconvenient. We therefore removed all volatile components from the crude products of the cyclocondensation step and subjected the unpurified compounds to the *O*-nonaflation procedure. Treatment with an excess of sodium hydride in THF followed by reaction with nonafluorobutanesulfonyl fluoride (NfF) afforded the desired 2,2´-bipyridine derivatives **5a**–**g** containing the 4-nonafloxy substituent in moderate to good overall yields. This functional group fixes the tautomeric structure and makes the products less polar which allowed smooth isolation, purification and identification. An additional advantage of this substituent is its ability to allow transition-metal-catalyzed coupling reactions [32]. Entries 1–7 of Table 1 demonstrate that quite differently substituted 2,2´-bipyridine derivatives are accessible by this method. Only precursor **3h** that should lead to compound **5h** with all substituents R^1^–R^5^ being hydrogen could not be isolated. We assume that the missing bulk of substituents led to undesired side reactions, e.g., deacylation or oligomerization processes.

**Table 1:** Attempted TMSOTf/DIPEA-promoted cyclocondensations of β-ketoenamides **3a**–**h** and subsequent *O*-nonaflation leading to 2,2'-bipyridines **5a**–**h**.

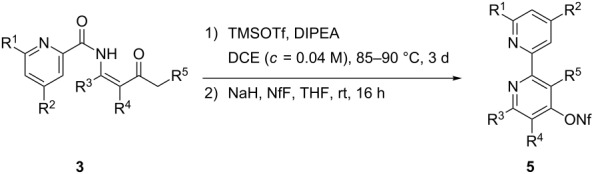							

Entry	β-Keto- enamide	R^1^	R^2^	R^3^	R^4^	R^5^	2,2'-Bipyrid-4-yl nonaflate (yield)^a^

1	**3a**	H	H	Me	H	H	**5a** (75%)
2	**3b**	H	H	Et	H	Me	**5b** (51%)^b^
3	**3c**	CO_2_Me	H	Me	Ph	H	**5c** (86%)
4	**3d**	H	H	Me	Bn	H	**5d** (54%)
5^c^	**3e**	H	H	CO_2_Et	H	H	**5e** (52%)
6	**3f**	H	Cl	Me	H	H	**5f** (66%)
7	**3g**	CO_2_Me	H	Me	H	H	**5g** (51%)
8	**3h**	H	H	H	H	H	**5h** –^d^

^a^Yield over two steps. ^b^Example taken from ref. [1]. ^c^Nf_2_O was used instead of NfF. ^d^No defined products isolated.

**Scheme 3:**
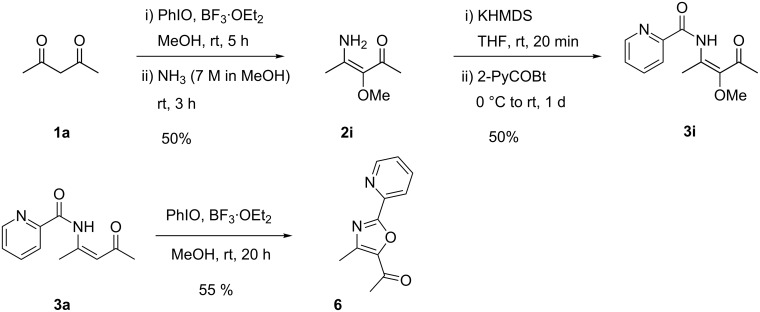
Synthesis of α-methoxy-β-ketoenamine **2i**, its *N*-acylation to **3i** and the reaction of β-ketoenamide **3a** with iodosylbenzene to 5-acetyloxazole **6**.

**Scheme 4:**
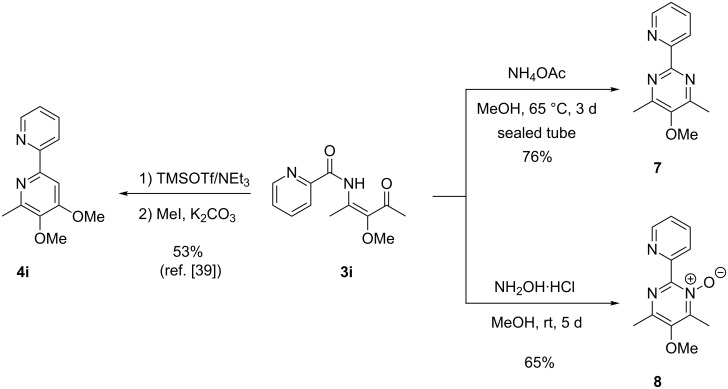
Cyclizations of β-ketoenamide **3i** leading to 2,2´-bipyridine derivative **4i** or to the related 2-(2-pyridyl)pyrimidine **7** or the corresponding *N*-oxide **8**.

The development of the discussed pyridine syntheses by cyclocondensation reactions of β-ketoenamides was inspired by a serendipitously discovered three-component reaction of lithiated alkoxyallenes, nitriles and carboxylic acids [33–40]. These components form α-alkoxy-substituted β-ketoenamides as precursors of highly substituted pyridine derivatives. We therefore tried to independently prepare β-ketoenamides with this substitution pattern starting from simple 1,3-diketones such as **1a**. An oxidative coupling of acetylacetone **1a** employing iodosylbenzene in the presence of boron trifluoride in methanol [41] followed by the standard amination procedure indeed furnished β-enaminoketone **2i** in moderate yield (Scheme 3). This intermediate could be *N*-acylated via its potassium salt with activated picolinic acid to furnish the desired α-methoxy-β-ketoenamide **3i** in 50% yield. This sequence demonstrates that this type of β-ketoenamide precursor is also available by this oxidative method; in this case the direct preparation of **3i** by the multicomponent route employing methoxyallene was similarly efficient (22% yield, [39]). It has been earlier demonstrated that **3i** can be cyclized and *O*-methylated to the corresponding bipyridine derivative **4i** in 53% yield (see Scheme 4) [39].

When we tried to employ the oxidative methoxylation to β-ketoenamide **3a** the expected methoxy-substituted product **3i** was not formed. Instead, we isolated the trisubstituted oxazole derivative **6** in 55% yield. The scope of this reaction has not been studied by us, however it is known in the literature that enamides can undergo related oxidative cyclizations to oxazoles in the presence of the reagent cocktail employed or under related reaction conditions [41–45]. In this context we want to notice that our approach to alkoxy-substituted β-ketoenamides via alkoxyallenes also allowed the synthesis of 5-acetyloxazoles with high flexibility, if the acid-labile trimethylsilylethyl group was introduced via the alkoxyallene [46–47].

As mentioned above β-ketoenamide **3i** is a good precursor for 2,2´-bipyridine derivative **4i**, but Scheme 4 also reveals that this intermediate could be converted into the expected pyrimidine derivative **7** by treatment with ammonium acetate [48–50] or into pyrimidine *N*-oxide **8** by employing hydroxylamine hydrochloride [51–52]. These products of simple condensation reactions may be regarded as azabipyridine derivatives and their synthesis demonstrates the versatility of β-ketoenamide such as **3i** for the synthesis of highly functionalized heterocycles.

**Scheme 5:**
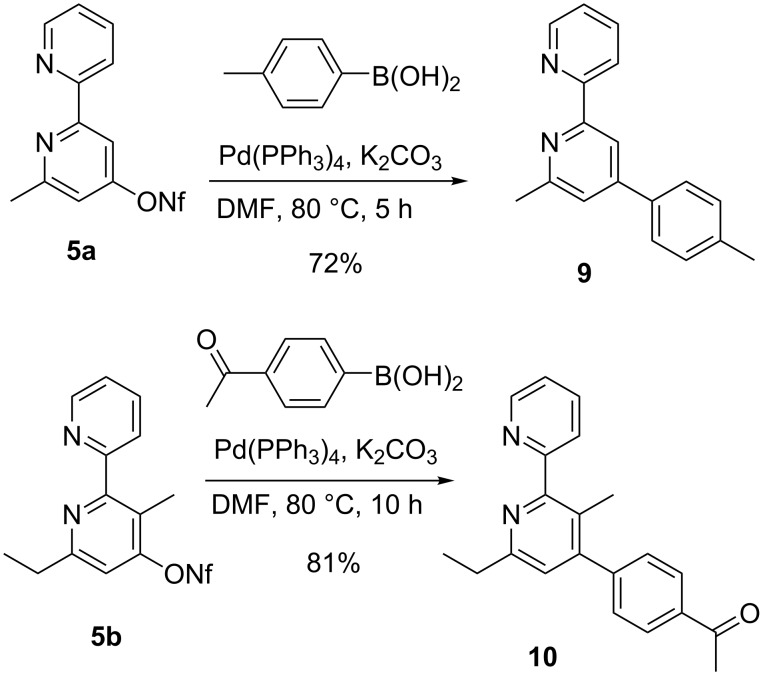
Suzuki-couplings of 2,2'-bipyrid-4-yl nonaflates **5a** and **5b** to compounds **9** and **10**.

The 2,2´-bipyridyl nonaflates prepared by the methods described above are excellent and versatile precursors for transition-metal-catalyzed coupling reactions [32,53–57]. Standard conditions for a Suzuki coupling smoothly transform compound **5a** and a boronic acid derivative into the expected 4-tolyl-substituted derivative **9** and the higher substituted precursor **5b** was converted into compound **10** by employing an acetyl-substituted boronic acid derivate in very good yield (Scheme 5). Compound **10** was prepared in order to investigate its capability to form a *C*_3_-symmetric star-shaped product by cyclocondensation of the acetyl group. This type of aldol condensation that generates a new benzene ring from three acetyl groups was successfully applied in our group in other cases delivering compounds whose 2D self-assembly at highly ordered pyrolytic graphite was studied by STM methods [58–59]. However, in the case of compound **10** no full conversion to the desired star-shaped compound was observed under the applied reaction conditions (SiCl_4_, EtOH) [60–61], very likely due to the insolubility of the intermediates involved in this multistep process.

**Scheme 6:**
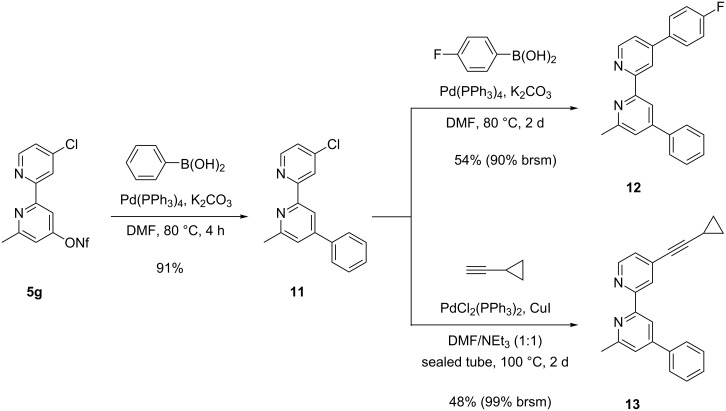
Palladium-catalyzed couplings of chloro-substituted 2,2'-bipyrid-4-yl nonaflate **5g** leading to compounds **11**, **12** and **13**.

The chloro-substituted 2,2´-bipyridine derivative **5g** – available due to the accidental chlorination of the picolinic acid precursor (Scheme 2) – offered the option to perform two subsequent coupling reactions in a controlled fashion. In the first step, the more reactive nonaflate moiety of **5g** was efficiently substituted by a phenyl group under standard Suzuki reaction conditions (Scheme 6). The untouched chloro substituent in the second pyridine ring was then replaced by another aryl substituent leading to product **12** in 54% yield. Alternatively, a Sonogashira reaction with (cyclopropyl)ethyne transformed intermediate **11** into the alkynyl-substituted 2,2´-bipyridine derivative **13**. Again the yield of this second coupling step is only moderate since the chloro compound **11** is not very reactive; in both cases even after two days reaction time ca. 50% of the precursor was re-isolated. Nevertheless, the examples in Scheme 5 and Scheme 6 clearly demonstrate that by suitable subsequent reactions our library of unsymmetrically substituted 2,2´-bipyridine derivatives **5** can easily be enlarged in a flexible manner.

## Conclusion

In this report we could show that the scope of the cyclocondensation reaction of β-ketoenamides **3** leading to unsymmetrically substituted bipyridine derivatives **5** is fairly broad and compatible with various functional groups. Only substrates such as **3h** having no “superfluous” substituents did not provide the expected bipyridine skeleton. Substrates **3** with higher substitution degree provided the expected bipyridine derivatives that were directly converted into the corresponding nonafloxy-substituted compounds **5** in good overall yield. These are excellent precursors for palladium-catalyzed coupling reactions as exemplified by the synthesis of compounds such as **9**–**13**. By an attempt to prepare the methoxy-substituted β-ketoenamide **3i** by oxidative methoxylation of compound **3a** we unexpectedly observed the formation of the 5-acetyl-substituted oxazole derivative **6**. However, precursor **3i** was smoothly available from acetylacetone **1a**. It could smoothly be transformed into the desired bipyridine derivative **4i**, but also into the related pyrimidine and pyrimidine *N*-oxide derivatives **7** and **8**. Altogether, we could confirm that our approach to functionalized pyridine derivatives by cyclocondensation of β-ketoenamides is also a very versatile and flexible method for the synthesis of unsymmetrically substituted bipyridine derivatives or related heterocyclic compounds that are not accessible by alternative methods.

## Supporting Information

File 1 General information, all experimental procedures and analytical data as well as copies of NMR spectra of all compounds.
