# Supplementary material for: Synthesis of highly functionalized 2,2'-bipyridines by cyclocondensation of β-ketoenamides – scope and limitations
Source: Beilstein J Org Chem. 2016 Jun 9;12:1170–7. doi: 10.3762/bjoc.12.112 (PMC4979911; doi:10.3762/bjoc.12.112)

Supporting Information

for

Synthesis of highly functionalized 2,2'-bipyridines by cyclocondensation of β-ketoenamides – scope and limitations

Paul Hommes[1] and Hans-Ulrich Reissig*[1]

Address: [1]Freie Universität Berlin, Institut für Chemie und Biochemie, Takustrasse 3, D-14195 Berlin, Germany

Email: Hans-Ulrich Reissig* - hreissig@chemie.fu-berlin.de

*Corresponding author

General information, all experimental procedures and analytical data as well as copies of NMR spectra of all compounds

Contents

1) General information s2

2) Experimental procedures s3

3) References. s25

4) ^{1}H and ^{13}C NMR spectra s26

1) General information

Reactions were generally performed under an atmosphere of argon in flame-dried flasks. Solvents and liquid reagents were added by syringe (polypropylene) or Eppendorf pipettes. Dichloromethane, THF, and toluene were purified with the MB SPS-800-dry solvent system. 1,2-Dichloroethane (DCE) was stored over activated 4 Å molecular sieves (freshly dried at 350 °C under vacuum). Ethanol was distilled from sodium and stored with activated 4 Å molecular sieves. DMF was purchased in p. A. quality (stored under an atmosphere of nitrogen with 4 Å molecular sieves). NEt_3 was distilled from CaH_2 and stored over KOH under an atmosphere of argon. NaH was purchased as a suspension in mineral oil (60%) and was washed with hexane under an atmosphere of argon prior to use. Hexanes were distilled from CaH_2, EtOAc was distilled from K_2CO_3 and $CaCl_2$. 3-Phenylpentane-2,4-dione (**1c**),[1] 3-benzylpentane-2,4-dione (**1d**),[2] enaminoketone **2a**,[3] (*Z*)-4-amino-3-phenylpent-3-en-2-one (**2c**),[4] (*Z*)-ethyl 2-amino-4-oxopent-2-enoate (**2e**),[5] 2-PyCOBt[6], PhIO[7] and 6-(methoxycarbonyl)picolinic acid[8] were synthesized according literature procedures. For the synthesis of 2,2-bipyrid-4-yl nonaflate **5b** see ref [9]. Unless noted otherwise, all other reagents and solvents were used as received by commercial suppliers.

Thin layer chromatography (TLC) analyses were performed on TLC plates purchased from Merck (silica gel 60, fluorescence indicator F254, 0.25 mm layer thickness). Products were purified by flash chromatography on silica gel 60 (230–400 mesh, Macherey-Nagel). NMR spectra were recorded with Bruker (AC 500, AVIII 700) and JEOL (ECX 400, Eclipse 500) instruments. Chemical shifts are reported relative to solvent residual peaks or TMS [^{1}H NMR: δ = 0.00 ppm (TMS), 2.50 ppm (DMSO-d_6), 3.31 ppm (CD_3OD), 7.26 ppm ($CDCl_3$); ^{13}C NMR: δ = 39.5 ppm (DMSO-d_6), 49.0 ppm (CD_3OD), 77.0 ppm ($CDCl_3$). Integrals are in accordance with assignments, and coupling constants are given in Hz. All ^{13}C NMR spectra are proton-decoupled. ^{13}C NMR signals of C_4F_9 groups are not listed: an unambiguous assignment was not possible due to very strong splitting of signals caused by coupling with the ^{19}F nuclei. For detailed peak assignments 2D spectra were measured (COSY, HMQC, HMBC). IR spectra were measured with a Jasco FT/IR-4100 spectrometer. HRMS analyses were performed with Varian Ionspec QFT-7 (ESI–FT ICRMS), Agilent 6210 (ESI–TOF) or Finnigan MAT 711 (EI, 80 eV, 8 kV) instruments. Elemental analyses were carried out with CHN-Analyzer Vario EL or Vario EL III instruments. Melting points were measured with a Reichert apparatus (Thermovar) and are uncorrected.

2) Experimental procedures

TMSOTf-promoted cyclization of β-ketoenamides followed by nonaflation of the crude product (GP-1):

In a similar manner as described before[10], TMSOTf (5 equiv) was added dropwise to a stirred solution of the β-ketoenamide (1 equiv) and DIPEA (4 equiv) in DCE (0.04 M) under an atmosphere of argon. The mixture was stirred at 90–100 °C in a sealed tube or under reflux for 3 d. After cooling to rt the solvent was evaporated carefully under reduced pressure. The remaining residue was dissolved in THF (0.05–0.10 M) under an atmosphere of argon. NaH (5–7 equiv, 60% in mineral oil) was washed twice with hexanes under an atmosphere of argon and suspended in a small amount of THF. This suspension was slowly transferred (*slowly! – vigorous* H_2*-evolution*) to the solution of the crude hydroxypyridine (or vice versa). NfF (2.5-3.0 equiv) or Nf_2O (1.1-1.5 equiv) was added and the mixture was stirred at rt overnight. Sat. aq. NH_4Cl solution was added (*slowly!*) and the layers were separated. The aqueous layer was extracted three times with CH_2Cl_2 (or Et_2O) and the combined organic layers were dried with Na_2SO_4, filtered and evaporated under reduced pressure. The obtained crude product was purified by flash column chromatography on silica gel.

Palladium-catalyzed coupling of 2,2'-bipyrid-4-yl nonaflates with boronic acids (Suzuki–Miyaura-coupling) (GP-2):

In a similar manner as described before[10], DMF (3-5 mL/mmol of substrate) was purged with argon for 30 min. The solvent was then transferred to a Schlenk-tube that was charged with the nonaflate (1 equiv), $Pd(PPh_3)_4$ (5–10 mol%), K_2CO_3 (1 equiv) and the boronic acid (1.0–1.1 equiv) under an atmosphere of argon. The mixture was stirred at 80 °C for 2–20 h. After cooling to rt the mixture was diluted with EtOAc and washed three times with an equal volume of a mixture of water and brine (1:1). The organic layer was dried with Na_2SO_4, filtered and evaporated under reduced pressure. The obtained crude product was purified by flash column chromatography.

(*Z*)-4-Amino-3-benzylpent-3-en-2-one (2d):

A solution of diketone **1d** (1.22 g, 6.41 mmol) in NH_3 (7 M in MeOH, 7.3 mL) was stirred at rt for 1 d. All volatile components were removed under reduced pressure. The obtained crude product was purified by flash column chromatography on silica gel (hexanes/EtOAc = 2:1) to provide β-ketoenamine **2d** (686 mg, 57%) as a pale yellow solid and a mixture of starting material **1d** and 4-phenylbutan-2-one (296 mg, **2d**/4-phenylbutan-2-one = 30:70). For the

elemental analysis, a sample of **2d** was recrystallized by diffusion of hexanes into a solution of **2d** in $CHCl_3$.

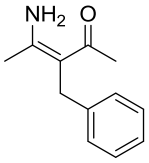

2d

M.p.: 69-72 °C.

^{1}H NMR ($CDCl_3$, 500 MHz): δ = 1.91, 2.05 (2 s, 3 H each, CH_3), 3.66 (s, 2 H, CH_2), 5.17 (s_{br}, 1 H, NH), 7.15-7.28 (m, 5 H, Ph), 10.67 (s_{br}, 1 H, NH) ppm.

^{13}C NMR ($CDCl_3$, 126 MHz): δ = 21.6, 28.1 (2 q, CH_3), 34.1 (t, CH_2), 102.2 (s, C-3), 125.8, 127.4, 128.4, 141.3 (3 d, s, Ph), 160.7 (s, CNH_2), 197.9 (s, C=O) ppm.

IR (ATR): ν = 3275, 3115 (N-H), 3085-3025 (=C-H), 2995, 2925, 2860 (C-H), 1590, 1490, 1545 (C=C, C=O), 1450, 1345, 1220, 1185 cm^{-1}.

HRMS (ESI-TOF): $C_{12}H_{16}NO$ ($M+H^+$), calcd.: 190.1226; found: 190.1234.

EA: $C_{12}H_{15}NO$ (189.3), calcd. (%): C 76.16, H 7.99, N 7.40; found (%): C 75.70, H 7.82, N 7.32.

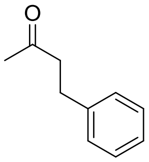

^{1}H NMR ($CDCl_3$, 250 MHz): δ = 2.12 (s, 3 H, CH_3), 2.71-2.78, 2.85-2.91 (2 m, 2 H each, CH_2), 7.15-7.29 (m, 5 H, Ph) ppm.

The ^{1}H-NMR data are in agreement with those reported in the literature.[11]

(*E*)-4-Amino-3-methoxypent-3-en-2-one (2i):

$BF_3 \cdot OEt_2$ (1.37 mL, 10.9 mmol) was added dropwise to a suspension of PhIO (2.40 g, 10.9 mmol) in MeOH (44 mL) under an atmosphere of argon. The mixture was stirred at rt for 5 min to give a clear solution. Acetyl acetone (**1a**, 1.12 mL, 10.9 mmol) was added and stirring was continued for 5 h. The mixture was concentrated under reduced pressure (>100 mbar), diluted with sat. aq. $NaHCO_3$ solution (50 mL) and extracted with CH_2Cl_2 (3 × 50 mL). The combined organic layers were dried with Na_2SO_4, filtered and evaporated under reduced pressure (>100 mbar). The remaining residue was dissolved in NH_3 (12 mL, 7 M in MeOH), stirred at rt for 3 h and evaporated again under reduced pressure. The obtained crude

product was purified by flash column chromatography on silica gel (hexanes/EtOAc = 1:1) to provide β-ketoenamine **2i** (709 mg, 50%) as a brown oil.

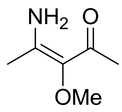

2i

^{1}H NMR ($CDCl_3$, 500 MHz): δ = 1.96, 2.14 (2 s, 3 H each, CH_3), 3.45 (s, 3 H, OCH_3), 4.69, 9.05 (2 s_{br}, 1 H each, NH) ppm.

^{13}C-NMR ($CDCl_3$, 126 MHz): δ = 17.3, 25.2 (2 q, CH_3), 61.5 (q, OCH_3), 132.8 (s, C-3), 153.2 (s, C-4), 195.4 (s, C=O) ppm.

IR (ATR): ν = 3350, 3175 (N-H), 2980, 2930, 2825 (C-H), 1620 (C=O), 1485, 1355, 1280, 1220 cm^{-1}.

HRMS (ESI-TOF): $C_6H_{11}NO_2Na^+$: calcd.: 152.0682; found: 152.0687.

(*Z*)-*N*-(4-Oxopent-2-en-2-yl)picolinamide (3a):

NEt_3 (90 µL, 0.65 mmol) and BOP (150 mg, 0.34 mmol) were added to a mixture of the picolinic acid (37 mg, 0.30 mmol) and β-ketoenamine **2a** (30 mg, 0.30 mmol) in CH_2Cl_2 under an atmosphere of argon. The mixture was stirred at rt for 20 h and then diluted with CH_2Cl_2 (20 mL), washed with aq. HCl (20 mL, 1 M) and sat. aq. $NaHCO_3$ solution (20 mL). The organic layer was dried with Na_2SO_4, filtered and concentrated under reduced pressure. The obtained crude product was purified by flash column chromatography on silica gel (hexanes/EtOAc = 4:1) to provide β-ketoenamide **3a** (45 mg, 73%) as a colorless solid.

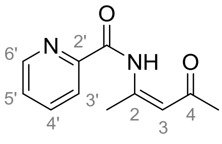

3a

M.p.: 106-107 °C.

^{1}H NMR ($CDCl_3$, 250 MHz): δ = 2.18 (s, 3 H, 2-CH_3), 2.52 (d, J = 0.9 Hz, 3 H, 4-CH_3), 5.47 (d, J = 0.9 Hz, 1 H, 3-H), 7.46 (ddd, J = 7.6, 4.8, 1.2 Hz, 1 H, 5'-H), 7.85 (td, J ≈ 7.7, 1.7 Hz, 1 H, 4'-H), 8.16 (dt, J ≈ 7.9, 1.0 Hz, 1 H, 3'-H), 8.75 (ddd, J = 4.8, 1.7, 0.9 Hz, 1 H, 6'-H), 13.95 (s_{br}, 1 H, NH) ppm.

^{13}C NMR ($CDCl_3$, 126 MHz): δ = 21.9 (q, 4-CH_3), 30.4 (q, 2-CH_3), 107.2 (d, C-3), 123.0 (d, C-3'), 126.7 (d, C-5'), 137.3 (d, C-4'), 148.8 (d, C-6'), 149.7, 153.4 (2 s, C-2, C-2'), 164.3 (s, CONH), 199.2 (s, C=O) ppm.

The analytical data are in agreement with those reported in the literature.[9]

(*Z*)-Methyl 6-(4-oxo-3-phenylpent-2-en-2-ylcarbamoyl)picolinate (3c):

6-(Methoxycarbonyl)picolinic acid[8] (318 mg, 1.76 mmol) was suspended in $SOCl_2$ (4 mL) in a two-neck flask equipped with a reflux condenser and a drying tube that was charged with $CaCl_2$. The mixture was heated to 80 °C under reflux for 30 min. to give a clear solution. Excess $SOCl_2$ was removed under reduced pressure and collected with a cooling trap (liquid N_2). The remaining residue was re-dissolved in CH_2Cl_2 (5 mL) and transferred at 0 °C to a solution of β-ketoenamine **2c** (280 mg, 1.60 mmol) and NEt_3 (0.36 mL, 2.59 mmol) in CH_2Cl_2 (5 mL) under an atmosphere of argon. The mixture was stirred over night while it was allowed to warm up to rt, then sat. aq. $NaHCO_3$ solution (15 mL) was added slowly and the layers were separated. The aqueous layer was extracted with CH_2Cl_2 (3 × 15 mL). The combined organic layers were dried with Na_2SO_4, filtered and evaporated under reduced pressure. The obtained crude product was purified flash column chromatography on silica gel (hexanes/EtOAc = 8:1→3:1) to provide β-ketoenamide **3c** (421 mg, 78%) as a colorless solid. For the elemental analysis, a sample of **3c** was recrystallized from hexanes/EtOAc (2:1).

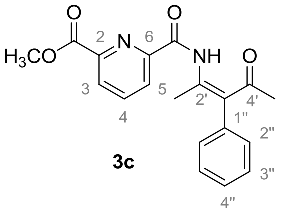

3c

M.p.: 130-132 °C.

^{1}H NMR ($CDCl_3$, 500 MHz): δ = 1.98 (s, 3 H, 4'-CH_3), 2.29 (s, 3 H, 2'-CH_3), 4.11 (s, 3 H, OCH_3), 7.18-7.21, 7.33-7.42 (2 m, 2 H, 3 H, Ph), 8.05 (t, *J* = 7.8 Hz, 1 H, 4-H), 8.31 (d_{br}, *J* = 7.8 Hz, 1 H, 3-H), 8.38 (d_{br}, *J* = 7.8 Hz, 1 H, 5-H) ppm; the NH signal was not detected.

^{13}C NMR ($CDCl_3$, 126 MHz): δ = 19.3 (q, 2'-CH_3), 31.4 (q, 4'-CH_3), 53.2 (q, OCH_3), 121.4 (s, C-3'), 126.0 (d, C-3), 127.5, 127.6 (2 d, C-5, C-4''), 128.9, 130.8 (2 d, C-2'', C-3''), 138.6 (s, C-1''), 138.7 (d, C-4), 147.0 (s, C-2), 150.3, 150.5 (s, C-6, C-2'), 163.7 (s, CONH), 164.9 (s, *C*O_2CH_3), 201.2 (s, C-4') ppm.

IR (ATR): ν = 3045 (=C-H), 2955 (C-H), 1750, 1720, 1690 (C=O), 1630, 1565 (C=C, C=N), 1465-1415, 1355, 1245-1135 cm^{-1}; the NH signal was not detected.

HRMS (ESI-TOF): $C_{19}H_{18}N_2O_4 \cdot Na^+$: calcd.: 361.1159; found: 361.1156.

EA: $C_{19}H_{18}N_2O_4$ (338.4) calcd. (%): C 67.44, H 5.36, N 8.28; found (%): C 67.37, H 5.34, N 8.28.

(*Z*)-*N*-(3-Benzyl-4-oxopent-2-en-2-yl)picolinamide (3d):

Picolinic acid (450 mg, 3.66 mmol), NEt_3 (0.50 mL, 3.60 mmol.) and catalytic amounts of DMF (~3 drops) were dissolved in CH_2Cl_2 (10 mL) under an atmosphere of argon and $SOCl_2$ (0.27 mL, 3.71 mmol) was added dropwise. The solution was stirred at rt for 4 h and then slowly transferred to a solution of the β-ketoenamine **2d** (346 mg, 1.83 mmol) and NEt_3 (0.50 mL, 3.60 mmol) in CH_2Cl_2 (5 mL) at 0 °C. The mixture was stirred for 20 h while being allowed to warm up to rt and then diluted slowly with sat. aq. $NaHCO_3$ solution (30 mL). The layers were separated and the aqueous layer was extracted with CH_2Cl_2 (3 × 30 mL). The combined organic layers were dried with Na_2SO_4, filtered and evaporated under reduced pressure. The obtained crude product was purified by flash column chromatography on silica gel (hexanes/EtOAc = 4:1) to provide β-ketoenamide **3d** (380 mg, 71%) as a colorless solid. For the elemental analysis, a sample of **3d** was recrystallized by diffusion of hexanes into a solution of **3d** in $CHCl_3$.

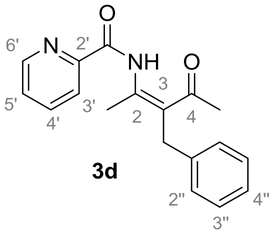

3d

M.p.: 103-104 °C.

^{1}H NMR ($CDCl_3$, 500 MHz): δ = 2.21 (s, 3 H, 4-CH_3), 2.58 (s, 3 H, 2-CH_3), 3.86 (s, 2 H, CH_2), 7.16-7.24 (m, 3 H, 3''-H, 4''-H), 7.28-7.33 (m, 2 H, 2''-H), 7.47 (ddd, *J* = 7.6, 4.8, 1.2 Hz, 1 H, 5'-H), 7.87 (td, *J* ≈ 7.7, 1.7 Hz, 1 H, 4'-H), 8.21 (m_c, *J* ≈ 7.9, 1.0 Hz, 1 H, 3'-H), 8.79 (ddd, *J* = 4.8, 1.7, 0.9 Hz, 1 H, 6'-H) ppm; the NH signal was not detected.

^{13}C NMR ($CDCl_3$, 126 MHz): δ = 17.6 (q, 2-CH_3), 29.7 (q, 4-CH_3), 34.1 (t, CH_2), 114.9 (s, C-3), 123.0 (d, C-3'), 126.4, 126.5 (2 d, C-5', C-4''), 127.5 (d, C-2''), 128.7 (d, C-3''), 137.3 (d, C-4'), 139.0 (s, C-1''), 148.8 (d, C-6'), 150.4 (s, C-2'), 151.6 (s, C-2), 164.4 (s, CONH), 202.1 (s, C-4) ppm.

IR (ATR): ν = 3165-2935 (N-H, =C-H, C-H), 1690, 1630 (C=O), 1560 (C=C, C=N), 1475, 1450, 1425, 1245, 1200 cm^{-1}.

HRMS (ESI-TOF): $C_{18}H_{18}N_2O_2 \cdot Na^+$: calcd.: 317.1260; found: 317.1271.

EA: $C_{18}H_{18}N_2O_2$ (294.3) calcd. (%): C 73.45, H 6.16, N 9.52; found (%): C 73.30, H 6.17, N 9.43.

(*Z*)-Ethyl 4-oxo-2-(picolinamido)pent-2-enoate (3e):

KHMDS (5.20 mL, 3.64 mmol, 0.7 M in toluene) was added to a solution of the β-ketoenamine **2e** (440 mg, 2.80 mmol) in THF (25 mL) under an atmosphere of argon. 2-PyCOBt (690 mg, 3.08 mmol) was added and the mixture was stirred at rt for 1 d. Brine (30 mL) was added and the layers were separated. The aqueous layer was extracted with CH_2Cl_2 (3 × 30 mL) and the combined organic layers were dried with Na_2SO_4, filtered and evaporated under reduced pressure. The obtained crude product was purified by flash column chromatography on silica gel (hexanes/EtOAc = 5:1, 4:1) to provide recovered starting material **2e** (95 mg, 22%) and β-ketoenamide **3e** (244 mg, 33%) as a pale yellow solid.

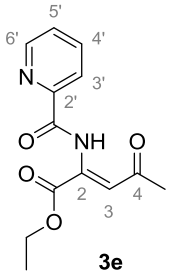

3e

M.p.: 75-76 °C.

^{1}H NMR ($CDCl_3$, 500 MHz): δ = 1.37 (t, *J* = 7.2 Hz, 3 H, CH_2CH_3), 2.30 (s, 3 H, $COCH_3$), 4.40 (q, *J* = 7.2 Hz, 2 H, OCH_2), 5.89 (s, 1 H, 3-H), 7.50 (ddd, *J* = 7.6, 4.8, 1.2 Hz, 1 H, 5'-H), 7.87 (td, *J* ≈ 7.7, 1.7 Hz, 1 H, 4'-H), 8.16 (m_c, *J* ≈ 7.7, 1.0 Hz, 1 H, 3'-H), 8.73 (ddd, *J* = 4.8, 1.7, 0.9 Hz, 1 H, 6'-H), 13.0 (s_{br}, 1 H, NH) ppm.

^{13}C NMR ($CDCl_3$, 126 MHz): δ = 13.9 (q, CH_2CH_3) 31.1 (q, $COCH_3$), 62.4 (t, OCH_2), 107.9 (d, C-3), 123.2 (d, C-3'), 127.2 (d, C-5'), 137.4 (d, C-4'), 141.5 (s, C-2), 148.2 (s, C-2'), 150.0 (d, C-6'), 163.0 (s, CONH), 164.4 (s, CO_2Et), 199.9 (s, CO) ppm.

IR (ATR): ν = 3245 (N-H), 3060 (=C-H), 2990, 2935, 2900 (C-H), 1735, 1700, 1665 (C=O), 1590 (C=C, C=N), 1475-1460, 1290-1265 cm^{-1}.

EA: $C_{13}H_{14}N_2O_4$ (262.3) calcd. (%): C 59.54, H 5.38, N 10.68; found (%): C 59.62, H 5.41, N 10.69.

(*Z*)-4-Chloro-*N*-(4-oxopent-2-en-2-yl)picolinamide (3f):

$SOCl_2$ (15.0 mL, 206 mmol) was stirred at 50 °C in a two-neck flask equipped with a reflux condenser and a drying tube that was charged with $CaCl_2$. DMF (0.5 mL) was added dropwise and the mixture was stirred for 15 min. After slow addition of picolinic acid (5.00 g, 40.6 mmol) the mixture was heated to 75 °C under reflux for 20 h. The initially green color of

the mixture turned quickly to dark violet and changed again to an orange color during that time. Excess $SOCl_2$ was removed under reduced pressure and collected with a cooling trap (liquid N_2). Toluene (20 mL) was added to the remaining residue and the solvent was again removed under reduced pressure. The remaining residue was then re-dissolved in CH_2Cl_2 (50 mL) and transferred at 0 °C to a solution of β-ketoenamine **2a** (4.03 g, 40.6 mmol) and NEt_3 (14 mL, 101 mmol) in CH_2Cl_2 (110 mL) under an atmosphere of argon. The mixture was stirred over night while it was allowed to warm up to rt Sat. aq. $NaHCO_3$ solution (50 mL) was added slowly and the layers were separated. The aqueous layer was extracted with CH_2Cl_2 (3 × 50 mL). The combined organic layers were dried with Na_2SO_4, filtered and evaporated under reduced pressure. The obtained crude product was purified by two runs of flash column chromatography on silica gel (hexanes/EtOAc = 10:1→4:1) to provide β-ketoenamide **3f** (4.94 g, 51%) and β-ketoenamide **3a** (2.24 g, 27%) as colorless solids.

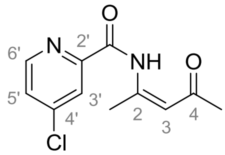

3f

M.p.: 138-140 °C.

^{1}H NMR ($CDCl_3$, 500 MHz): δ = 2.20 (s, 3 H, 2-CH_3), 2.52 (d, *J* = 0.9 Hz, 3 H, 4-CH_3), 5.50 (d, *J* = 0.9 Hz, 1 H, 3-H), 7.48 (dd, *J* = 5.2, 2.1 Hz, 1 H, 5'-H), 8.18 (d, *J* = 2.1 Hz, 1 H, 3'-H), 8.66 (d, *J* = 5.2 Hz, 1 H, 6'-H) ppm; the NH signal was not detected.

^{13}C NMR ($CDCl_3$, 126 MHz): δ = 21.9 (q, 4-CH_3), 30.5 (q, 2-CH_3), 107.6 (d, C-3), 123.6 (d, C-3'), 126.9 (d, C-5'), 145.8 (s, C-4'), 149.7 (d, C-6'), 151.2 (s, C-2'), 153.2 (s, C-2), 163.1 (s, CONH), 199.4 (s, C=O) ppm.

IR (ATR): ν = 3115-3090 (=C-H), 3000-2920 (C-H), 1690, 1655 (C=O), 1600, 1570, 1555 (C=C, C=N), 1490, 1350, 1255 cm^{-1}.

HRMS (ESI-TOF): $C_{11}H_{11}ClN_2O_2 \cdot Na^+$: calcd.: 261.0407; found: 261.0403.

EA: $C_{11}H_{11}ClN_2O_2$ (238.7) calcd. (%): C 55.36, H 4.65, N 11.74; found (%): C 55.27, H 4.78, N 11.52.

Methyl (*Z*)-6-(4-oxopent-2-en-2-ylcarbamoyl)picolinate (3g):

6-(Methoxycarbonyl)picolinic acid[8] (1.00 g, 5.52 mmol) was suspended in $SOCl_2$ (4 mL) in a two-neck flask equipped with a reflux condenser and a drying tube that was charged with $CaCl_2$. The mixture was heated to 80 °C under reflux for 30 min. to give a clear solution. Excess $SOCl_2$ was removed under reduced pressure and collected with a cooling trap (liquid

N_2). The remaining residue was re-dissolved in CH_2Cl_2 (15 mL) and transferred at 0 °C to a solution of β-ketoenamine **2a** (547 mg, 5.52 mmol) and NEt_3 (1.15 mL, 8.28 mmol) in CH_2Cl_2 (10 mL) under an atmosphere of argon. The mixture was stirred over night while it was allowed to warm up to rt Sat. aq. $NaHCO_3$ solution (30 mL) was added slowly and the layers were separated. The aqueous layer was extracted with CH_2Cl_2 (3 × 30 mL). The combined organic layers were dried with Na_2SO_4, filtered and evaporated under reduced pressure. The obtained crude product was purified flash column chromatography on silica gel (hexanes/EtOAc = 2:1) to provide β-ketoenamide **3g** (1.33 g, 92%) as a colorless solid.

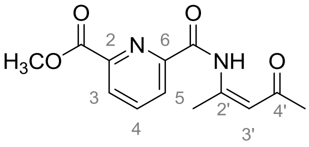

3g

M.p.: 116-117 °C.

^{1}H NMR ($CDCl_3$, 500 MHz): δ = 2.21 (s, 3 H, 2'-CH_3), 2.52 (d, *J* = 1.0 Hz, 3 H, 4'-CH_3), 4.10 (s, 3 H, OCH_3), 5.51 (d, *J* = 1.0 Hz, 1 H, 3'-H), 8.04 (t, *J* = 7.8 Hz, 1 H, 4-H), 8.30 (dd, *J* = 1.1, 7.8 Hz, 1 H, 5-H), 8.36 (dd, *J* = 1.1, 7.8 Hz, 1 H, 3-H), 14.08 (s, 1 H, NH) ppm.

^{13}C NMR ($CDCl_3$, 126 MHz): δ = 21.8 (q, 2'-CH_3), 30.6 (q, 4'-CH_3), 53.2 (q, OCH_3), 107.8 (d, C-3'), 126.0 (d, C-5), 127.7 (d, C-3), 138.7 (d, C-4), 147.0 (s, C-6), 149.9 (s, C-2), 152.7 (s, C-2'), 163.3 (s, CONH), 164.7 (s, *C*$OOCH_3$), 199.4 (s, C-4') ppm.

IR (ATR): ν = 3135–2995 (=C-H), 2955 (C-H), 1720 (C=O), 1695, 1650, 1600, 1585, 1490, 1430 (C=C, C=N), 1355, 1320, 1255 cm^{-1}.

HRMS (ESI-TOF): $C_{13}H_{14}N_2O_4{\cdot}Na^+$: calcd.: 285.0872; found: 285.0851.

EA: $C_{13}H_{14}N_2O_4$ (262.3) calcd. (%): C 59.54, H 5.38, N 10.68; found (%): C 59.52, H 5.33, N 10.72.

(*Z*)-*N*-(3-Oxobut-1-enyl)picolinamide (3h):

A mixture of picolinamide (293 mg, 2.40 mmol) and but-3-yn-2-one (0.16 mL, 2.04 mmol) was dissolved in MeOH (5 mL) and stirred at 65 °C for 1 d in a sealed tube. All volatile components were removed under reduced pressure. The obtained crude product was purified by flash column chromatography on silica gel (hexanes/EtOAc = 8:1→1:1) to provide β-ketoenamide **3h** (82 mg, 22%) as a colorless solid and re-isolated picolinamide (134 mg, 46%). For the elemental analysis, a sample of **3h** was recrystallized from hexanes/EtOAc (2:1).

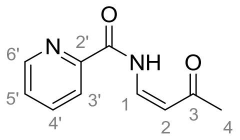

3h

M.p.: 86-88 °C.

^{1}H NMR ($CDCl_3$, 700 MHz): δ = 2.24 (s, 3 H, 4-H), 5.67 (d, *J* = 8.6 Hz, 1 H, 2-H), 7.49 (ddd, *J* = 7.6, 4.7, 1.2 Hz, 1 H, 5'-H), 7.54 (dd, *J* = 11.7, 8.6 Hz, 1 H, 1-H), 7.87 (td, *J* ≈ 7.7, 1.7 Hz, 1 H, 4'-H), 8.21 (m_c, *J* ≈ 7.8, 1.0 Hz, 1 H, 3'-H), 8.72 (ddd, *J* = 4.7, 1.7, 0.9 Hz, 1 H, 6'-H), 13.16 (d_{br}, *J* ≈ 12 Hz, 1 H, NH) ppm.

^{13}C NMR ($CDCl_3$, 176 MHz): δ = 30.7 (q, C-4), 105.6 (d, C-2), 123.2 (d, C-3'), 127.1 (d, C-5'), 135.9 (d, C-1), 137.3 (d, C-4'), 148.3 (s, C-2'), 149.0 (d, C-6'), 163.8 (s, CONH), 200.5 (s, C-3) ppm.

IR (ATR): ν = 3390 (N-H), 3070, 3000 (=C-H), 2960-2850 (C-H), 1700, 1660 (C=O), 1570, 1475-1465 (C=C, C=N), 1440, 1385-1265 cm^{-1}.

HRMS (ESI-TOF): $C_{10}H_{10}N_2O_2 \cdot Na^+$: calcd.: 213.0635; found: 213.0638.

EA: $C_{10}H_{10}N_2O_2$ (190.2) calcd. (%): C 63.15, H 5.30, N 14.73; found (%): C 63.07, H 4.89, N 14.73.

(*E*)-*N*-(3-Methoxy-4-oxopent-2-en-2-yl)picolinamide (3i):

KHMDS (2.06 mL, 1.44 mmol, 0.7 M in toluene) was added to a solution of β-ketoenamine **2i** (124 mg, 0.96 mmol) in THF (10 mL) under an atmosphere of argon. 2-PyCOBt (238 mg, 1.06 mmol) was added and the mixture was stirred at rt for 2 d. Brine (30 mL) was added and the layers were separated. The aqueous layer was extracted with CH_2Cl_2 (3 × 30 mL) and the combined organic layers were dried with Na_2SO_4, filtered and evaporated under reduced pressure. The obtained crude product was purified by flash column chromatography on silica gel (hexanes/EtOAc = 4:1) to provide β-ketoenamide **3i** (113 mg, 50%) as a yellow solid.

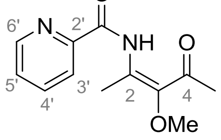

3i

M.p.: 59-61 °C.

^{1}H NMR ($CDCl_3$, 500 MHz): δ = 2.33 (s, 3 H, 4-CH_3) 2.61 (s, 3 H, 2-CH_3), 3.60 (s, 3 H, OCH_3), 7.54 (ddd, *J* = 7.6, 4.8, 1.1 Hz, 1 H, 5'-H), 7.85 (td, *J* ≈ 7.7, 1.7 Hz, 1 H, 4'-H), 8.16

(m_c, $J \approx 7.8$, 0.9 Hz, 1 H, 3'-H), 8.73 (ddd, $J = 4.8$, 1.7, 0.8 Hz, 1 H, 6'-H), 13.5 (s_{br}, 1 H, NH) ppm.

^{13}C NMR ($CDCl_3$, 126 MHz): δ = 14.4 (q, 2-CH_3) 26.9 (q, 4-CH_3), 61.1 (q, OCH_3), 122.8 (d, C-3'), 126.5 (d, C-5'), 137.3 (d, C-4'), 139.2 (s, C-3), 143.6 (s, C-2), 148.7 (d, C-6'), 150.0 (s, C-2'), 164.5 (s, CONH), 200.0 (s, CO) ppm.

IR (ATR): ν = 3125 (N-H), 3050 (=C-H), 2935, 2835 (C-H), 1695, 1655 (CO), 1580, 1545 (C=C, C=N), 1480-1435, 1245 cm^{-1}.

HRMS (ESI-TOF): $C_{12}H_{14}N_2O_3 \cdot Na^+$: calcd.: 235.1077; found: 235.1122.

EA: $C_{12}H_{14}N_2O_3$ (234.3) calcd. (%): C 61.53, H 6.02, N 11.96; found (%): C 61.52, H 6.02, N 11.98.

(6-Methyl-2,2'-bipyridin-4-yl) nonaflate (5a):

According to **GP-1**, β-ketoenamide **3a** (400 mg, 1.96 mmol) was treated with DIPEA (1.33 mL, 7.83 mmol) and TMSOTf (1.78 mL, 9.79 mmol) in DCE (50 mL) for 3 d in a sealed tube, followed by nonaflation of the crude product using NaH (588 mg, 14.7 mmol) and NfF (1.06 mL, 5.88 mmol) in THF (20 mL). Workup was performed with sat. aq. NH_4Cl solution (20 mL) and CH_2Cl_2 (4 × 20 mL). The obtained crude product was purified by flash column chromatography on silica gel (hexanes/EtOAc = 20:1→15:1) to provide bipyridyl nonaflate **5a** (690 mg, 75%) as a colorless oil.

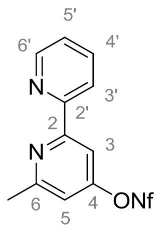

5a

^{1}H NMR ($CDCl_3$, 500 MHz): δ = 2.69 (s, 3 H, CH_3), 7.10 (d, $J = 2.2$ Hz, 1 H, 5-H), 7.35 (ddd, $J = 1.0$, 4.8, 7.8 Hz, 1 H, 5'-H), 7.83 (dt, $J = 1.8$, 7.8 Hz, 1 H, 4'-H), 8.20 (d, $J = 2.2$ Hz, 1 H, 3-H), 8.44 (td, $J = 1.0$ Hz, 7.8 Hz, 1 H, 3'-H), 8.69 (ddd, $J = 1.0$, 1.8, 4.8 Hz, 1 H, 6'-H) ppm.

^{13}C NMR ($CDCl_3$, 126 MHz): δ = 24.6 (q, CH_3), 110.5 (d, C-3'), 114.9, 121.3 (2 d, C-5', C-3), 124.4 (d, C-5), 136.9 (d, C-4'), 149.3 (d, C-6'), 154.4, 157.7, 158.9, 161.2 (4 s, C-2', C-2, C-4, C-6) ppm.

^{19}F NMR ($CDCl_3$, 471 MHz): δ = –125.7 (m_c, 2 F, CF_2), –120.7 (m_c, 2 F, CF_2), –108.7 (m_c, 2 F, CF_2), –80.7 (m_c, 3 F, CF_3) ppm.

IR (ATR): ν = 3095-3015 (=C-H), 2925 (C-H), 1600-1560 (C=C, C=N) cm^{-1}.

HRMS (ESI-TOF): $C_{15}H_9F_9N_2O_3S \cdot H^+$: calcd.: 469.0269; found: 469.0273.

Methyl 6'-methyl-4'-nonafloxy-5'-phenyl-2,2'-bipyridine-6-carboxylate (5c):

According to **GP-1**, β-ketoenamide **3c** (320 mg, 0.95 mmol) was treated with DIPEA (0.62 mL, 3.65 mmol) and TMSOTf (0.86 mL, 4.74 mmol) in DCE (25 mL) for 3 d in a sealed tube, followed by nonaflation of the crude product using NaH (227 mg, 5.68 mmol) and NfF (0.42 mL, 2.34 mmol) in THF (10 mL). Workup was performed with sat. aq. NH_4Cl solution (20 mL) and Et_2O (4 × 20 mL). The obtained crude product was purified by flash column chromatography on silica gel (hexanes/EtOAc = 10:1) to provide bipyridyl nonaflate **5c** (489 mg, 86%) as a pale yellow solid.

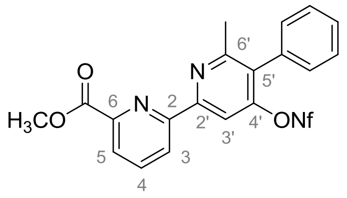

5c

M.p.: 84-86 °C.

^{1}H NMR ($CDCl_3$, 500 MHz): δ = 2.51 (s, 3 H, CH_3), 4.04 (s, 3 H, OCH_3), 7.29-7.31, 7.45-7.52 (2 m, 2 H, 3 H, Ph), 8.00 (dd, *J* = 7.9, 7.7 Hz, 1 H, 4-H), 8.19 (dd, *J* = 7.7, 0.6 Hz, 1 H, 5-H), 8.41 (s, 1 H, 3'-H), 8.66 (dd, *J* = 7.9, 0.6 Hz, 1 H, 3-H) ppm.

^{13}C NMR ($CDCl_3$, 126 MHz): δ = 23.9 (q, CH_3), 52.8 (q, OCH_3), 111.6 (d, C-3'), 124.4 (d, C-3), 125.6 (d, C-5), 128.7, 128.8, 129.7 (3 d, Ph), 130.4 (s, C-5'), 132.3 (s, Ph), 138.1 (d, C-4), 147.8, 154.7, 155.2, 156.2 (4 s, C-2, C-2', C-4', C-6), 160.2 (s, C-6'), 165.7 (s, C=O) ppm.

^{19}F NMR ($CDCl_3$, 471 MHz): δ = –125.8 (dt, *J* = 13.7, 4.2 Hz, 2 F, CF_2), –120.9 to –120.8 (m, 2 F, CF_2), –109.5 (t, *J* = 13.7 Hz, 2 F, CF_2), –80.6 (t, *J* = 9.6 Hz, 3 F, CF_3) ppm.

IR (ATR): ν = 3065 (=C-H), 2955 (C-H), 1720 (C=O), 1595-1545 (C=C, C=N), 1450, 1400, 1310, 1225-1140 cm^{-1}.

HRMS (ESI-TOF): $C_{23}H_{15}F_9N_2O_5S \cdot Na^+$: calcd.: 625.0450; found: 625.0429.

EA: $C_{23}H_{15}F_9N_2O_5S$ (602.4) calcd. (%): C 45.86, H 2.51, N 4.65, S 5.32; found (%): C 46.00, H 2.54, N 4.72, S 4.96.

(5-Benzyl-6-methyl-2,2'-bipyridin-4-yl) nonaflate (5d):

According to **GP-1**, β-ketoenamide **3d** (350 mg, 1.19 mmol) was treated with DIPEA (0.78 mL, 4.59 mmol) and TMSOTf (1.08 mL, 5.95 mmol) in DCE (30 mL) for 3 d under reflux, followed by nonaflation of the crude product using NaH (285 mg, 7.13 mmol) and NfF (0.53 mL, 2.95 mmol) in THF (15 mL). Workup was performed with sat. aq. NH_4Cl solution (20 mL) and CH_2Cl_2 (3 × 20 mL). The obtained crude product was purified by flash column chromatography on silica gel (hexanes/EtOAc = 10:1) to provide bipyridyl nonaflate **5d** (358 mg, 54%) as a yellow oil that solidified upon storage.

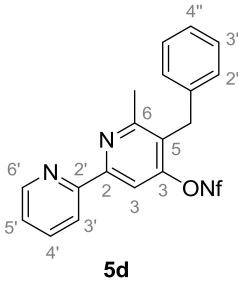

5d

M.p.: 39-40 °C.

^{1}H NMR ($CDCl_3$, 500 MHz): δ = 2.60 (s, 3 H, CH_3), 4.19 (s, 2 H, CH_2), 7.08-7.11 (m, 2 H, 3''-H), 7.22 (m_c, 1 H, 4''-H), 7.28 (m_c, 2 H, 2''-H), 7.33 (ddd, *J* = 7.5, 4.8, 1.2 Hz, 1 H, 5'-H), 7.82 (ddd, *J* = 8.0, 7.5, 1.8 Hz, 1 H, 4'-H), 8.35 (s, 1 H, 3-H), 8.44 (dt, *J* ≈ 7.6, 1.1 Hz, 1 H, 3'-H), 8.70 (ddd, *J* = 4.8, 1.8, 0.9 Hz, 1 H, 6'-H) ppm.

^{13}C NMR ($CDCl_3$, 126 MHz): δ = 23.3 (q, CH_3), 32.0 (t, CH_2), 110.9 (d, C-3), 121.1 (d, C-3'), 124.2 (d, C-5'), 126.2 (s, C-5), 126.6 (d, C-4''), 128.0 (d, C-3''), 128.7 (d, C-2''), 136.9 (d, C-4'), 137.2 (s, C-1''), 149.4 (d, C-6'), 154.5 (s, C-2'), 156.37, 156.39 (2 s, C-2, C-4), 161.1 (s, C-6) ppm.

^{19}F NMR ($CDCl_3$, 471 MHz): δ = –125.6, –120.6 (m_c, 2 F each, CF_2), –109.0 (t, *J* = 13.6 Hz, 2 F, CF_2), –80.5 (t, *J* = 9.7 Hz, 3 F, CF_3) ppm.

IR (ATR): ν = 3090, 3070, 3035 (=C-H), 2960-2925, 2865 (C-H), 1600-1550 (C=C, C=N), 1420, 1235, 1200, 1140 cm^{-1}.

HRMS (ESI-TOF): $C_{22}H_{15}F_9N_2O_3S \cdot Na^+$: calcd.: 581.0552; found: 581.0589.

EA: $C_{22}H_{15}F_9N_2O_3S$ (558.4) calcd. (%): C 47.32, H 2.71, N 5.02, S 5.74; found (%): C 47.32, H 2.70, N 4.90, S 5.71.

Ethyl 4-nonafloxy-2,2'-bipyridine-6-carboxylate (5e):

According to **GP-1**, β-ketoenamide **3e** (186 mg, 0.71 mmol) was treated with DIPEA (0.46 mL, 2.70 mmol) and TMSOTf (0.64 mL, 3.53 mmol) in DCE (18 mL) for 3 d in a sealed tube, followed by nonaflation of the crude product using NaH (170 mg, 4.25 mmol) and Nf_2O (0.24 mL, 0.78 mmol) in THF (10 mL). THF was removed under reduced pressure and the obtained crude product was purified by flash column chromatography on silica gel (hexanes/EtOAc = 8:1) to provide bipyridyl nonaflate **5e** (195 mg, 52%) as a colorless solid.

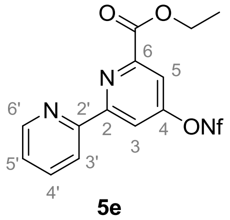

5e

M.p.: 64-65 °C.

^{1}**H NMR** ($CDCl_3$, 500 MHz): δ = 1.48, 4.52 (t, q, *J* = 7.2 Hz, 3 H, 2 H, OCH_2CH_3), 7.40 (ddd, *J* = 7.5, 4.8, 1.2 Hz, 1 H, 5'-H), 7.87 (td, *J* ≈ 7.8, 1.8 Hz, 1 H, 4'-H), 8.00 (d, *J* = 2.4 Hz, 1 H, 3-H), 8.58 (dt, *J* ≈ 8.0, 1.0 Hz, 1 H, 3'-H), 8.59 (d, *J* = 2.4 Hz, 1 H, 5-H), 8.70 (ddd, *J* = 4.8, 1.8, 0.9 Hz, 1 H, 6'-H) ppm.

^{13}**C NMR** ($CDCl_3$, 126 MHz): δ = 14.2 (q, CH_3), 62.5 (t, OCH_2), 116.1 (d, C-5), 117.2 (d, C-3), 121.9 (d, C-3'), 125.2 (d, C-5'), 137.2 (d, C-4'), 149.4 (d, C-6'), 150.7, 153.3 (2 s, C-2, C-2'), 158.1 (s, C-4), 159.9 (s, C-6), 163.7 (C=O) ppm.

^{19}**F NMR** ($CDCl_3$, 376 MHz): δ = –125.6, –120.6 (2 m_c, 2 F each, CF_2), –108.2 (t, *J* = 13.8 Hz, 2 F, CF_2), –80.4 (t, *J* = 9.9 Hz, 3 F, CF_3) ppm.

IR (ATR): ν = 3095, 3060 (=C-H), 2985, 2940 (C-H), 1750-1725 (C=O), 1590-1560 (C=C, C=N), 1435-1420, 1335, 1235-1145 cm^{-1}.

HRMS (ESI-TOF): $C_{17}H_{11}F_9N_2O_5S \cdot H^+$: calcd.: 527.0318; found: 527.0312.

EA: $C_{17}H_{11}F_9N_2O_5S$ (526.3) calcd. (%): C 38.79, H 2.11, N 5.32; found (%): C 38.71, H 2.09, N 5.28.

(4'-Chloro-6-methyl-2,2'-bipyridin-4-yl) nonaflate (5f):

According to **GP-1**, β-ketoenamide **3f** (1.20 g, 5.03 mmol) was treated with DIPEA (3.42 mL, 20.1 mmol) and TMSOTf (4.57 mL, 25.2 mmol) in DCE (125 mL) for 3 d under reflux, followed by nonaflation of the crude product using NaH (1.41 g, 35.3 mmol) and NfF (2.71 mL, 15.1 mmol) in THF (60 mL). After evaporation of THF under reduced pressure, the

remaining residue was dissolved in CH_2Cl_2 (50 mL) and washed with water (3 × 50 mL). The organic layer was dried with $NaSO_4$, filtered and evaporated to dryness under reduced pressure. The obtained crude product was purified by flash column chromatography on silica gel (hexanes/EtOAc = 50:1, 20:1) to provide bipyridyl nonaflate **5f** (1.67 g, 66%) as a colorless solid.

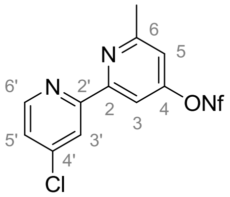

5f

M.p.: 38-39 °C.

^{1}H NMR ($CDCl_3$, 700 MHz): δ = 2.70 (s, 3 H, CH_3), 7.12 (d, *J* = 2.2 Hz, 1 H, 5-H), 7.35 (dd, *J* = 5.2, 2.1 Hz, 1 H, 5'-H), 8.19 (d, *J* = 2.2 Hz, 1 H, 3-H), 8.48 (dd, *J* = 2.1, 0.5 Hz, 1 H, 3'-H), 8.57 (dd, *J* = 5.2, 0.5 Hz, 1 H, 6'-H) ppm.

^{13}C NMR ($CDCl_3$, 176 MHz): δ = 24.8 (q, CH_3), 110.9 (d, C-3), 115.6 (d, C-5), 121.8 (d, C-3'), 124.7 (d, C-5'), 145.4 (s, C-4'), 150.2 (d, C-6'), 155.9, 157.6, 157.8 (3 s, C-2, C-2', C-4'), 161.5 (s, C-6) ppm.

^{19}F NMR ($CDCl_3$, 376 MHz): δ = –125.7, –120.7 (2 m_c, 2 F each, CF_2), –108.6 (t, *J* = 13.6 Hz, 2 F, CF_2), –80.5 (t, *J* = 9.4 Hz, 3 F, CF_3) ppm.

IR (ATR): ν = 3090 (=C-H), 2960, 2930 (C-H), 1600, 1570, 1550 (C=C, C=N), 1415, 1355, 1240-1140 cm^{-1}.

HRMS (ESI-TOF): $C_{15}H_8ClF_9N_2O_3S \cdot H^+$: calcd.: 502.9879; found: 502.9886.

EA: $C_{15}H_8ClF_9N_2O_3S$ (502.7) calcd. (%): C 35.84, H 1.60, N 5.57, S 6.38; found (%): C 35.95, H 1.65, N 5.58, S 6.39.

(6'-Methoxycarbonyl-6-methyl-2,2'-bipyridin-4-yl) nonaflate (5g):

According to **GP-1**, β-ketoenamide **3g** (226 mg, 1.00 mmol) was treated with DIPEA (0.68 mL, 4.00 mmol) and TMSOTf (0.91 mL, 5.00 mmol) in DCE (25 mL) for 3 d under reflux, followed by nonaflation of the crude product using NaH (200 mg, 5.00 mmol) and NfF (0.45 mL, 2.50 mmol) in THF (15 mL). After evaporation of THF under reduced pressure, the remaining residue was dissolved in CH_2Cl_2 (50 mL) and washed with water (3 × 50 mL). The organic layer was dried with $NaSO_4$, filtered and evaporated to dryness under reduced pressure. The obtained crude product was purified by flash column chromatography on silica

gel (hexanes/EtOAc = 15:1→8:1) to provide bipyridyl nonaflate **5g** (270 mg, 51 %) as a colorless solid.

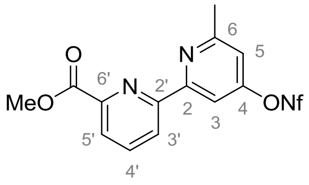

5g

M.p.: 103-105 °C.

^{1}H NMR ($CDCl_3$, 500 MHz): δ = 2.70 (s, 3 H, CH_3), 4.04 (s, 3 H, OCH_3), 7.14 (d, J = 2.2 Hz, 1 H, 5-H), 7.99 (t, J = 7.8 Hz, 1 H, 4'-H), 8.18 (dd, J = 7.8, 1.0 Hz, 1 H, 5'-H), 8.27 (d, J = 2.2 Hz, 1 H, 3-H), 8.63 (dd, J = 7.8, 1.0 Hz, 1 H, 3'-H) ppm.

^{13}C NMR ($CDCl_3$, 126 MHz): δ = 24. 8 (q, CH_3), 52.9 (q, OCH_3), 111.2 (d, C-3), 115.4 (d, C-5), 124.6 (d, C-3'), 125.7 (d, C-5'), 138.1 (d, C-4'), 147.8 (s, C-6'), 154.7 (s, C-2'), 157.8 (s, C-4), 158.0 (s, C-2), 161.4 (s, C-6), 165.6 (s, *C*$OOCH_3$) ppm.

^{19}F NMR ($CDCl_3$, 376 MHz): δ = –125.7 (m_c, 2 F, CF_2), –120.7 (m_c, 2 F, CF_2), –108.6 (t, J = 13.7 Hz, 2 F, CF_2), –80.5 (t, J = 9.7 Hz, 3 F, CF_3) ppm.

IR (ATR): ν = 3280, 3075 (=C-H), 2990–2945 (C-H), 1715 (C=O), 1600–1570, 1495, (C=C, C=N), 1425, 1200, 1125 cm^{-1}.

HRMS (ESI-TOF): $C_{17}H_{11}F_9N_2O_5S\cdot Na^+$: calcd.: 549.0137; found: 549.0132.

EA: $C_{17}H_{11}F_9N_2O_5S$ (526.3) calcd. (%): C 38.79, H 2.11, N 5.32, S 6.09; found (%): C 39.26, H 2.15, N 5.49, S 6.11.

1-[4-Methyl-2-(pyridin-2-yl)oxazol-5-yl]ethanone (6):

$BF_3\cdot OEt_2$ (0.14 mL, 1.11 mmol) was added slowly to a suspension of iodosylbenzene (119 mg, 0.54 mmol) in MeOH (3 mL) under an atmosphere of argon. β-Ketoenamide **3a** (100 mg, 0.49 mmol) was added and the resulting solution was stirred over night at rt The mixture was diluted with sat. aq. $NaHCO_3$ solution (40 mL) and extracted with CH_2Cl_2 (3 × 40 mL). The combined organic layers were dried with Na_2SO_4, filtered and evaporated under reduced pressure. The obtained crude product was purified by flash column chromatography on silica gel (hexanes/EtOAc = 3:1→2:1) to provide re-isolated starting material **3a** (9 mg, 9%) and oxazole **6** (55 mg, 55%) as a pale yellow solid.

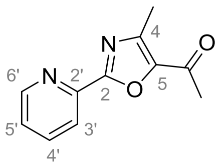

6

M.p.: 80-81 °C.

^{1}H NMR ($CDCl_3$, 500 MHz): δ = 2.57 (s, 3 H, 4-CH_3), 2.59 (s, 3 H, $COCH_3$), 7.42 (ddd, *J* = 7.6, 4.8, 1.2 Hz, 1 H, 5'-H), 7.84 (td, *J* ≈ 7.8, 1.7 Hz, 1 H, 4'-H), 8.15-8.17 (m, *J* ≈ 7.9, 1.0 Hz, 1 H, 3'-H), 8.77 (ddd, *J* = 4.8, 1.7, 0.9 Hz, 1 H, 6'-H) ppm.

^{13}C NMR ($CDCl_3$, 101 MHz): δ = 13.7 (q, 4-CH_3), 27.6 (q, CO*C*H_3), 123.0 (d, C-3'), 125.5 (d, C-5'), 137.0 (d, C-4'), 145.2, 145.7, 146.2 (3 s, C-2', C-4, C-5), 150.4 (d, C-6'), 159.7 (s, C-2), 187.9 (s, C=O) ppm.

IR (ATR): ν = 3070, 3010-3000 (=C-H), 2965, 2925, 2850 (C-H), 1670 (C=O), 1590, 1540 (C=C, C=N), 1460-1435, 1390-1245 cm^{-1}.

HRMS (ESI-TOF): $C_{11}H_{10}N_2O_2 \cdot H^+$: calcd.: 203.0815; found: 203.0814.

EA: $C_{11}H_{10}N_2O_2$ (202.2) calcd. (%): C 65.34, H 4.98, N 13.85; found (%): C 64.84, H 5.04, N 13.81.

5-Methoxy-4,6-dimethyl-2-(pyridin-2-yl)pyrimidine (7):

A mixture of β-ketoenamide **3i** (82 mg, 0.35 mmol) and NH_4OAc (432 mg, 2.80 mmol) in MeOH (1.4 mL) was stirred at 65 °C for 1 d in a sealed tube. TLC analysis indicated incomplete conversion of **3i**. Additional NH_4OAc (432 mg, 2.80 mmol) was added and the reaction mixture was stirred at 80 °C for another 2 d. The mixture was diluted with water (20 mL) and extracted with CH_2Cl_2 (3 × 20 mL). The combined organic layers were dried with Na_2SO_4, filtered and evaporated under reduced pressure. The obtained crude product was purified by flash column chromatography on silica gel (hexanes/EtOAc = 1:1; EtOAc/MeOH = 15:1) to provide pyrimidine **7** (57 mg, 76%) as a colorless solid.

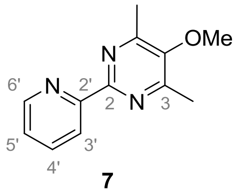

7

M.p.: 60-61 °C.

^{1}H NMR ($CDCl_3$, 500 MHz): δ = 2.58 (s, 6 H, CH_3), 3.79 (s, 3 H, OCH_3), 7.32 (ddd, *J* = 7.5, 4.8, 1.2 Hz, 1 H, 5'-H), 7.79 (td, *J* ≈ 7.8, 1.8 Hz, 1 H, 4'-H), 8.41-8.43 (m, *J* ≈ 8.0, 1.0 Hz, 1 H, 3'-H), 8.78 (ddd, *J* = 4.8, 1.8, 0.9 Hz, 1 H, 6'-H) ppm.

^{13}C NMR ($CDCl_3$, 126 MHz): δ = 19.0 (q, CH_3), 60.4 (q, OCH_3), 123.4 (d, C-3'), 124.1 (d, C-5'), 136.8 (d, C-4'), 149.9 (d, C-6'), 151.0, 154.9, 157.8 (3 s, C-2, C-2', C-4), 160.4 (s, C-3) ppm.

IR (ATR): ν = 3065, 3005 (=C-H), 2965, 2945, 2855 (C-H), 1560 (C=C, C=N), 1460, 1385, 1225 cm^{-1}.

HRMS (ESI-TOF): $C_{12}H_{13}N_3O·H^+$:calcd.: 216.1131; found: 216.1116.

EA: $C_{12}H_{13}N_3O$ (215.3) calcd. (%): C 66.96, H 6.09, N 19.52; found (%): C 66.89, H 6.06, N 19.42.

5-Methoxy-4,6-dimethyl-2-(pyridin-2-yl)pyrimidine 1-oxide (8):

A solution of β-ketoenamide **3i** (100 mg, 0.43 mmol) and $NH_2OH·HCl$ (74 mg, 1.07 mmol) in MeOH (1.4 mL) was stirred at rt for 1 d. TLC analysis indicated incomplete conversion of **3i**. Additional $NH_2OH×HCl$ (300 mg, 4.32 mmol) was added and the reaction mixture stirred for another 4 d. The mixture was diluted with water (30 mL) and extracted with CH_2Cl_2 (4 × 30 mL). The combined organic layers were dried with Na_2SO_4, filtered and evaporated under reduced pressure. The obtained crude product was purified by flash column chromatography on silica gel (hexanes/EtOAc = 1:1; EtOAc/MeOH = 4:1) to provide pyrimidine *N*-oxide **8** (64 mg, 65%) as a brown oil.

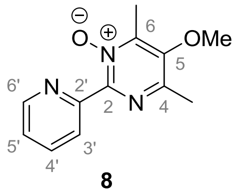

8

^{1}H NMR ($CDCl_3$, 500 MHz): δ = 2.55, 2.57 (2 s, 3 H each, 4-CH_3, 6-CH_3), 3.83 (s, 3 H, OCH_3), 7.36 (ddd, *J* = 7.6, 4.8, 1.1 Hz, 1 H, 5'-H), 7.81 (td, *J* ≈ 7.8, 1.8 Hz, 1 H, 4'-H), 8.40-8.42 (m, *J* ≈ 8.0, 1.0 Hz, 1 H, 3'-H), 8.81 (ddd, *J* = 4.8, 1.7, 0.8 Hz, 1 H, 6'-H) ppm.

^{13}C NMR ($CDCl_3$, 126 MHz): δ = 11.5 (q, 6-CH_3), 18.9 (q, 4-CH_3), 61.1 (q, OCH_3), 124.5 (d, C-5'), 125.6 (d, C-3'), 136.0 (d, C-4'), 148.5, 149.9, 150.1, 150.4,* 151.7 (s, d, 3 s, C-2, C-2', C-4, C-5, C-6, C-6') ppm; * signal corresponds to two carbon atoms.

IR (ATR): ν = 3055, 3000 (=C-H), 2930, 2855 (C-H), 1585-1565 (C=C, C=N), 1465, 1430, 1390, 1325, 1270, 1215-1125 cm^{-1}.

HRMS (ESI-TOF): $C_{12}H_{13}N_3O_2 \cdot H^+$: calcd.: 232.1081; found: 232.1070.

6-Methyl-4-*p*-tolyl-2,2'-bipyridine (9):

According to **GP-2**, bipyridyl nonaflate **5a** (200 mg, 0.427 mmol) was treated with 4-*p*-tolyl boronic acid (64 mg, 0.43 mmol), $Pd(PPh_3)_4$ (50 mg, 0.021 mmol) and K_2CO_3 (59 mg, 0.43 mmol) in DMF (2 mL) for 4 h. Workup was performed with EtOAc (25 mL) and water/brine (1:1, 3 × 25 mL). The obtained crude product was purified by flash column chromatography on silica gel (hexanes/EtOAc = 3:1) to provide bipyridine **9** (80 mg, 72%) as a colorless solid.

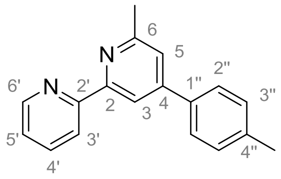

9

M.p.: 97-99 °C.

^{1}H NMR ($CDCl_3$, 500 MHz): δ = 2.42 (s, 3 H, 6-CH_3), 2.69 (s, 3 H, 4''-CH_3), 7.27-7.30 (m, 2 H, Ar), 7.31 (ddd, *J* = 1.0, 4.8, 7.8 Hz, 1 H, 5'-H), 7.40 (d, *J* = 1.6 Hz, 1 H, 5-H), 7.64-7.68 (m, 2 H, Ar), 7.82 (dt, *J* = 1.8, 7.8 Hz, 1 H, 4'-H), 8.44 (d, *J* = 1.6 Hz, 1 H, 3-H), 8.46 (td, *J* = 1.0, 7.8 Hz, 1 H, 3'-H), 8.70 (ddd, *J* = 1.0, 1.8, 4.8 Hz, 1 H, 6'-H) ppm.

^{13}C NMR ($CDCl_3$, 126 MHz): δ = 21.2 (q, 4''-CH_3), 24.7 (q, 6-CH_3), 116.1 (d, C-3), 120.9 (d, C-5), 121.3 (d, C-3'), 123.5 (d, C-5'), 126.5 (d, C-2''), 129.6 (d, C-3''), 135.6 (s, C-2), 136.8 (d, C-4'), 138.9 (s, C-4''), 149.1 (d, C-6'), 149.4 (s, C-1''), 156.1 (s, C-4), 156.6 (s, C-2), 158.4 (s, C-6) ppm.

IR (ATR): ν = 3060-3015 (=C-H), 2915, 2850 (C-H), 1600, 1580, 1545, 1515 (C=C, C=N), 1470-1410, 1385, 1260 cm^{-1}.

HRMS (ESI-TOF): $C_{18}H_{16}N_2 \cdot H^+$: calcd.: 261.1392; found: 261.1521.

EA: $C_{18}H_{16}N_2$ (260.3) calcd. (%): C 83.04, H 6.19, N 10.76; found (%): C 83.07, H 6.19, N 10.68.

1-[4-(6-Ethyl-3-methyl-2,2'-bipyridin-4-yl)phenyl]ethanone (10):

According to **GP-2**, bipyridyl nonaflate **5b** (520 mg, 1.05 mmol) was treated with 4-acetylphenylboronic acid (189 mg, 1.15 mmol), $Pd(PPh_3)_4$ (121 mg, 0.10 mmol) and K_2CO_3 (145 mg, 1.05 mmol) in DMF (5.5 mL) for 4 h. Workup was performed with EtOAc (25 mL) and water/brine (1:1, 3 × 25 mL). The obtained crude product was purified by flash column chromatography on silica gel (hexanes/EtOAc = 4:1→1:1) to provide bipyridine **10** (268 mg, 81%) as a colorless solid. For the elemental analysis, a sample of **10** was recrystallized from hexanes/EtOAc (2:1).

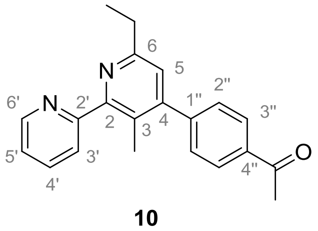

10

M.p.: 89-90 °C.

^{1}H NMR ($CDCl_3$, 500 MHz): δ = 1.34 (t, *J* = 7.6 Hz, 3 H, CH_2CH_3), 2.22 (s, 3 H, 3-CH_3), 2.65 (s, 3 H, $COCH_3$), 2.88 (q, *J* = 7.6 Hz, 2 H, CH_2CH_3), 7.06 (s, 1 H, 5-H), 7.30 (ddd, *J* = 7.5, 4.8, 1.2 Hz, 1 H, 5'-H), 7.47 (d, *J* = 8.1 Hz, 2 H, 2''-H), 7.73-7.74 (m, 1 H, 3'-H), 7.83 (td, *J* ≈ 7.7, 1.7 Hz, 1 H, 4'-H), 8.04 (d, *J* = 8.1 Hz, 2 H, 3''-H), 8.67-8.68 (m, 1 H, 6'-H) ppm.

^{13}C NMR ($CDCl_3$, 126 MHz): δ = 14.1 (q, CH_3CH_2), 16.9 (q, 3-CH_3), 26.6 (q, $COCH_3$), 31.0 (t, CH_2), 121.9 (d, C-5), 122.6 (d, C-5'), 124.4 (d, C-3'), 126.2 (s, C-3), 128.4 (d, C-3''), 129.1 (d, C-2''), 136.4 (s, C-4''), 136.7 (d, C-4'), 144.9 (s, C-4), 148.5 (d, C-6'), 150.4 (s, C-1''), 157.1, 159.5 (2 s, C-2, C-2'), 160.2 (s, C-6), 197.6 (s, C=O) ppm.

IR (ATR): ν = 3055, 3015 (=C-H), 2965, 2920 (C-H), 1670 (C=O), 1605, 1585, 1565, 1540 (C=C, C=N), 1470-1345, 1270 cm^{-1}.

HRMS (ESI-TOF): $C_{21}H_{20}N_2O \cdot H^+$: calcd.: 317.1648; found: 317.1625.

EA: $C_{21}H_{20}N_2O$ (316.4) calcd. (%): C 79.72, H 6.37, N 8.85; found (%): C 79.40, H 6.15, N 8.95.

4'-Chloro-6-methyl-4-phenyl-2,2'-bipyridine (11):

Method A: According to **GP-2**, bipyridyl nonaflate **5g** (502 mg, 1.00 mmol) was treated with phenylboronic acid (122 mg, 1.00 mmol), $Pd(PPh_3)_4$ (58 mg, 0.05 mmol) and K_2CO_3 (138 mg, 1.00 mmol) in DMF (5 mL) for 4 h. Workup was performed with EtOAc (50 mL) and water/brine (1:1, 3 × 50 mL). The obtained crude product was purified by flash column

chromatography on silica gel (hexanes/EtOAc = 20:1) to provide bipyridine **5g** (256 mg, 91%).

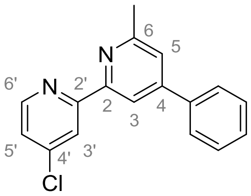

11

M.p.: 96-98 °C.

^{1}H NMR ($CDCl_3$, 500 MHz): δ = 2.70 (s, 3 H, CH_3), 7.31 (dd, *J* = 5.5, 2.1 Hz, 1 H, 5'-H), 7.42-7.45 (m, 2 H, 5-H, Ph), 7.47-7.50, 7.73-7.75 (2 m, 2 H each, Ph), 8.45 (d, *J* = 1.0 Hz, 1 H, 3-H), 8.52 (d, *J* = 2.1 Hz, 1 H, 3'-H), 8.57 (d, *J* = 5.5 Hz, 1 H, 6'-H) ppm.

^{13}C NMR ($CDCl_3$, 126 MHz): δ = 24.7 (q, CH_3), 116.6 (d, C-3), 121.67, 127.73 (2 d, C-3', C-5), 123.7 (d, C-5'), 127.1, 128.9*, 138.4 (2 d, s, Ph), 145.1 (s, C-4'), 149.7 (s, C-4), 149.9 (d, C-6'), 154.8 (s, C-2'), 158.0 (s, C-2), 158.6 (s, C-6) ppm; * the signal corresponds to two carbon atoms.

IR (ATR): ν = 3055 (=C-H), 2955, 2925, 2850 (C-H), 1605, 1570, 1550, 1500 (C=C, C=N), 1450, 1380, 1360 cm^{-1}.

HRMS (ESI-TOF): $C_{17}H_{13}ClN_2\cdot H^+$: calcd.: 281.0840; found: 281.0850.

EA: $C_{17}H_{13}ClN_2$ (280.8) calcd. (%): C 72.73, H 4.67, N 9.98; found (%): C 72.82, H 4.77, N 9.98.

4'-(4-Fluorophenyl)-6-methyl-4-phenyl-2,2'-bipyridine (12):

According to **GP-2**, 4'-chlorobipyridine **11** (253 mg, 0.90 mmol) was treated with 4-fluorophenylboronic acid (126 mg, 0.90 mmol), $Pd(PPh_3)_4$ (52 mg, 0.05 mmol) and K_2CO_3 (126 mg, 0.91 mmol) in DMF (3 mL) for 22 h. TLC analysis indicated incomplete conversion of **11**. Additional $Pd(PPh_3)_4$ (52 mg, 0.05 mmol), K_2CO_3 (63 mg, 0.46 mmol) and 4-fluorophenylboronic acid (63 mg, 0.45 mmol) were added and stirring at 80 °C was continued for another 24 h. Workup was performed with EtOAc (50 mL) and water/brine (1:1, 3 × 50 mL). The obtained crude product was purified by flash column chromatography on aluminum oxide (activity grade III, hexanes/EtOAc = 60:1→30:1) to provide recovered starting material **11** (77 mg, 30%), a mixed fraction (160 mg) containing **11** and bipyridine **12** and pure **12** (43 mg, 14%) as a colorless solid. The mixed fraction was further purified by three runs of preparative TLC (hexane/EtOAc = 4:1) to provide additional **11** (25 mg, 10%) and **12** (123 mg, 40%).

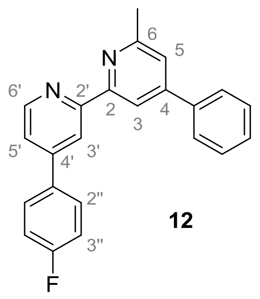

12

M.p.: 120-122 °C.

^{1}H NMR ($CDCl_3$, 500 MHz): δ = 2.72 (s, 3 H, CH_3), 7.20 (m_c, 2 H, 3''-H), 7.43-7.45 (m, 2 H, 5-H, 5'-H), 7.48-7.51 (m, 3 H, Ph), 7.73-7.78 (m, 4 H, Ph, 2''-H), 8.50 (d, J = 1.4 Hz, 1 H, 3-H), 8.68 (d, J = 3'-H), 8.73 (d, J = 5.1 Hz, 1 H, 6'-H) ppm.

^{13}C NMR ($CDCl_3$, 126 MHz): δ = 24.7 (q, CH_3), 116.0 (dd, $^2J_{CF}$ = 21.8 Hz, C-3''), 116.6 (d, C-3), 119.1 (d, C-3'), 121.28, 121.33 (2 d, C-5, C-5'), 127.1 (d, Ph), 128.86, 128.92 (2 d, Ph), 128.92 (dd, $^3J_{CF}$ = 8.4 Hz, C-2''), 134.5 (d, $^4J_{CF}$ = 3.1 Hz, C-1''), 138.5 (s, Ph), 148.2, 149.6 (2 s, C-4, C-4'), 149.6 (d, C-6'), 156.0, 157.0 (2 s, C-2, C-2'), 158.5 (s, C-6), 163.4 (d, $^1J_{CF}$ = 249.1 Hz, C-4'') ppm.

^{19}F NMR ($CDCl_3$, 471 MHz): δ = –112.6 (m_c) ppm.

IR (ATR): ν = 3090-3005 (=C-H), 2960-2860 (C-H), 1590, 1550, 1510 (C=C, C=N), 1470-1450, 1385, 1365, 1220, 1165 cm^{-1}.

HRMS (ESI-TOF): $C_{23}H_{17}FN_2{\cdot}Na^+$: calcd.: 363.1268; found: 363.1271.

4'-(Cyclopropylethynyl)-6-methyl-4-phenyl-2,2'-bipyridine (13):

A mixture of DMF and NEt_3 (1 mL, 1:1) was purged with argon for 30 min. The solvent mixture was then transferred to a sealed tube that was charged with chlorobipyridine **11** (88 mg, 0.31 mmol), $PdCl_2(PPh_3)_2$ (11.0 mg, 0.016 mmol), CuI (3.0 mg, 0.016 mmol) and cyclopropylethyne (414 mg, 6.26 mmol) under an atmosphere of argon. The mixture was stirred at 90 °C for 2 d. After cooling to rt, the mixture was diluted with EtOAc (30 mL) and washed with a mixture of water and brine (1:1, 3 × 30 mL). The organic layer was dried with Na_2SO_4, filtered and evaporated under reduced pressure. The obtained crude product was purified by flash column chromatography on aluminum oxide (activity grade III, hexanes/CH_2Cl_2 = 10:1, 3:1) to provide recovered starting material **11** (46 mg, 52%) and bipyridine **13** (47 mg, 48%) as a yellow oil that solidified upon storage.

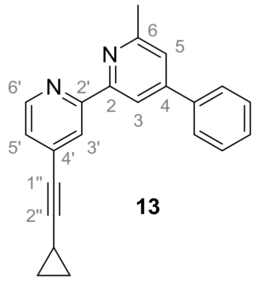

13

M.p.: 91-92 °C.

^{1}H NMR ($CDCl_3$, 500 MHz): δ = 0.85-0.89, 0.93 (m, m_c, 2 H each, CH_2), 1.50 (tt, *J* = 8.2, 5.1 Hz, 1 H, 2''-CH), 2.70 (s, 3 H, CH_3), 7.24 (dd, *J* = 5.1, 1.4 Hz, 1 H, 5'-H), 7.40-7.44 (m, 2 H, Ph, 5-H), 7.46-7.49, 7.73-7.75 (2 m, 2 H each, Ph), 8.41-8.42 (m, 2 H, 3-H, 3'-H), 8.59 (d, *J* = 5.1 Hz, 1 H, 6'-H) ppm.

^{13}C NMR ($CDCl_3$, 101 MHz): δ = 0.3 (d, 2''-CH), 8.9 (t, CH_2), 24.7 (q, CH_3), 74.1 (s, C-1''), 99.0 (s, C-2''), 116.4 (d, C-3), 121.3 (d, C-5), 123.5 (d, C-3'), 125.4 (d, C-5'), 127.1, 128.8, 128.9 (3 d, Ph), 133.1 (s, C-4'), 138.5 (s, Ph), 148.9 (d, C-6'), 149.5 (s, C-4), 155.6, 156.3 (2 s, C-2, C-2'), 158.4 (s, C-6) ppm.

IR (ATR): ν = 3085, 3060, 3010 (=C-H), 2960, 2920, 2855 (C-H), 2230 (C≡C), 1585, 1545, 1535 (C=C, C=N), 1455, 1385 cm^{-1}.

HRMS (ESI-TOF): $C_{22}H_{18}N_2 \cdot Na^+$: calcd.: 333.1362; found: 333.1384.

3) References

[1] Jiang, Y.; Wu, N.; Wu, H.; He, M. *Synlett* **2005**, 2731–2734.

[2] González, A.; Marquet, J.; Moreno-Mañas, M. *Tetrahedron Lett.* **1988**, *29*, 1469–1470.

[3] Gao, Y.; Zhang, Q.; Xu, J. *Synth. Commun.* **2004**, *34*, 909–916.

[4] Li, X.; Du, Y.; Liang, Z.; Li, X.; Pan, Y.; Zhao, K. *Org. Lett.* **2009**, *11*, 2643–2646.

[5] Baraldi, P. G.; Barco, A.; Benetti, S.; Manfredini, S.; Pollini, G. P.; Simoni, D. *Tetrahedron* **1987**, *43*, 235–242.

[6] Katritzky, A. R.; He, H.-Y.; Suzuki, K. *J. Org. Chem.* **2000**, *65*, 8210–8213.

[7] a) Saltzman, H.; Sharefkin, J. G.; Newman, M. S.; Gill, N. *Org. Synth.* **1963**, *43*, 60. b) *Org. Synth.* **1973**, *Coll. Vol. 5*, 658; c) Dauban, P.; Sanière, L.; Tarrade, A.; Dodd, R. H. *J. Am. Chem. Soc.* **2001**, *123*, 7707–7708.

[8] Johansen, J. E.; Christie, B. D.; Rapoport, H. *J. Org. Chem.* **1981**, *46*, 4914–4920.

[9] Dash, J.; Reissig, H.-U. *Chem. Eur. J.* **2009**, *15*, 6811–6814.

[10] Hommes, P.; Berlin, S.; Reissig, H.-U. *Synthesis* **2013**, *45*, 3288–3294..

[11] Hayes, J. F.; Shipman, M.; Twin, H. *J. Org. Chem.* **2002**, *67*, 935–942.

4) Copies of NMR spectra

^{1}H and ^{13}C NMR of **2d**

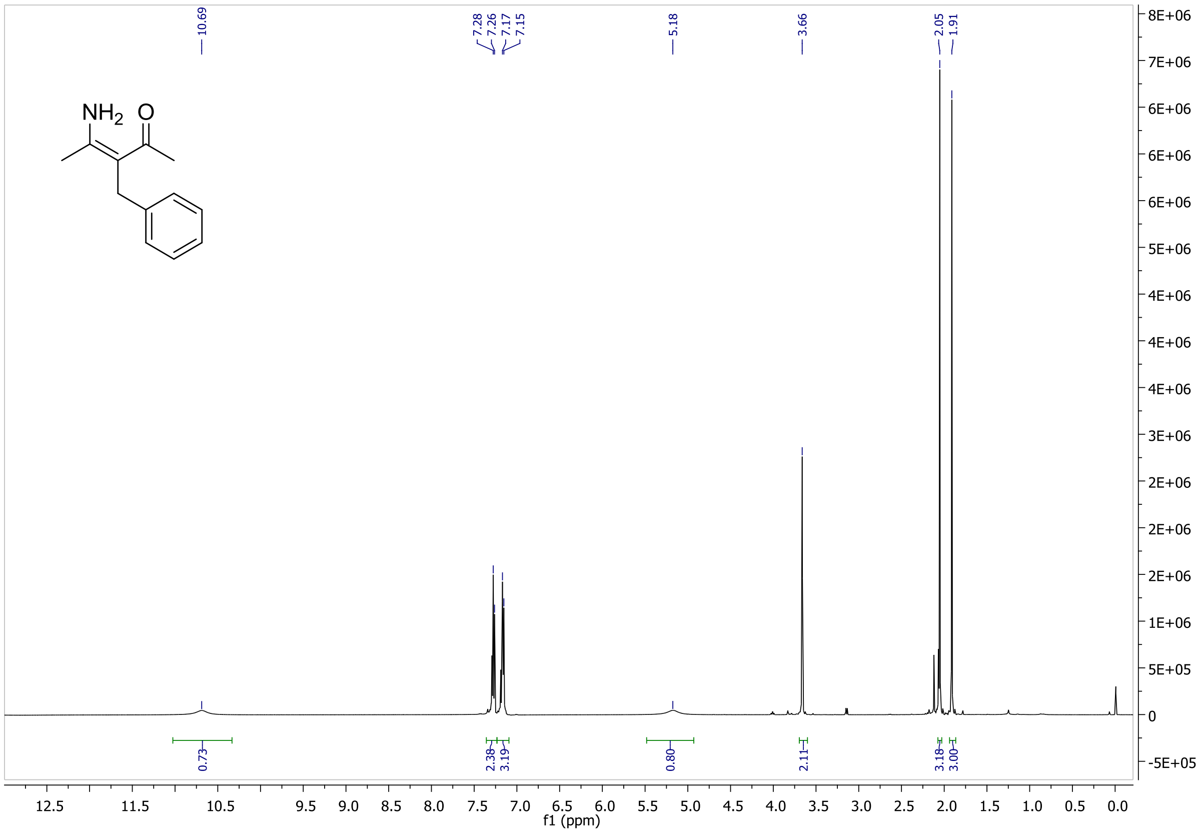

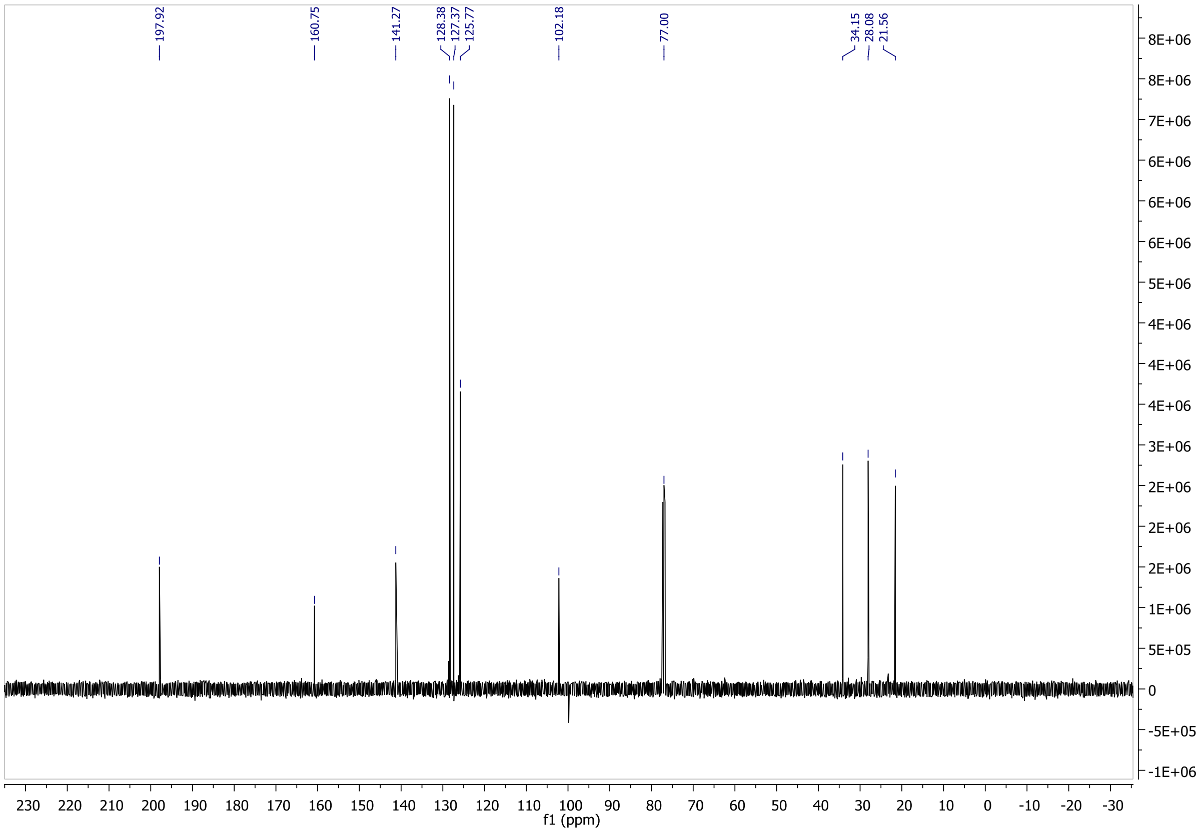

^{1}H and ^{13}C NMR of **2i**

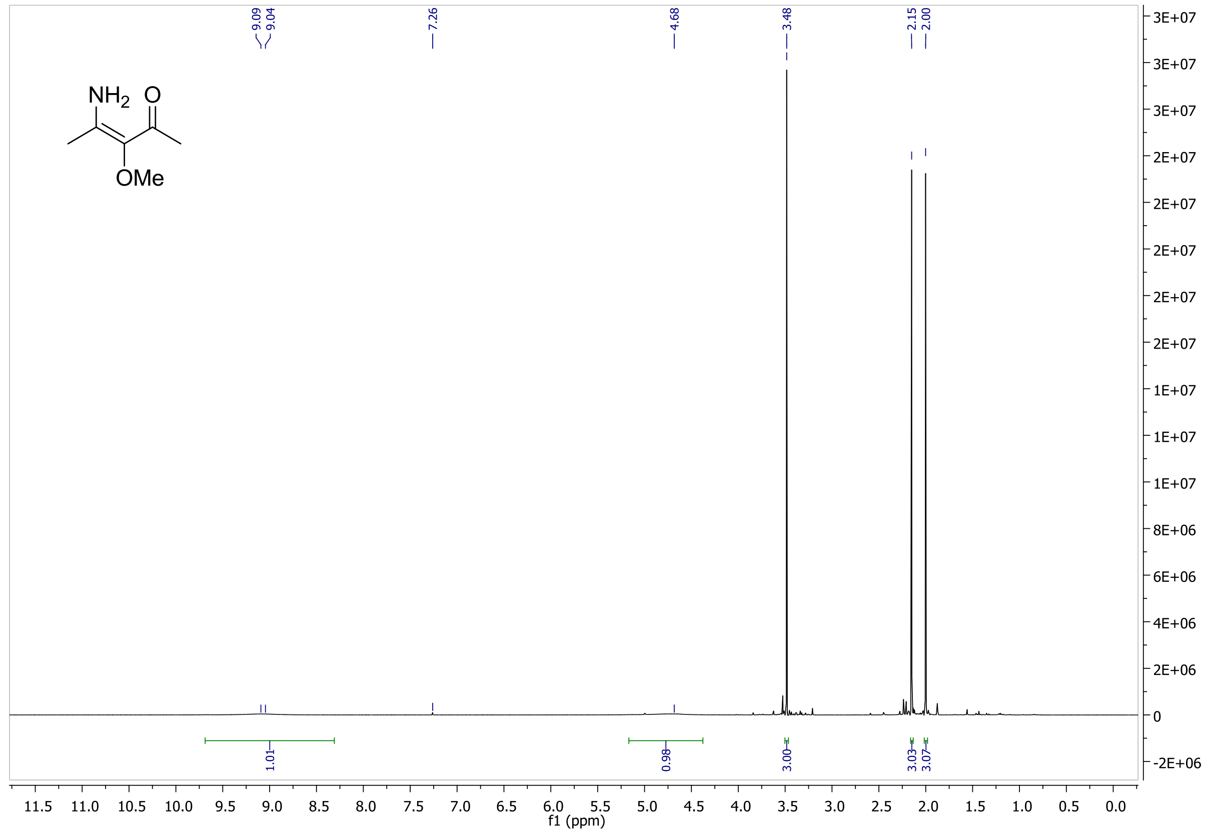

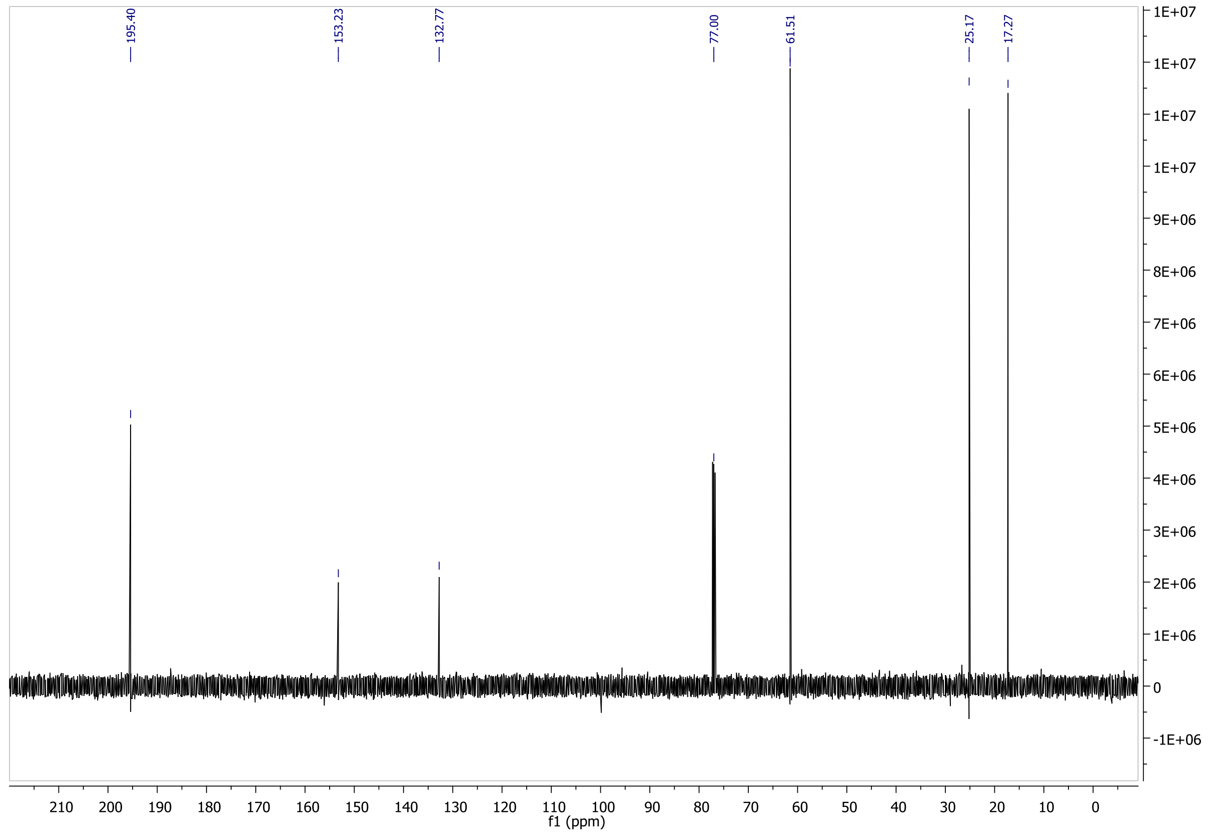

^{1}H and ^{13}C NMR of **3c**

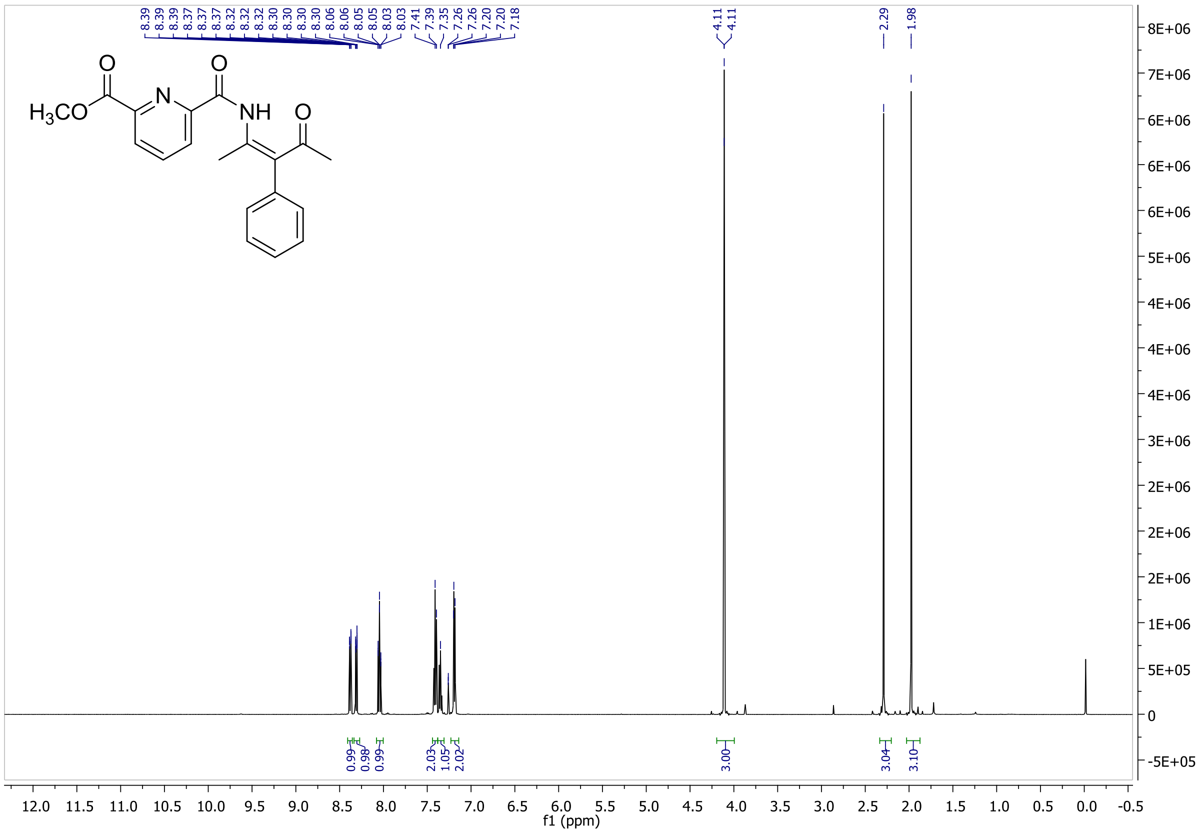

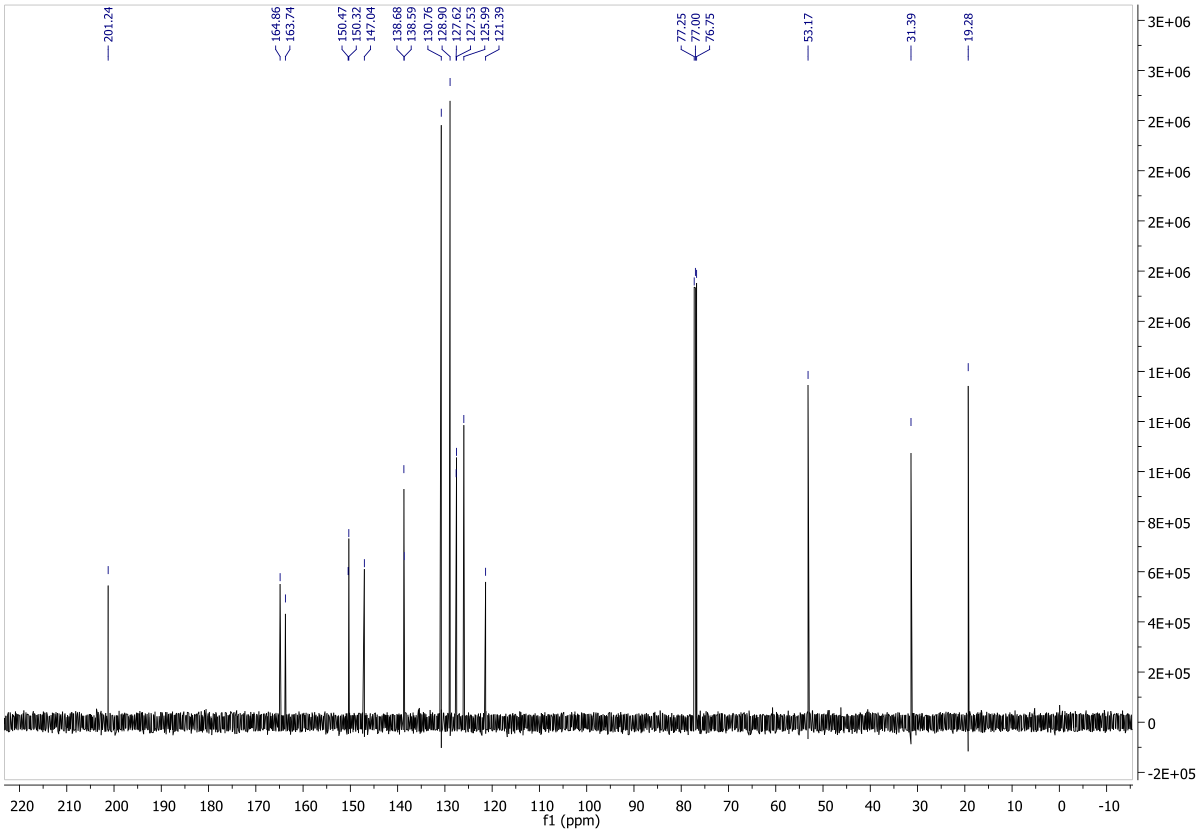

^{1}H and ^{13}C NMR of **3d**

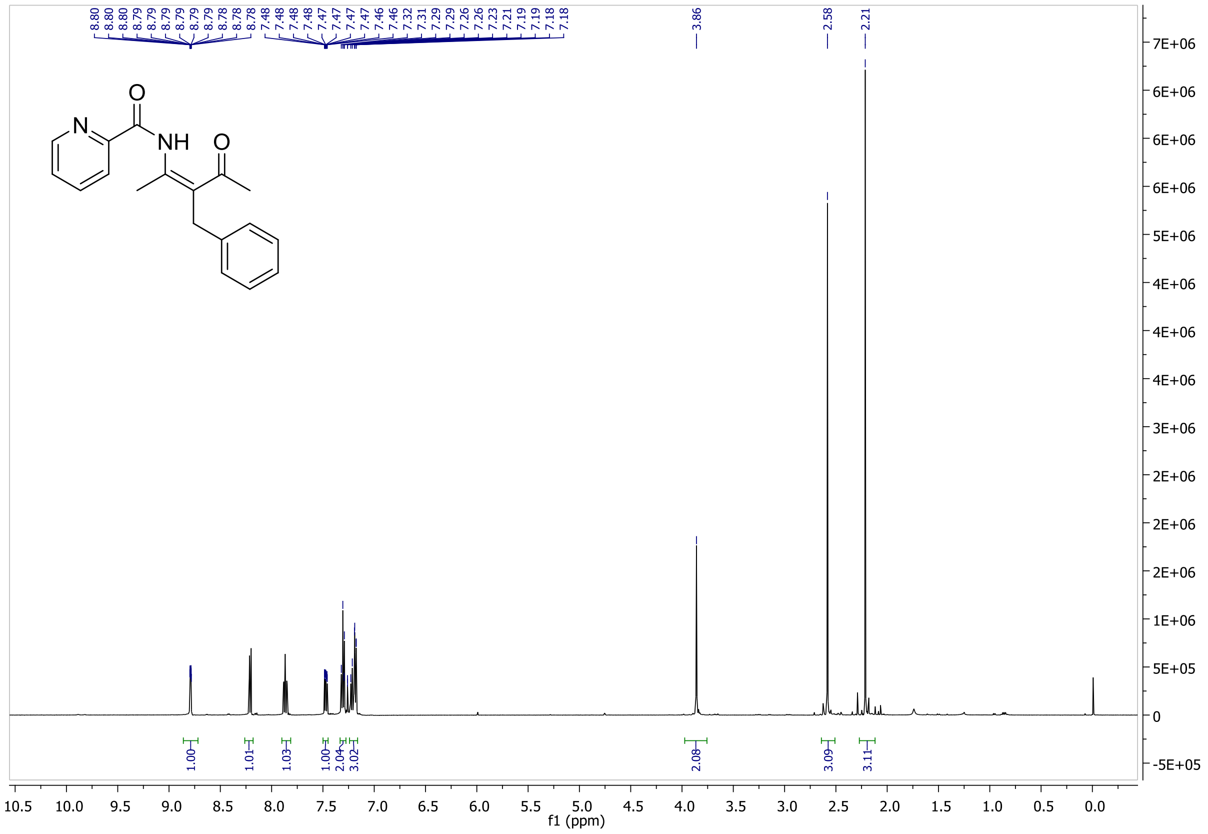

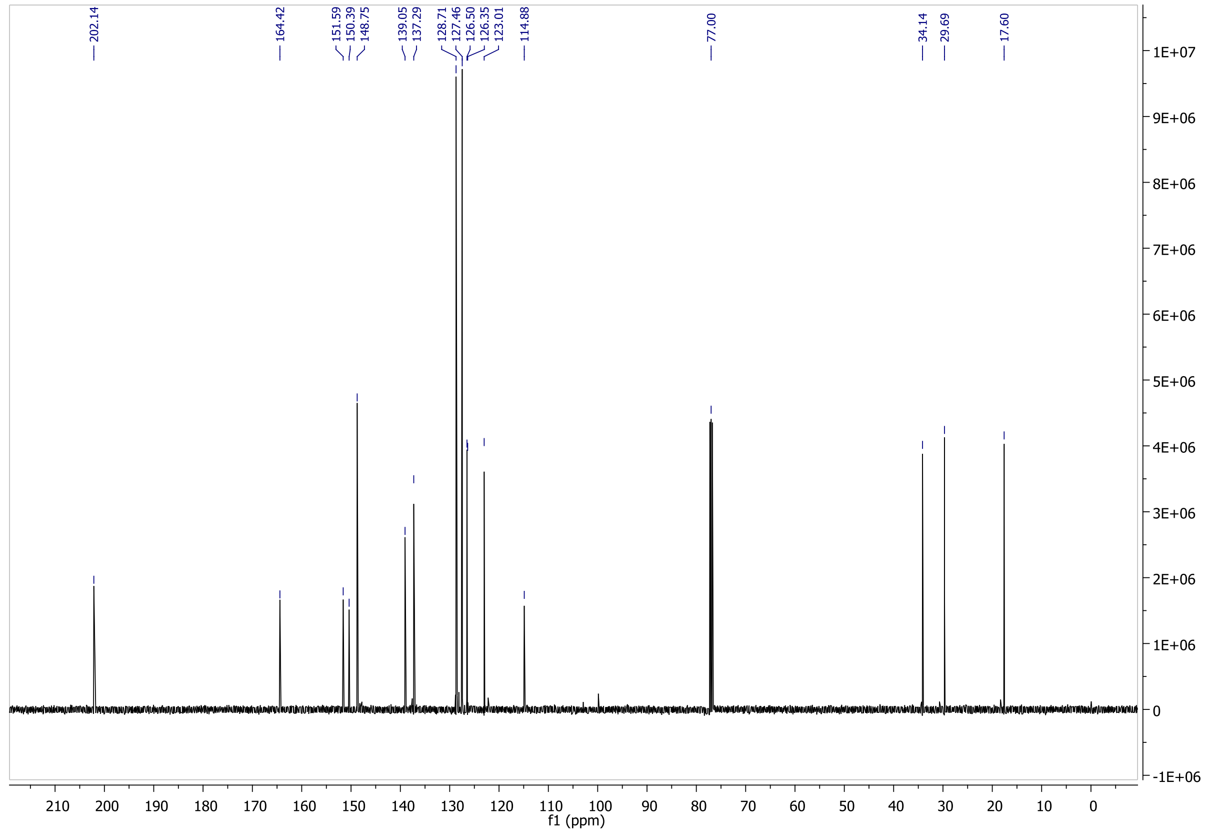

^{1}H and ^{13}C NMR of **3e**

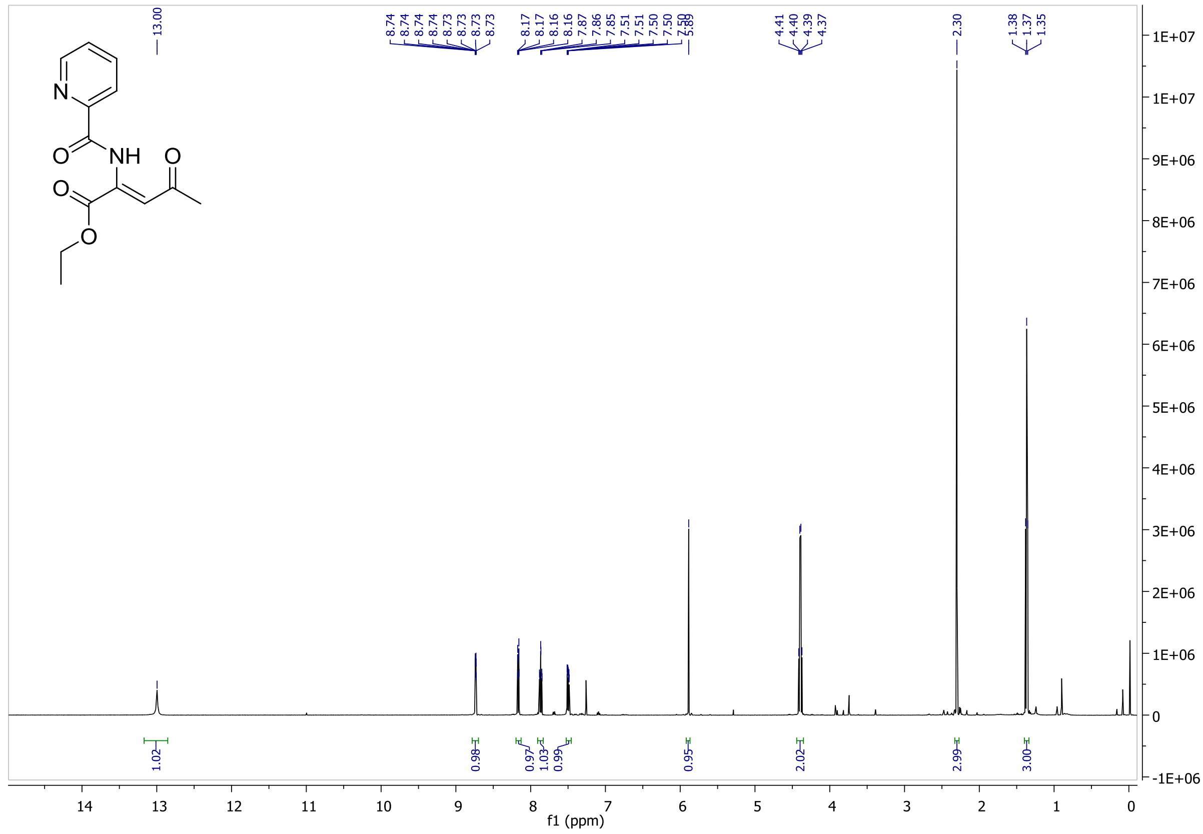

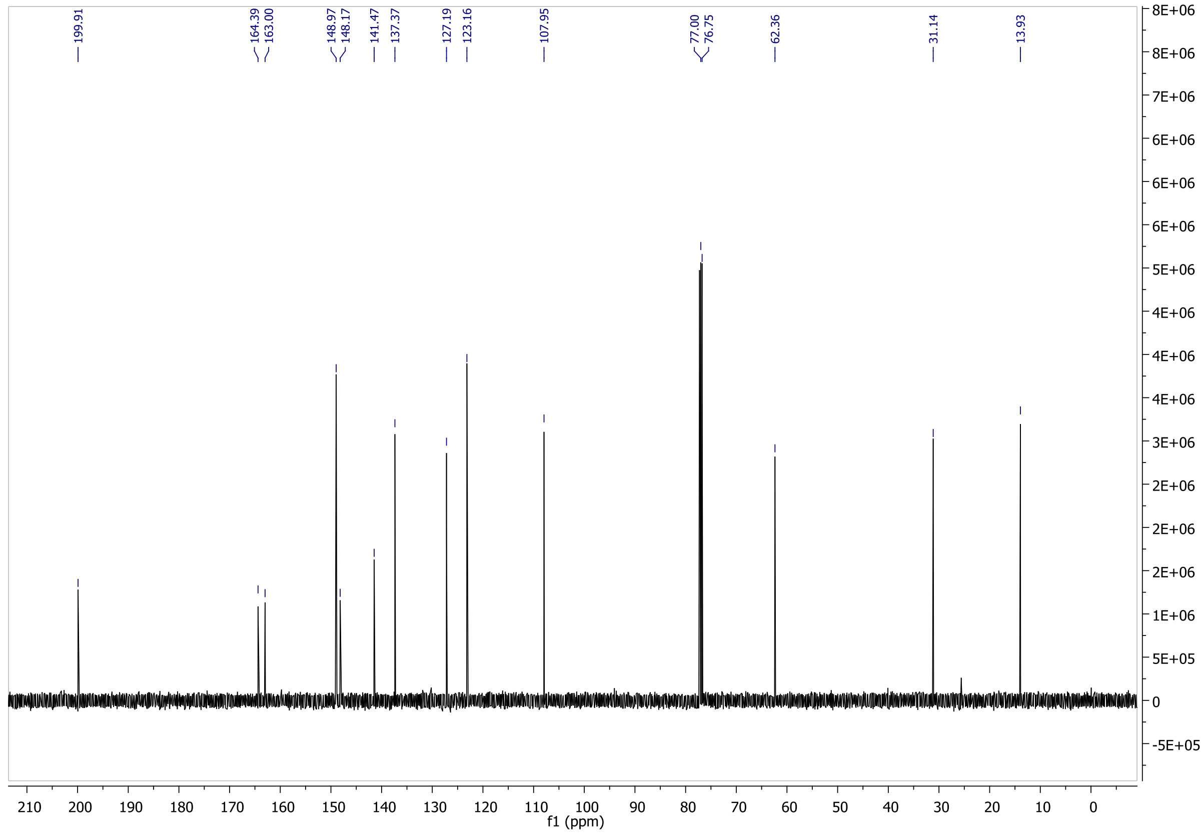

^{1}H and ^{13}C NMR of **3f**

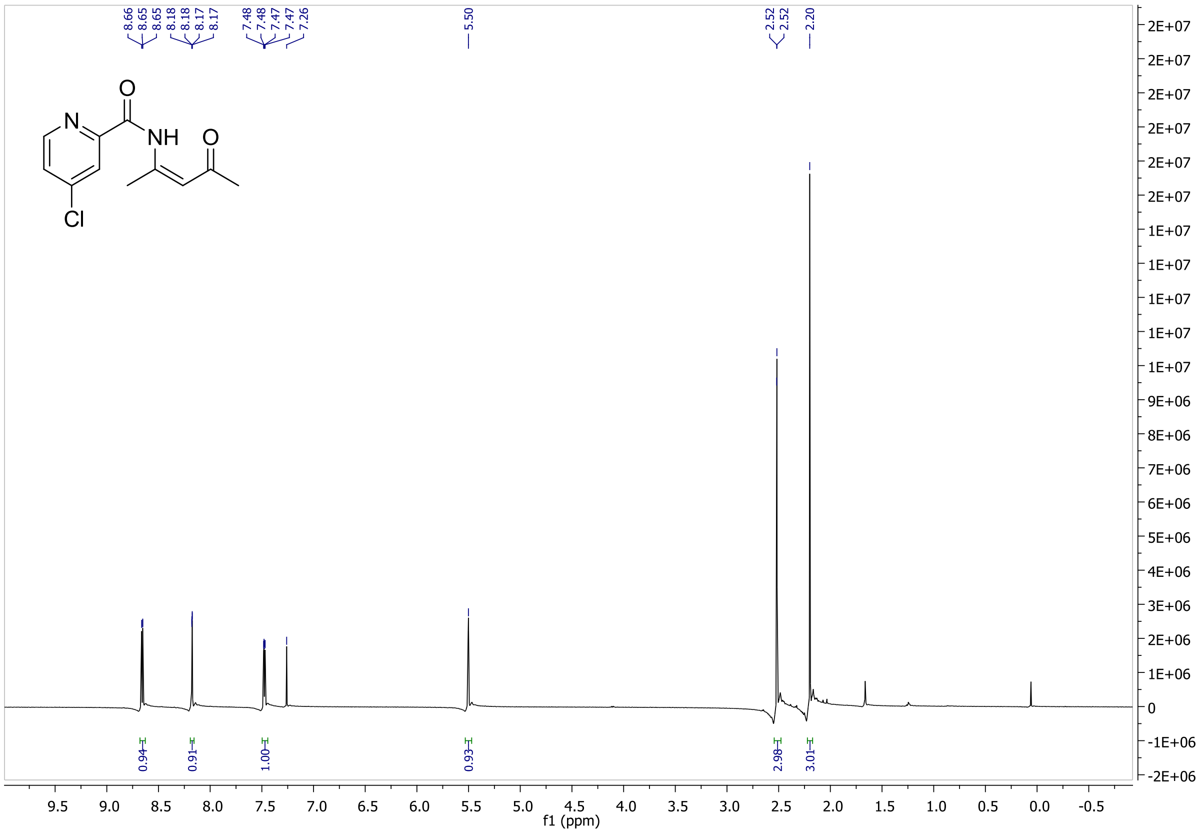

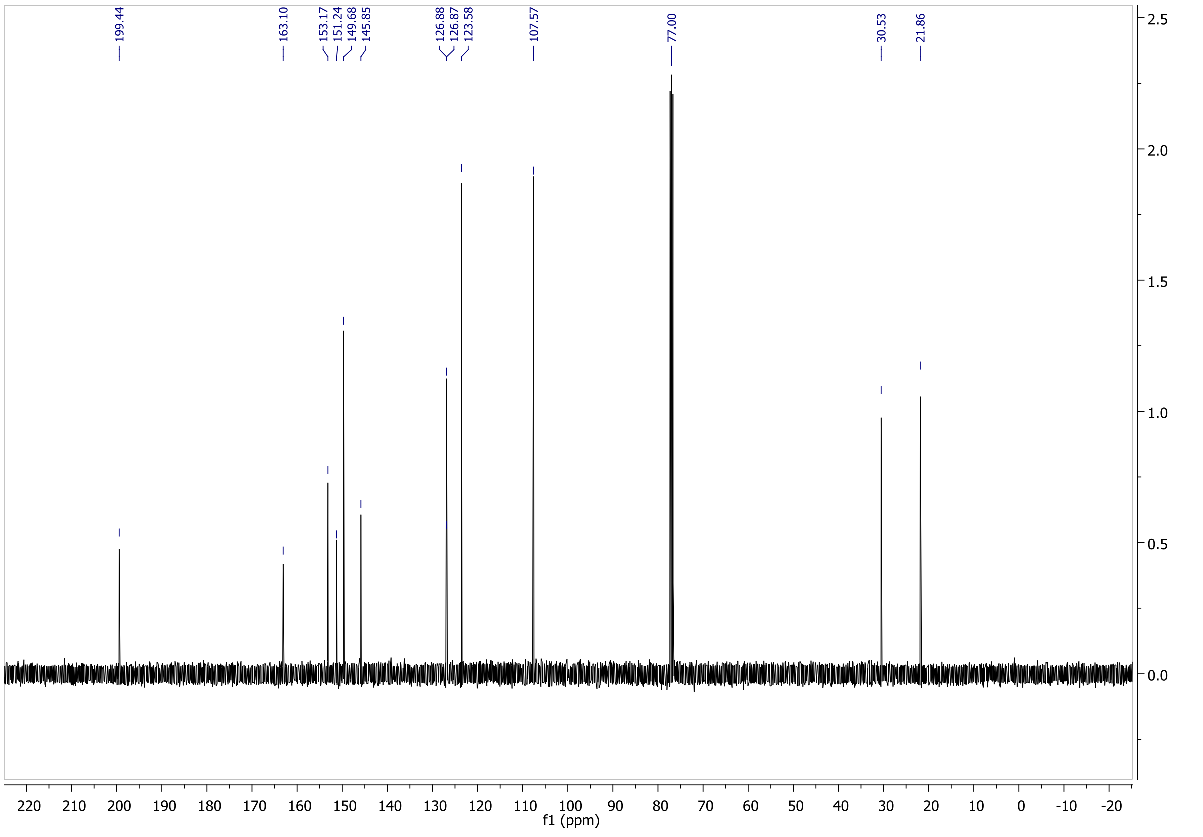

^{1}H and ^{13}C NMR of **3g**

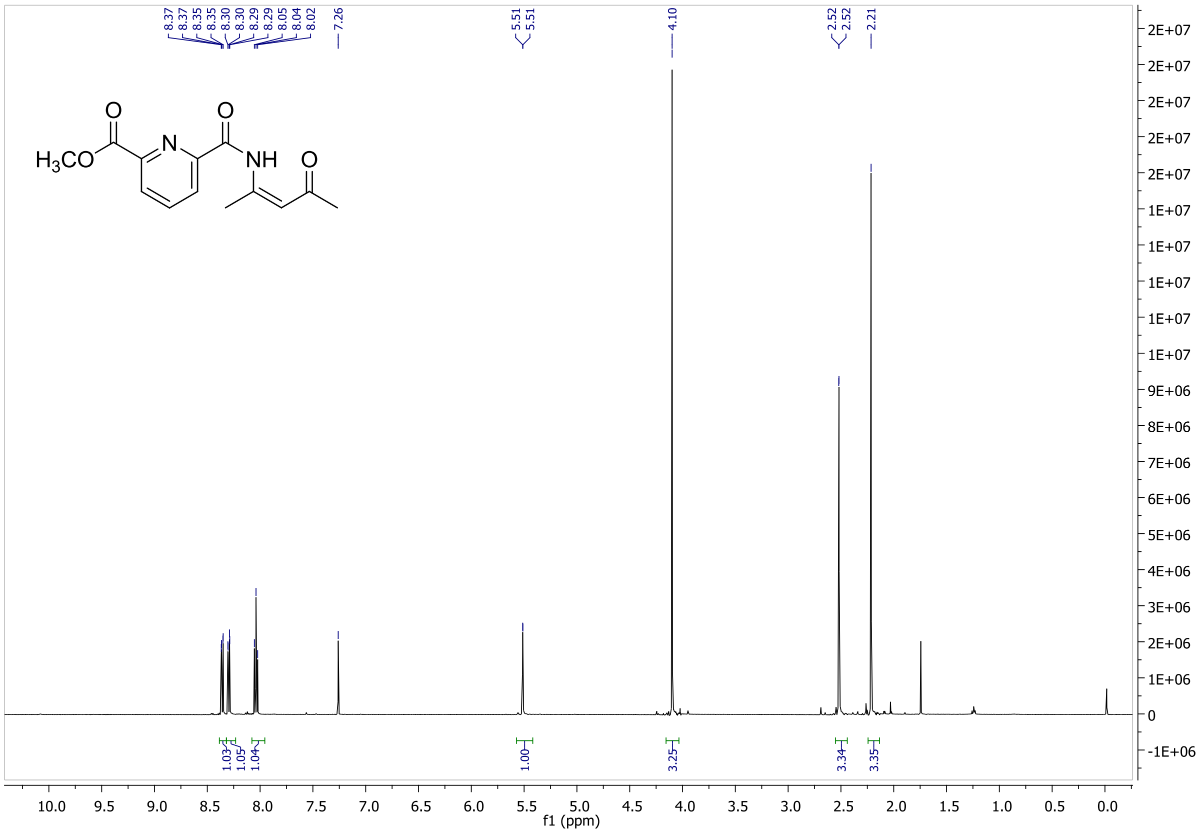

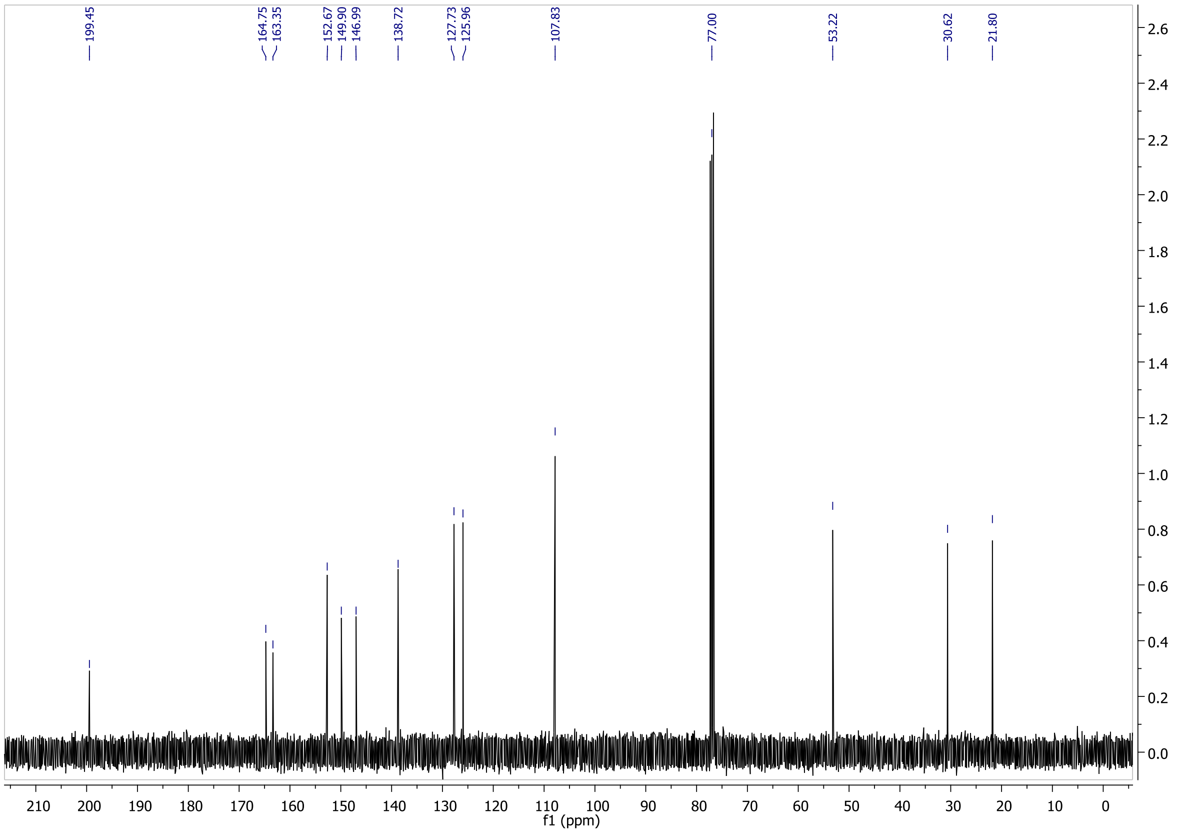

^{1}H and ^{13}C NMR of **3h**

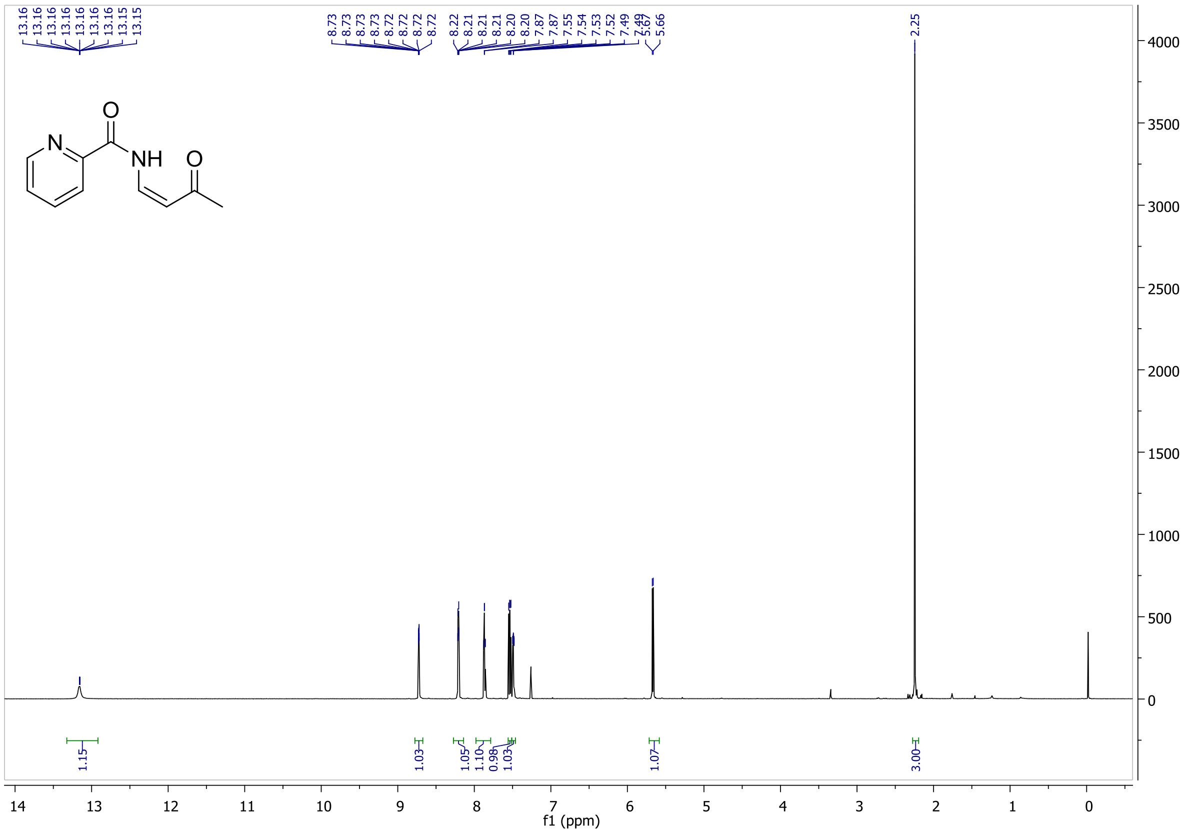

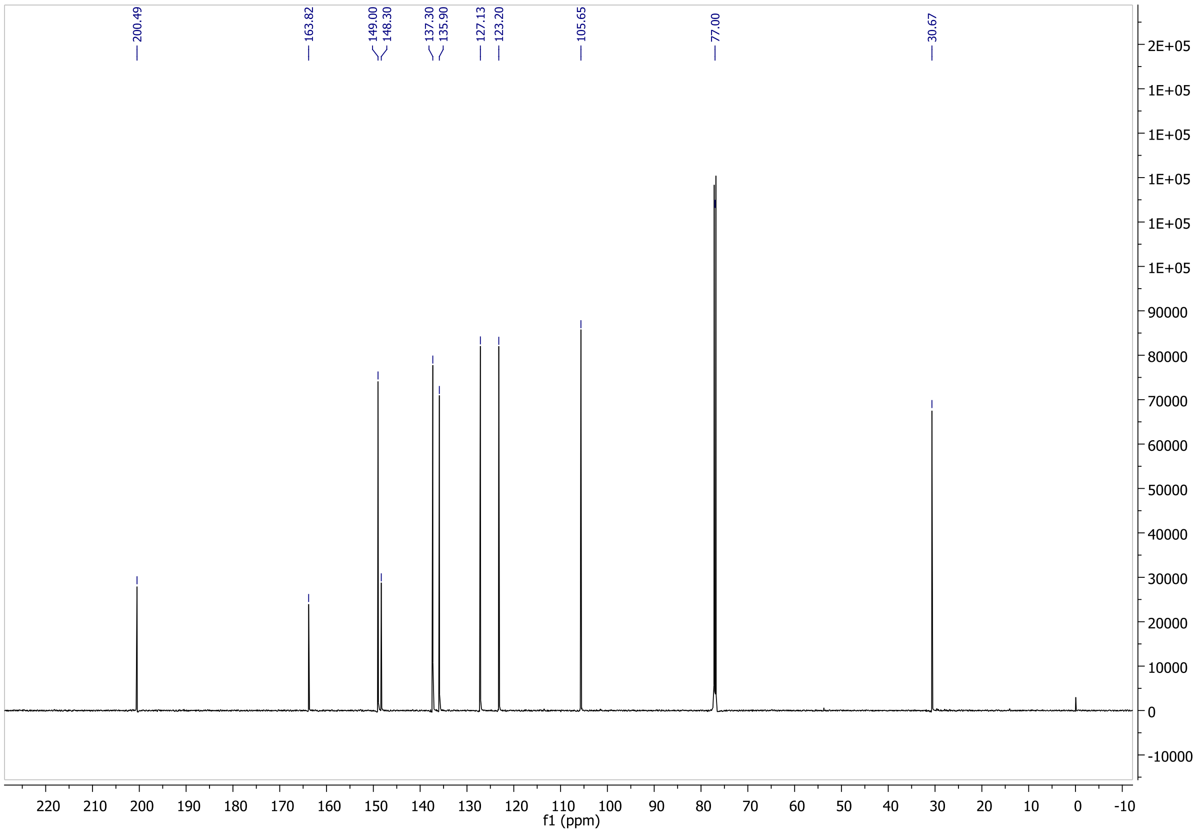

^{1}H and ^{13}C NMR of **3i**

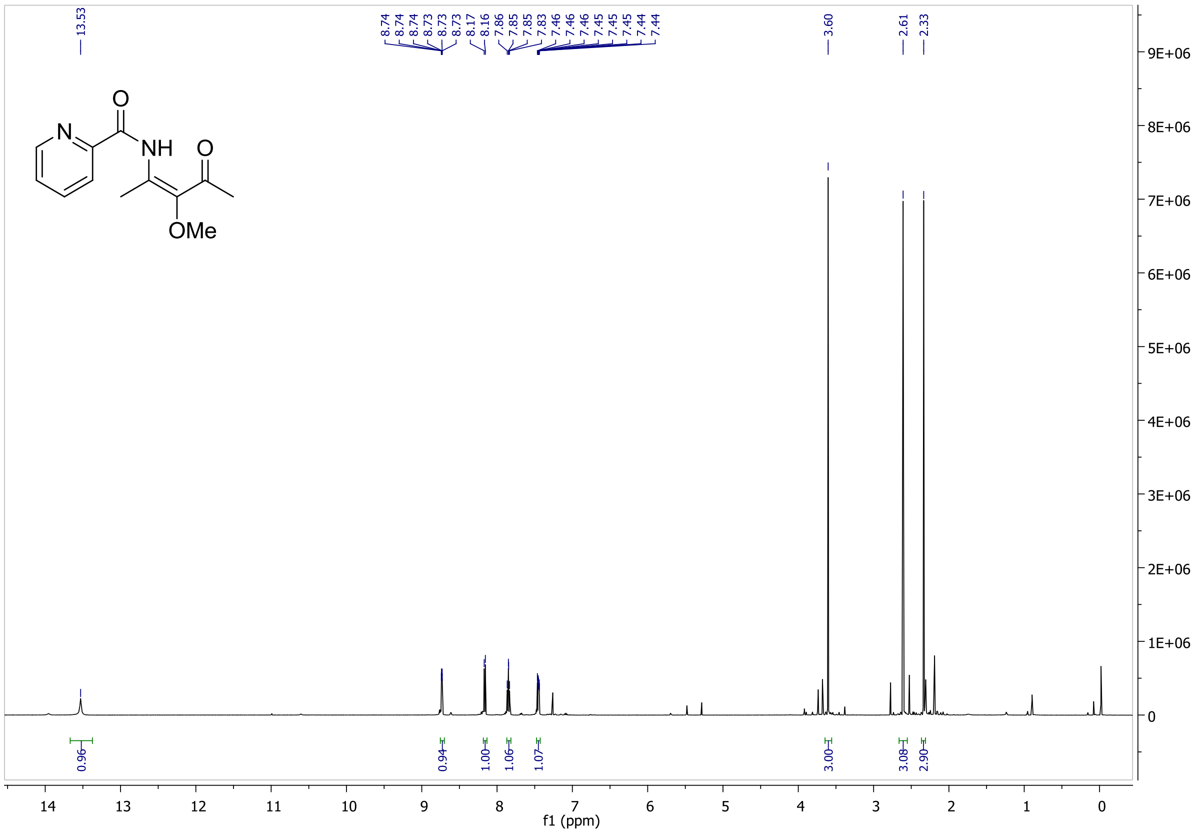

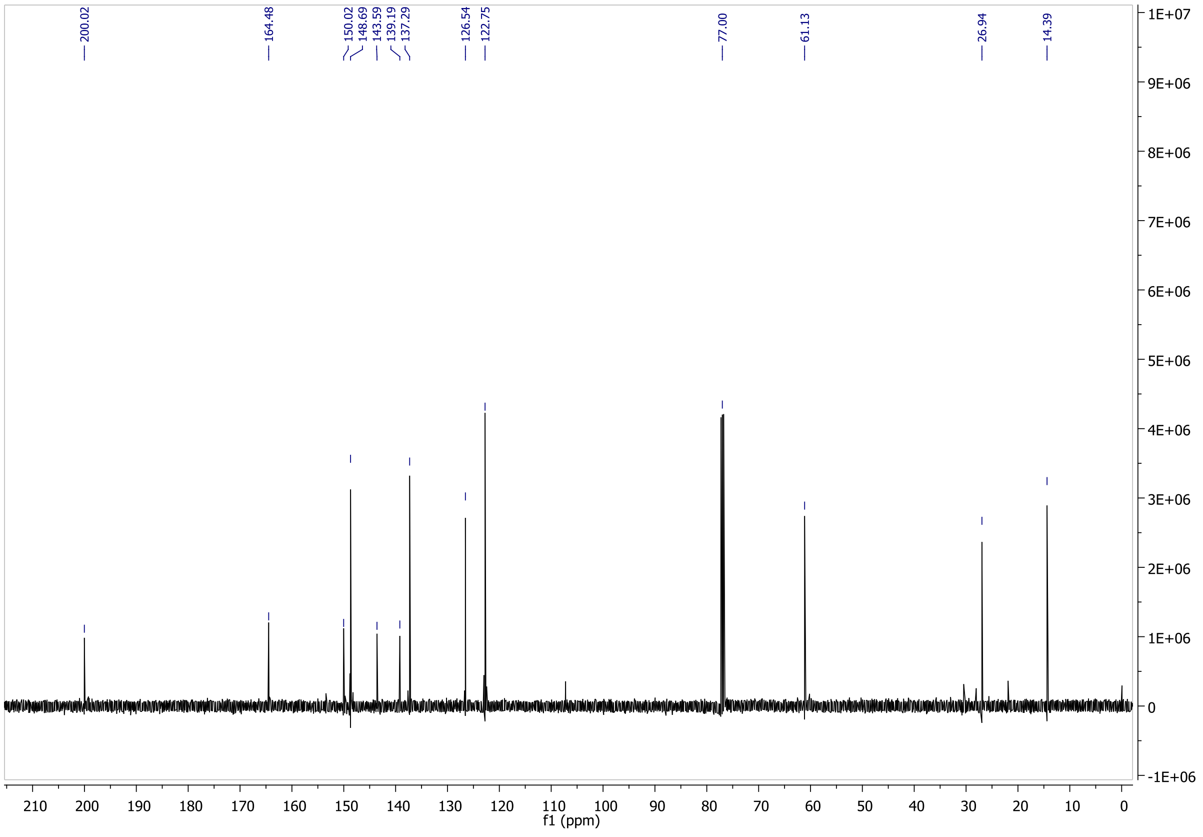

[1]H of **5a**

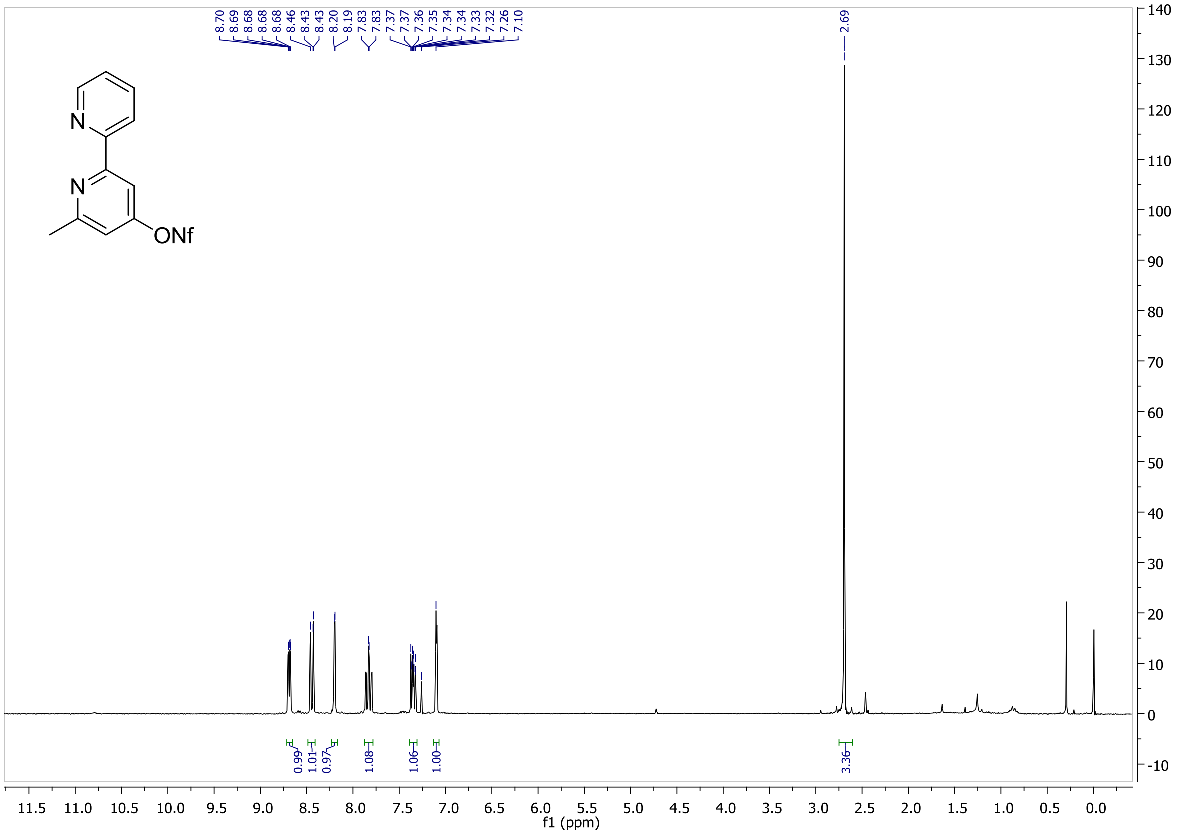

^{1}H and ^{13}C NMR of **5c**

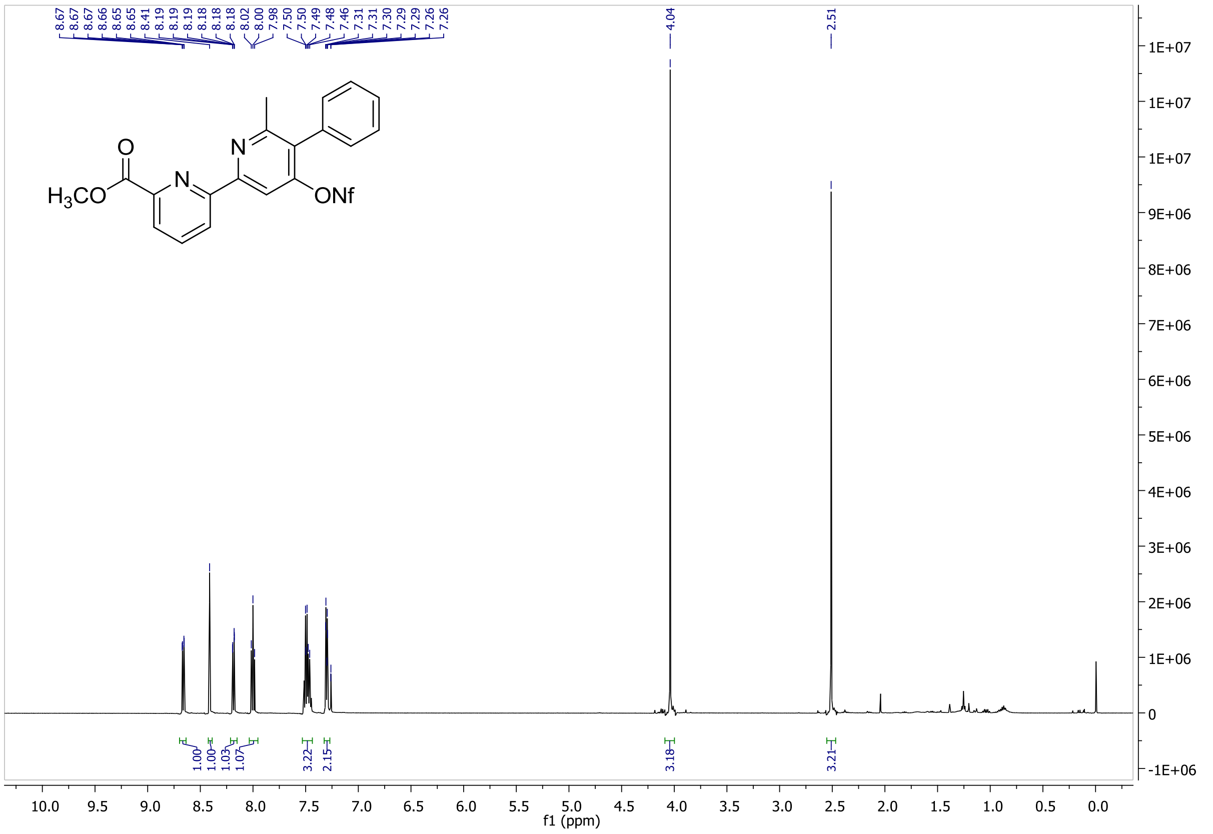

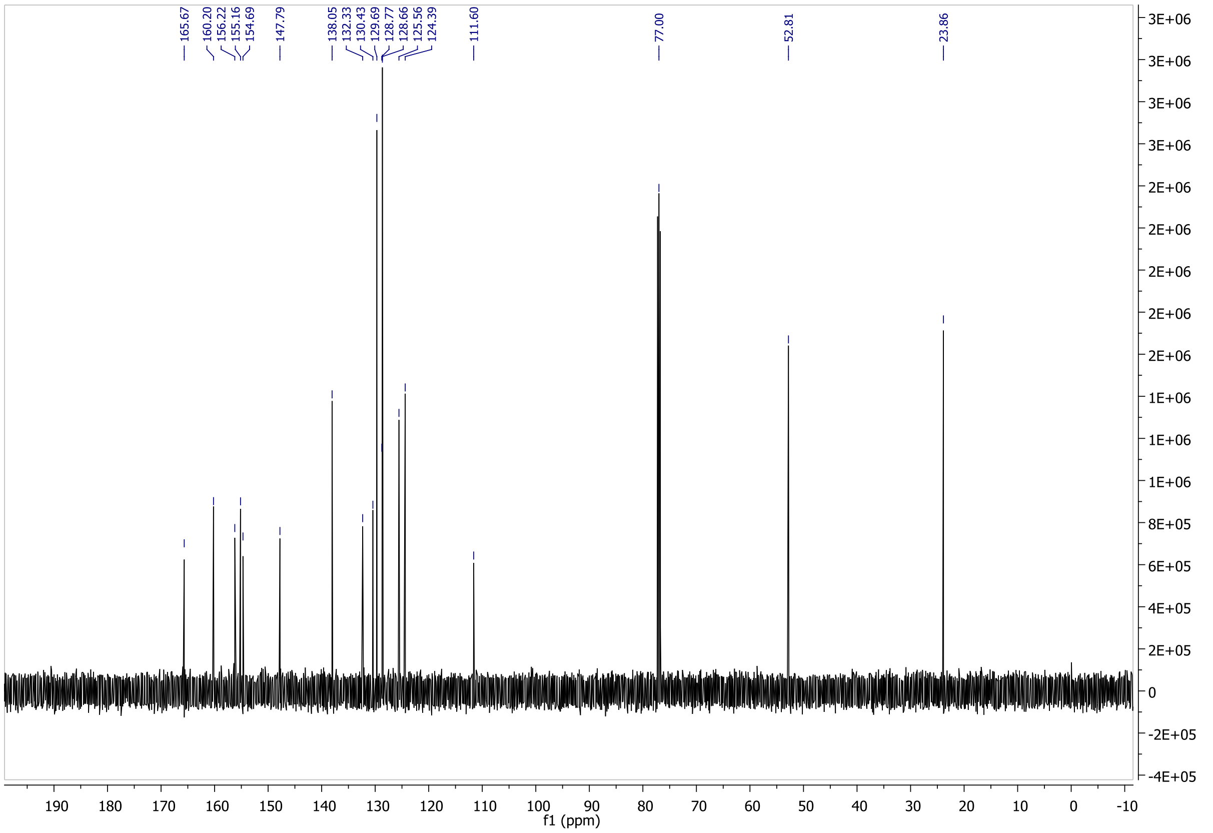

^{1}H and ^{13}C NMR of **5d**

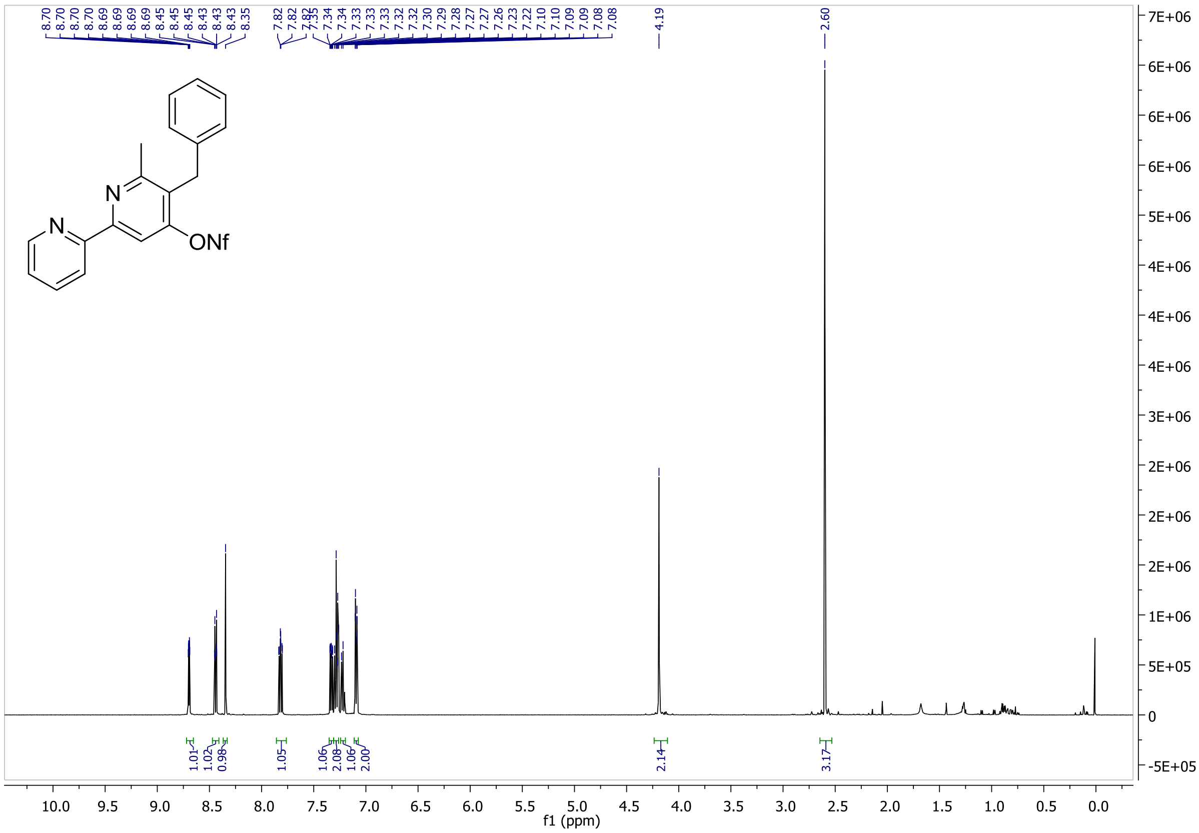

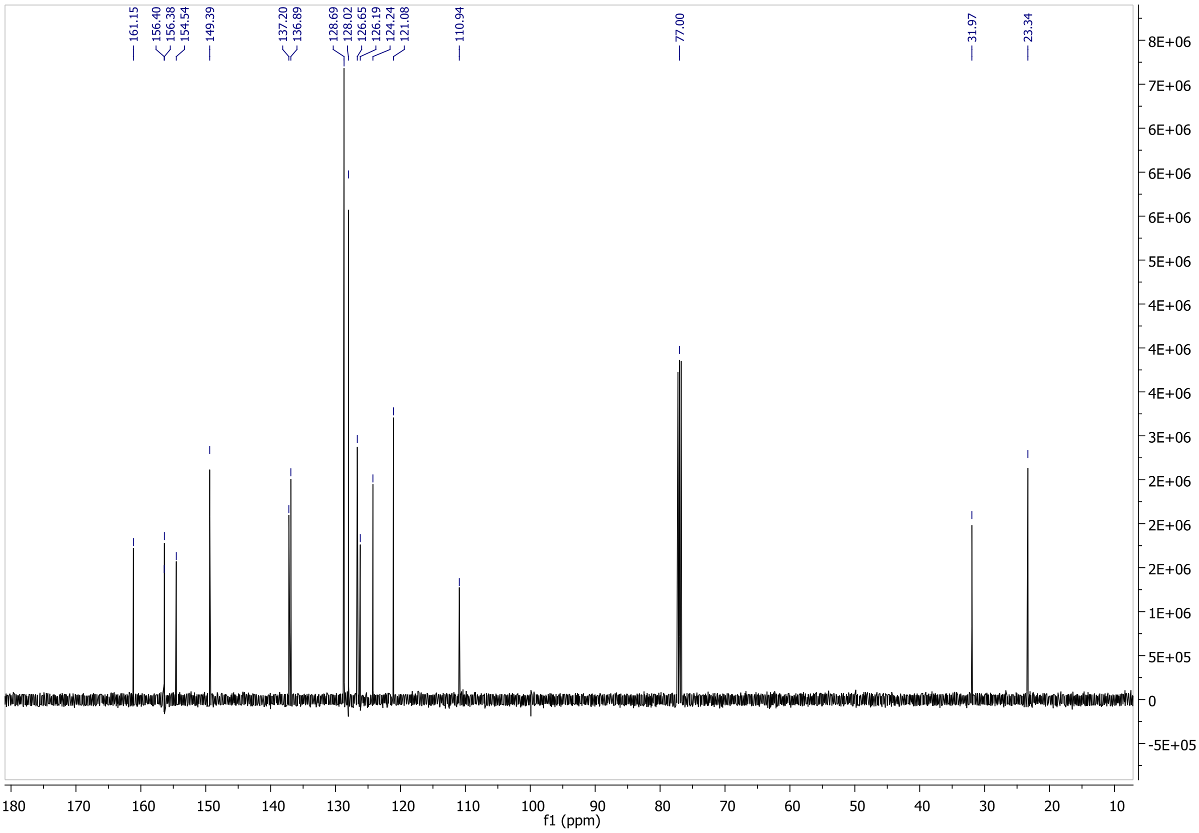

^{1}H and ^{13}C NMR of **5e**

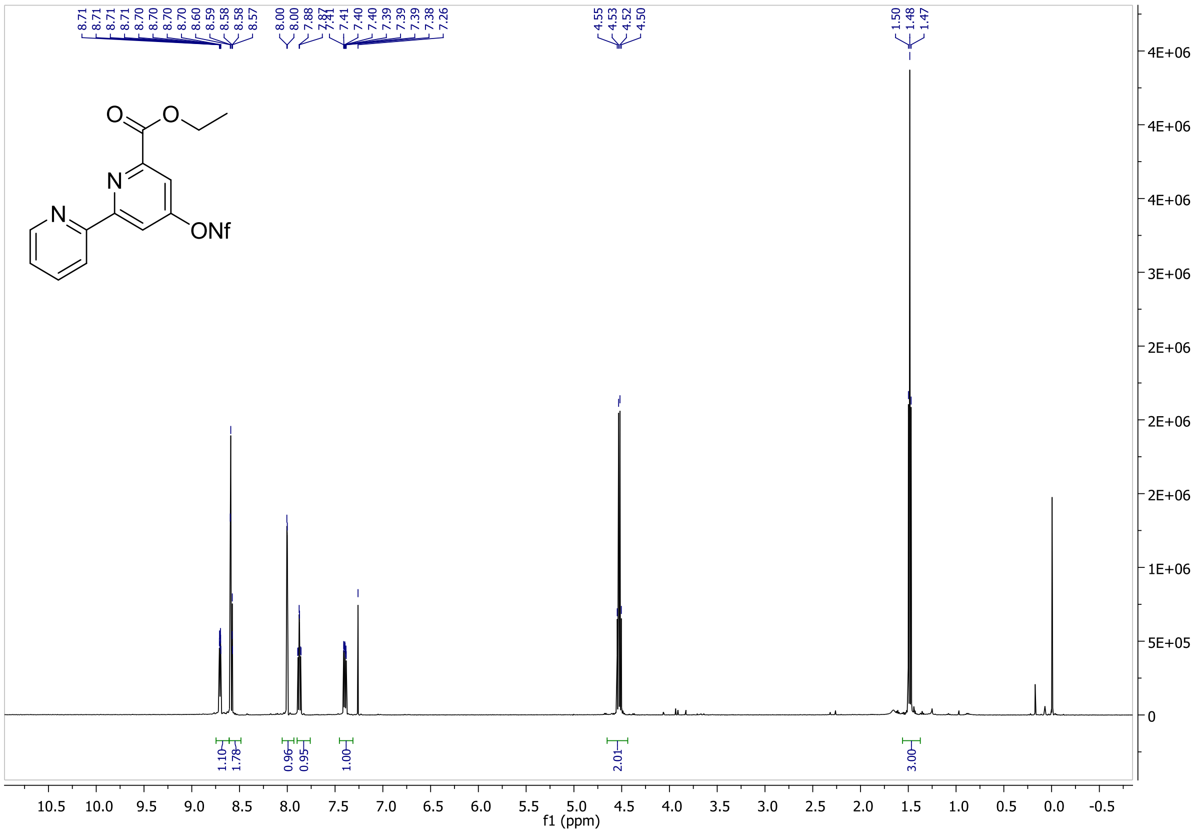

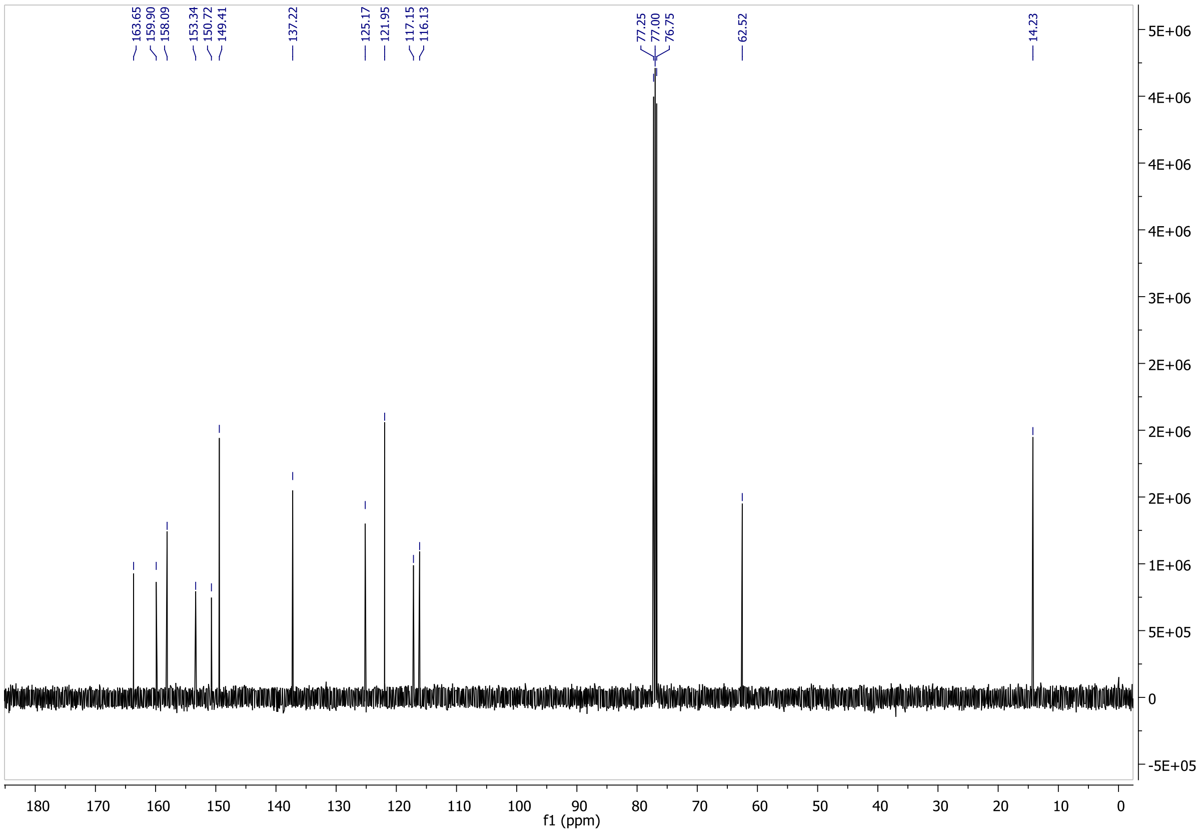

^{1}H and ^{13}C NMR of **5f**

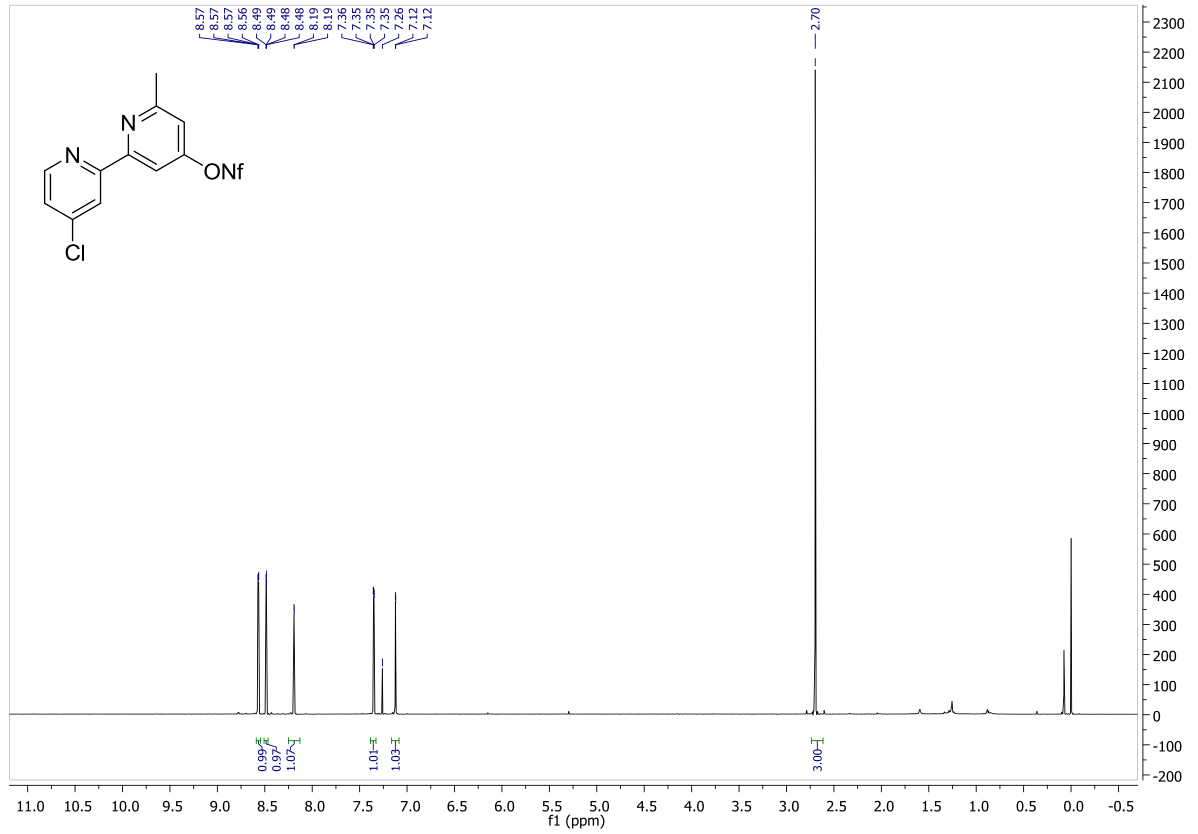

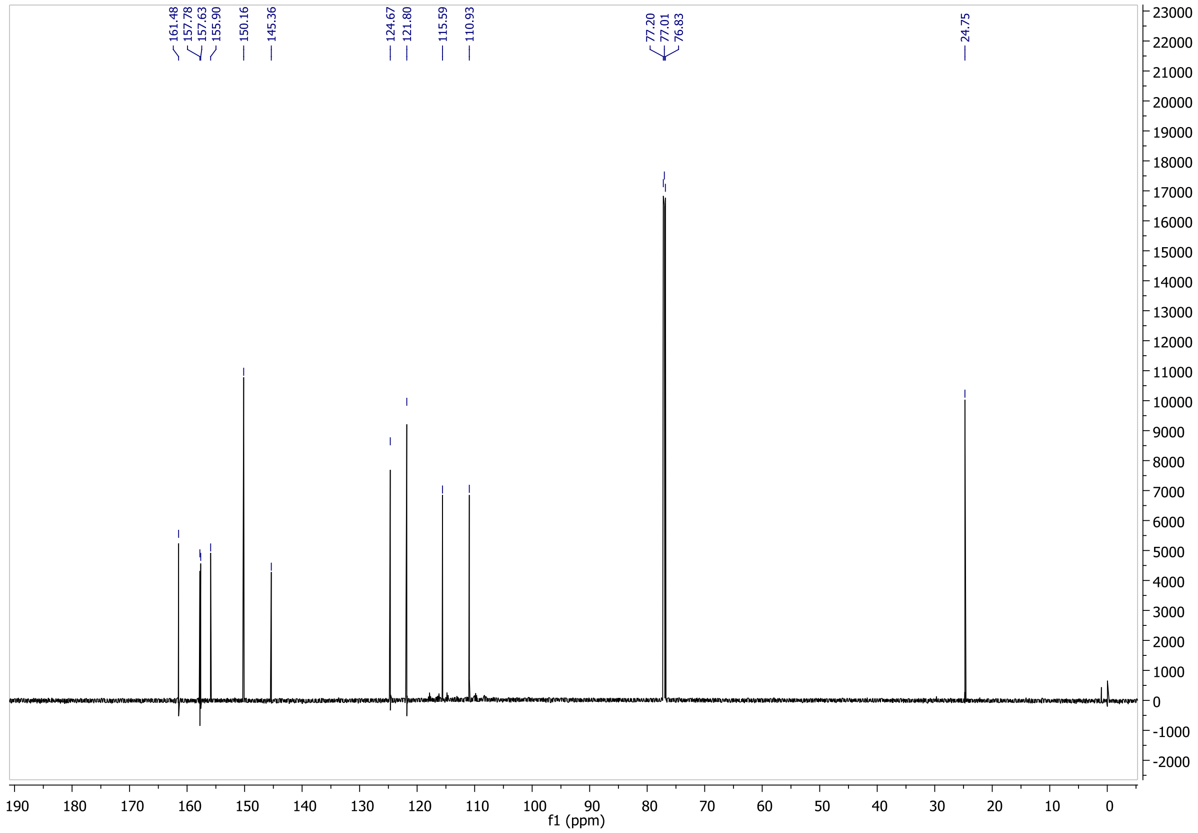

^{1}H and ^{13}C NMR of **5g**

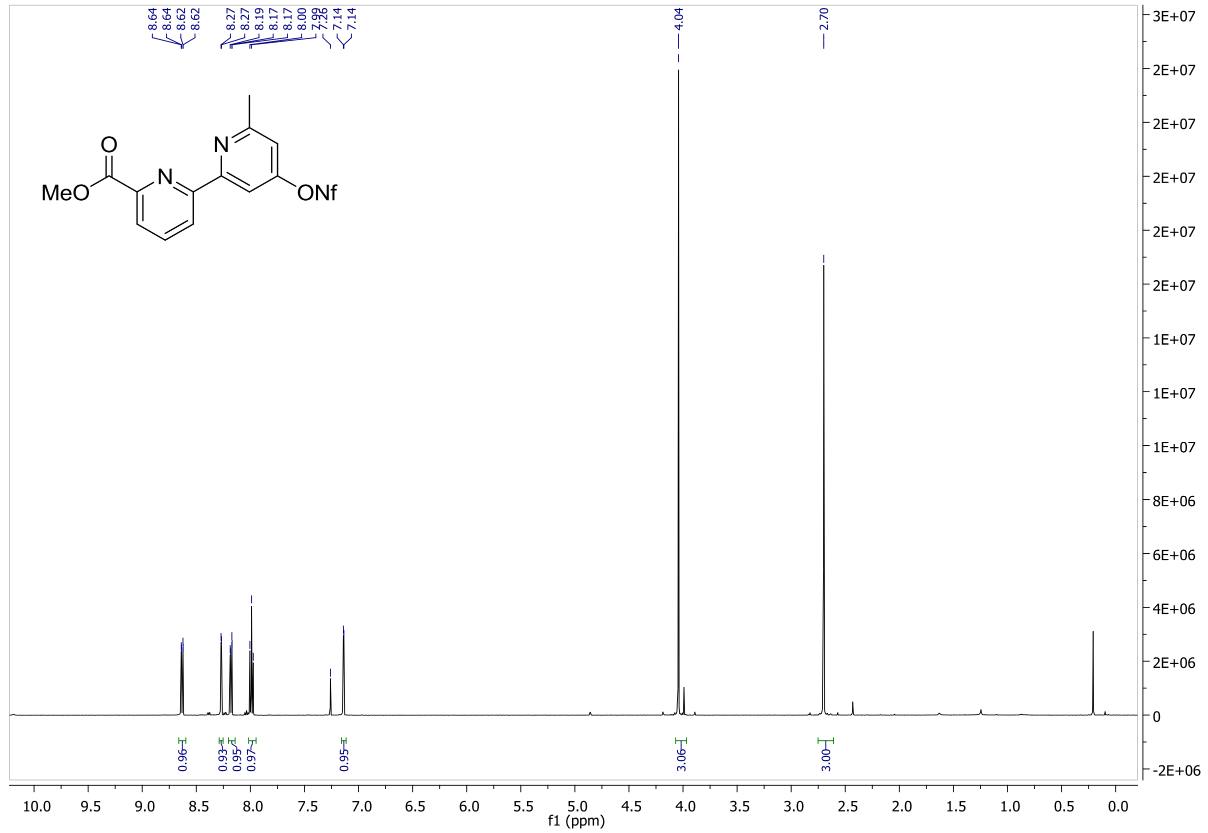

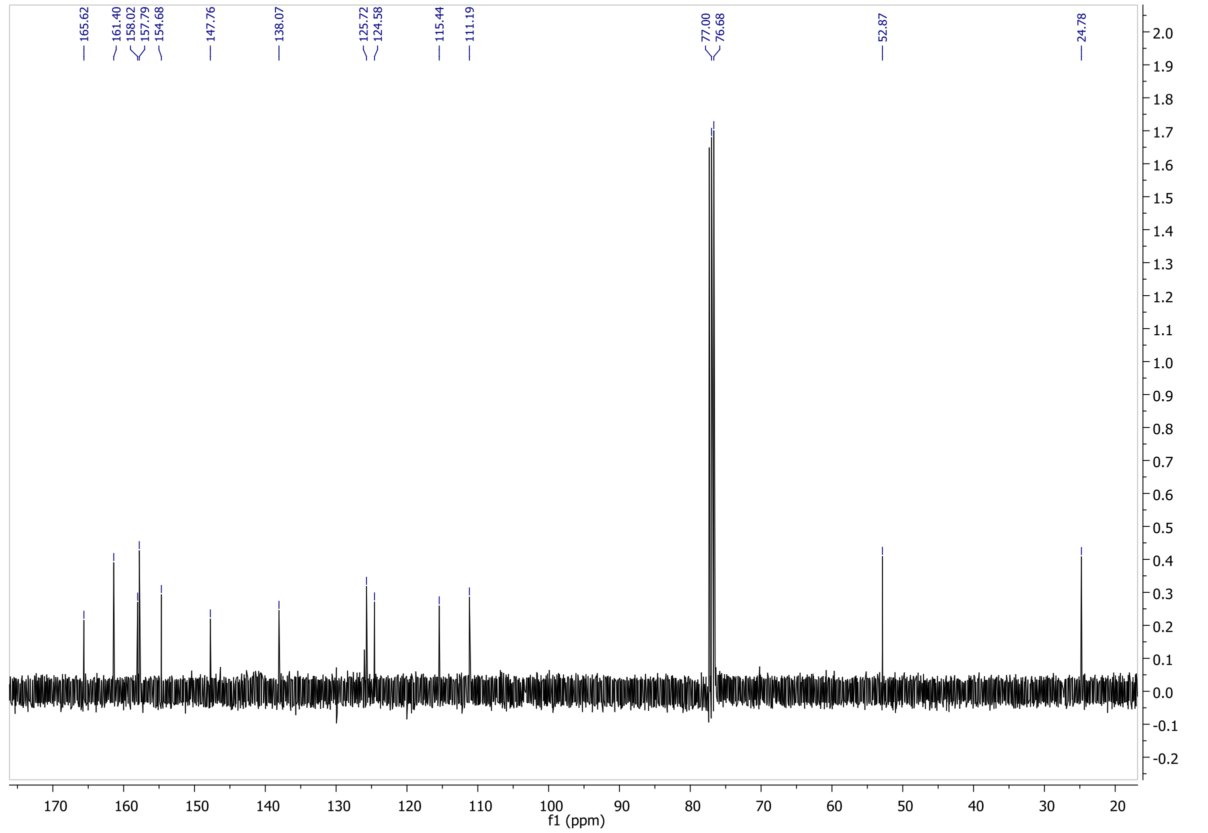

^{1}H and ^{13}C NMR of **6**

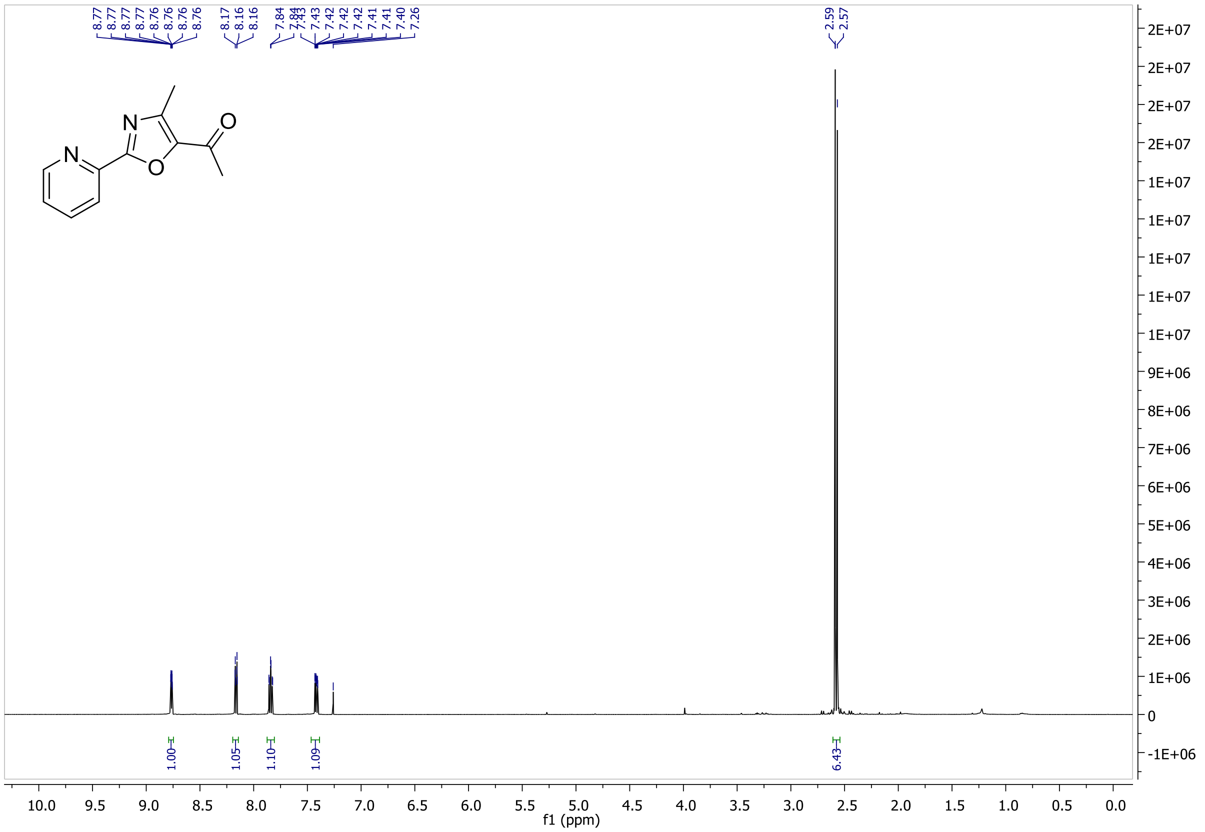

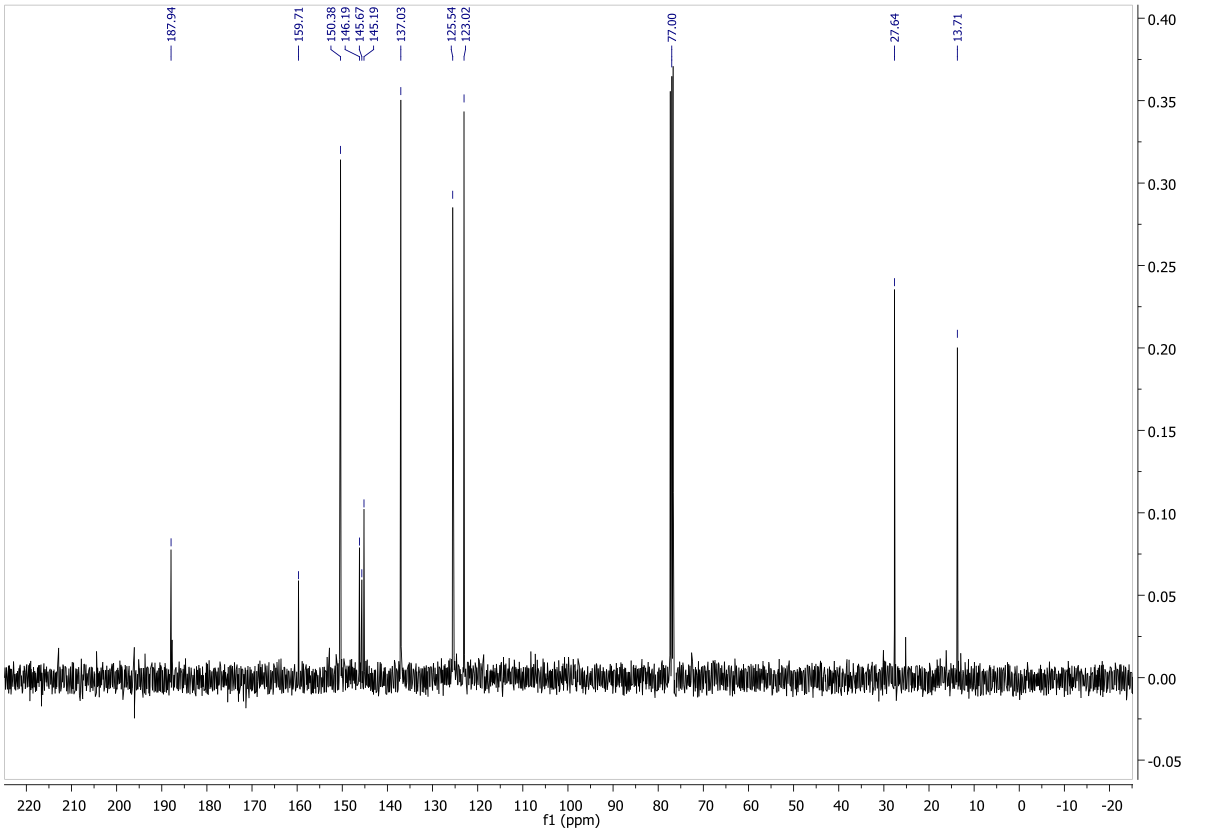

^{1}H and ^{13}C NMR of **7**

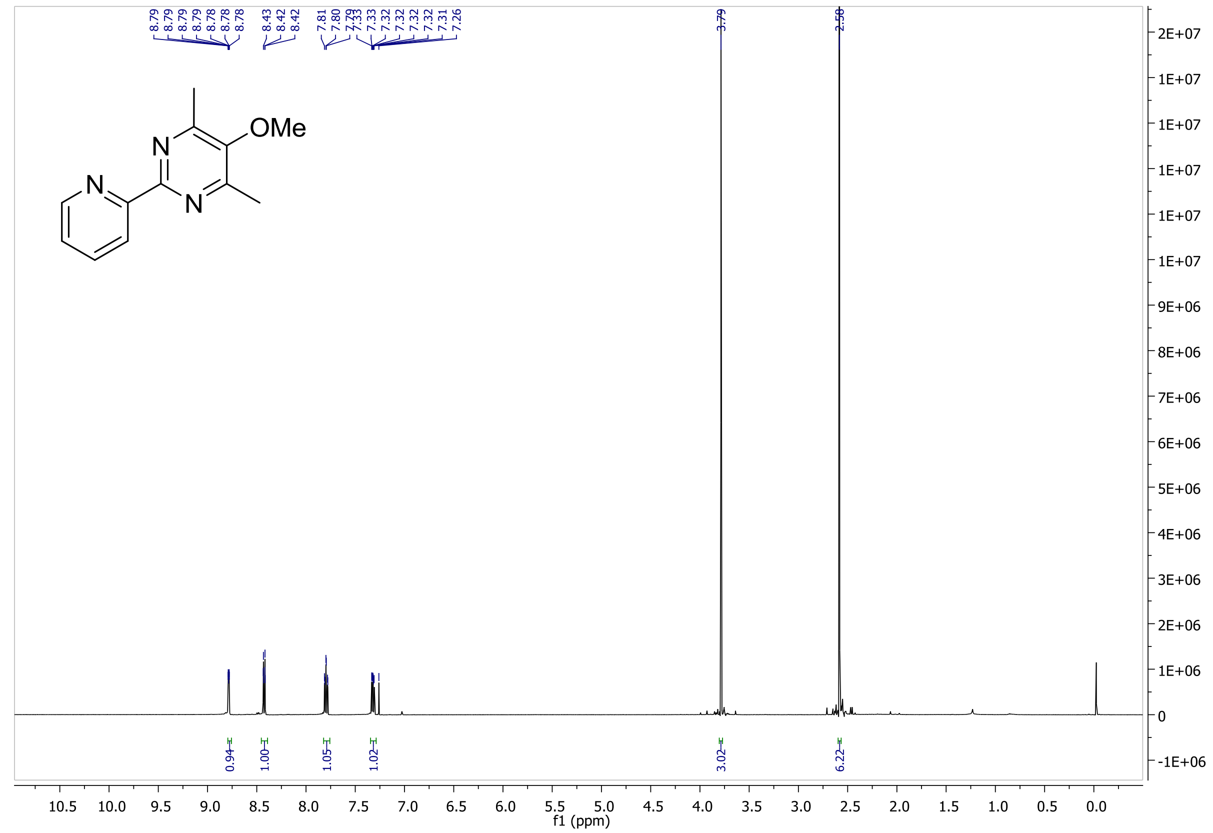

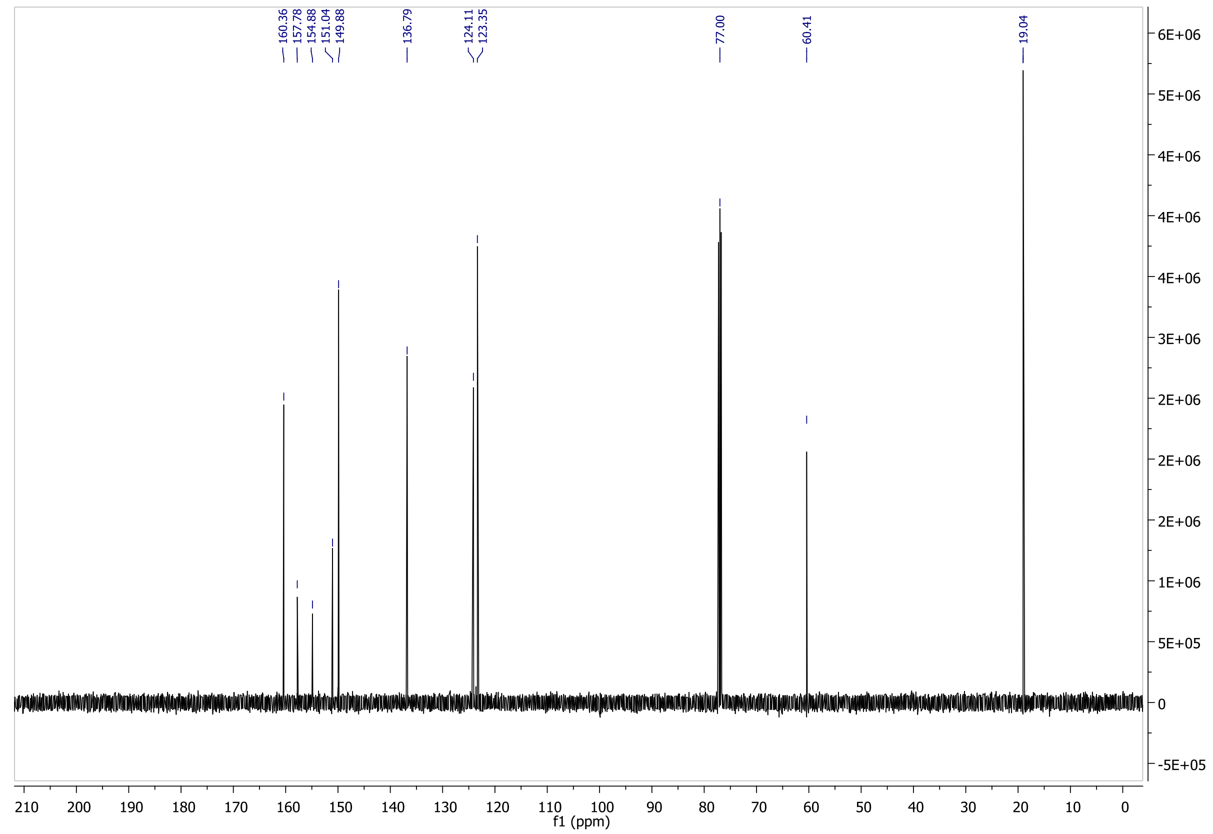

^{1}H and ^{13}C NMR of **8**

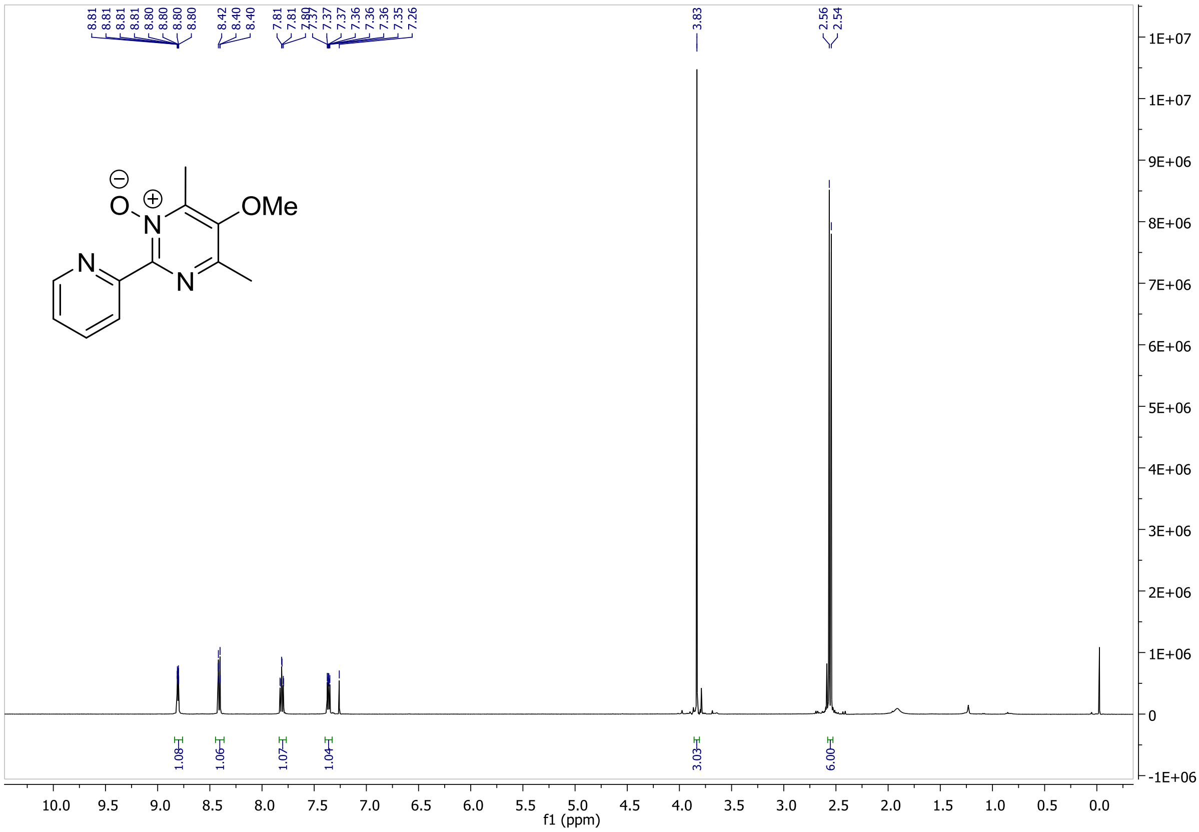

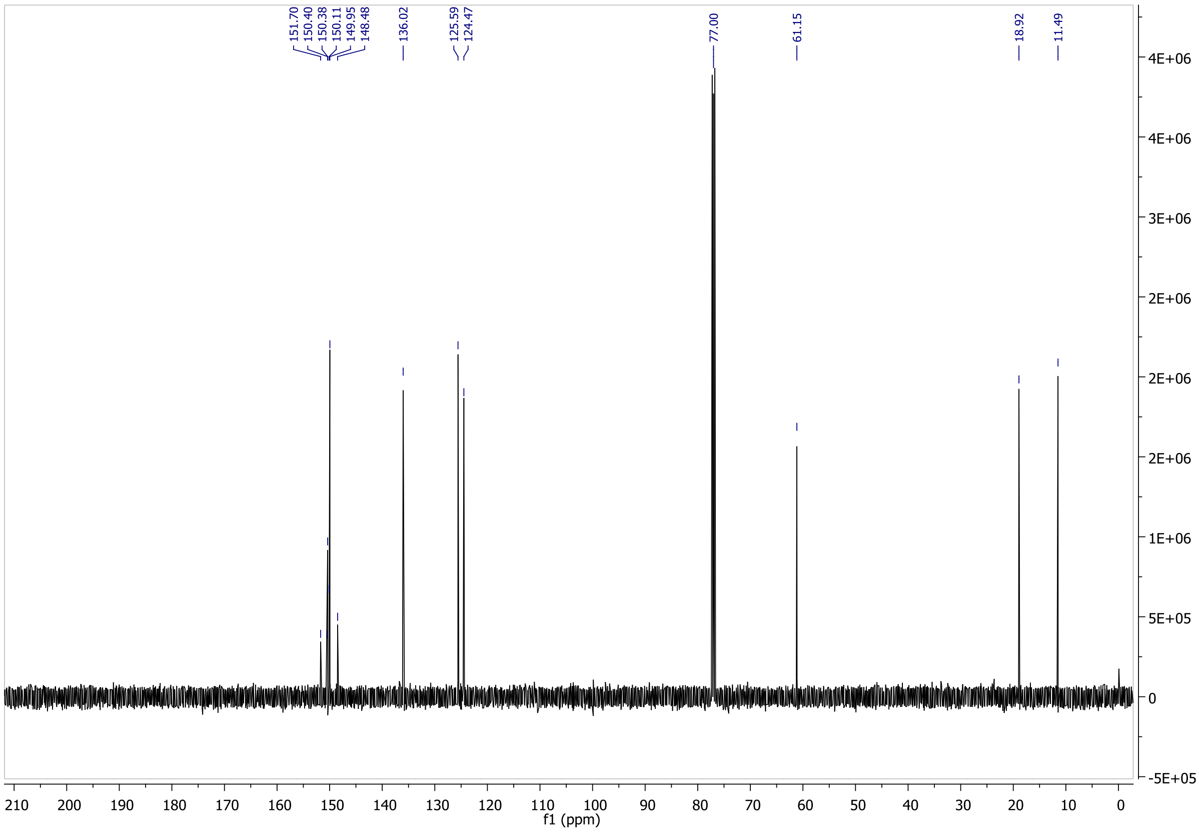

^{1}H and ^{13}C NMR of **9**

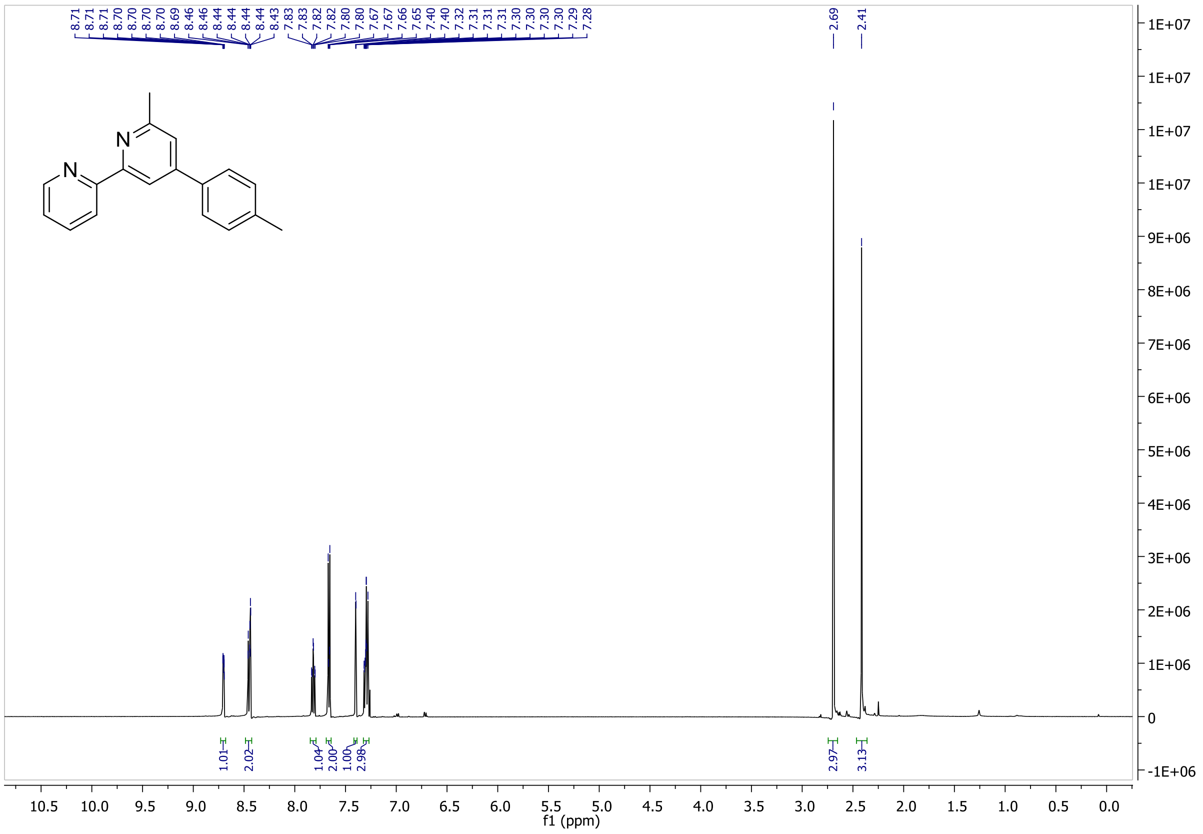

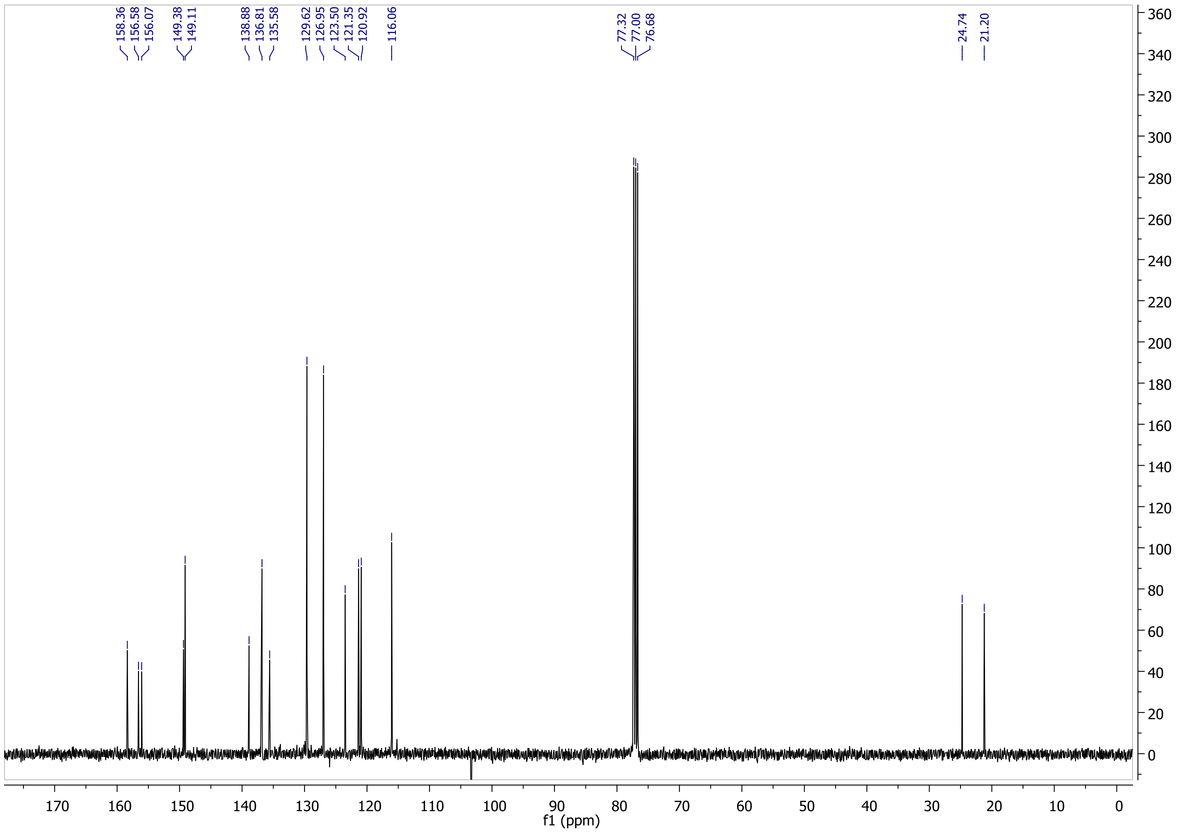

^{1}H and ^{13}C NMR of **10**

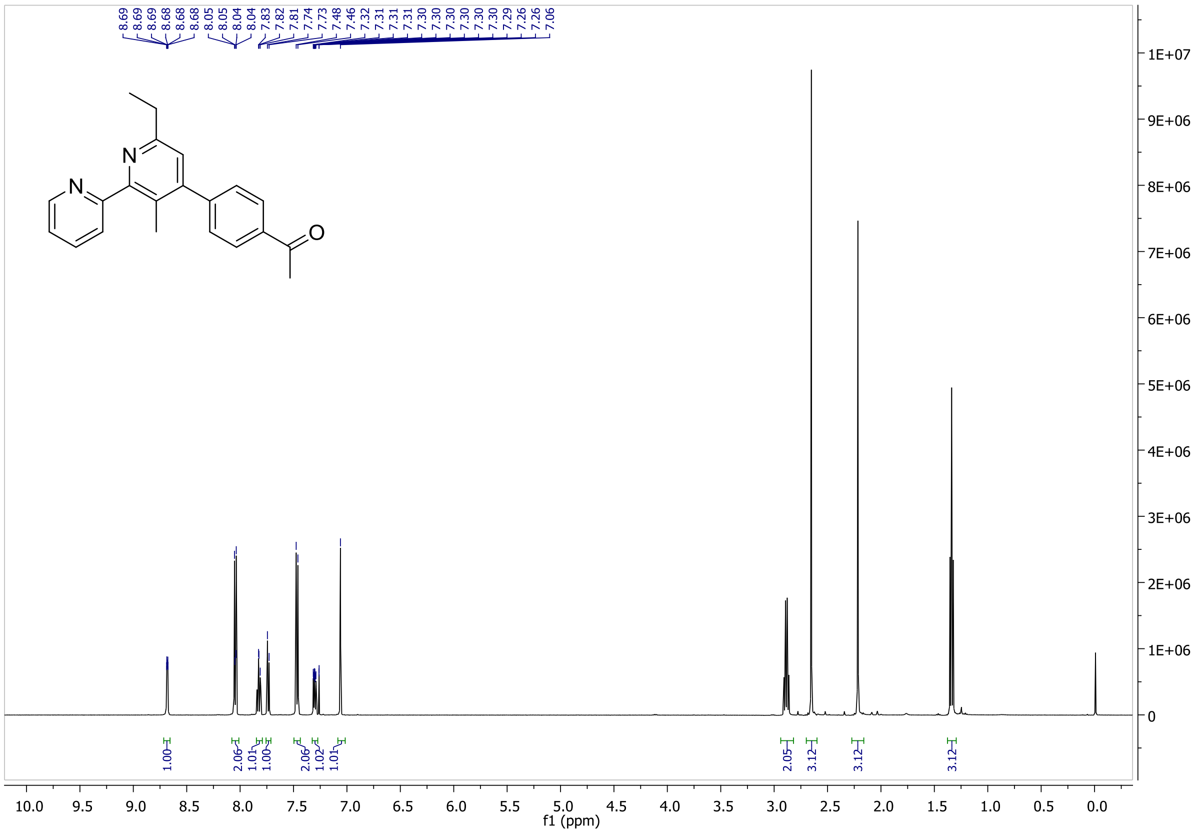

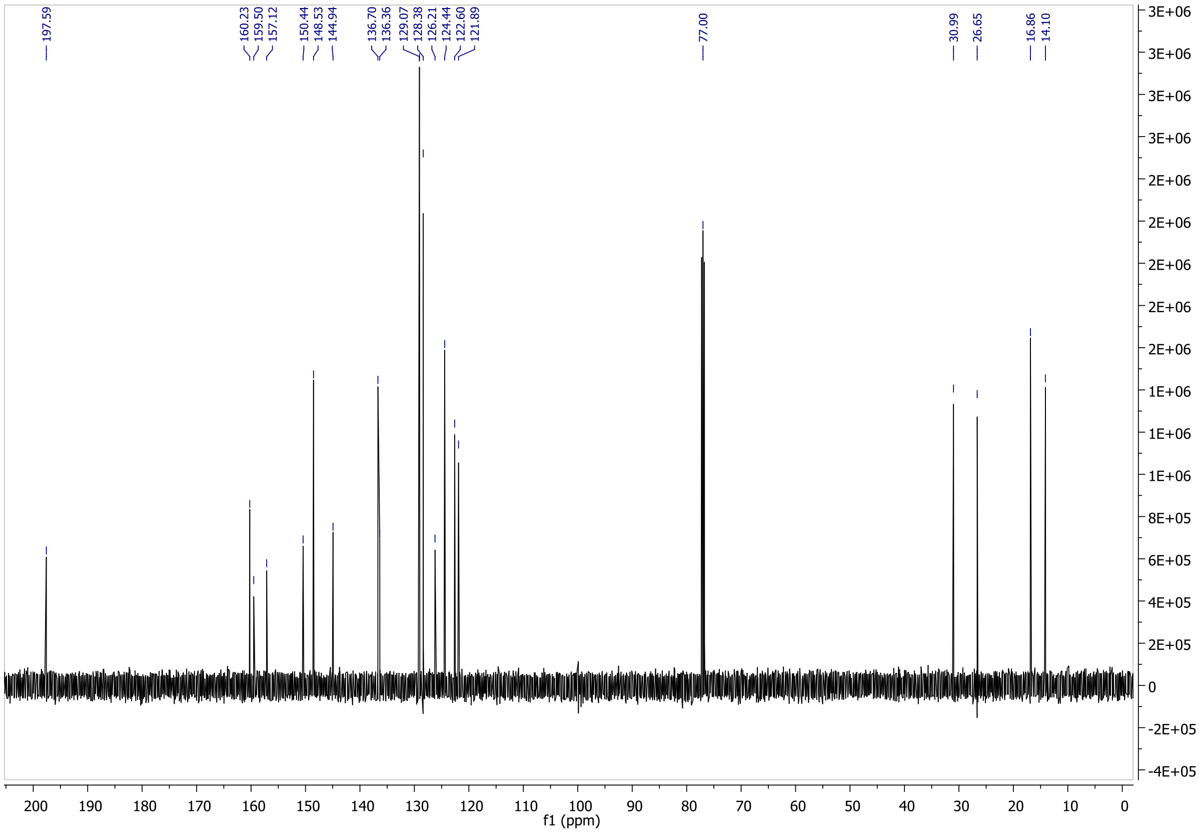

^{1}H and ^{13}C NMR of **11**

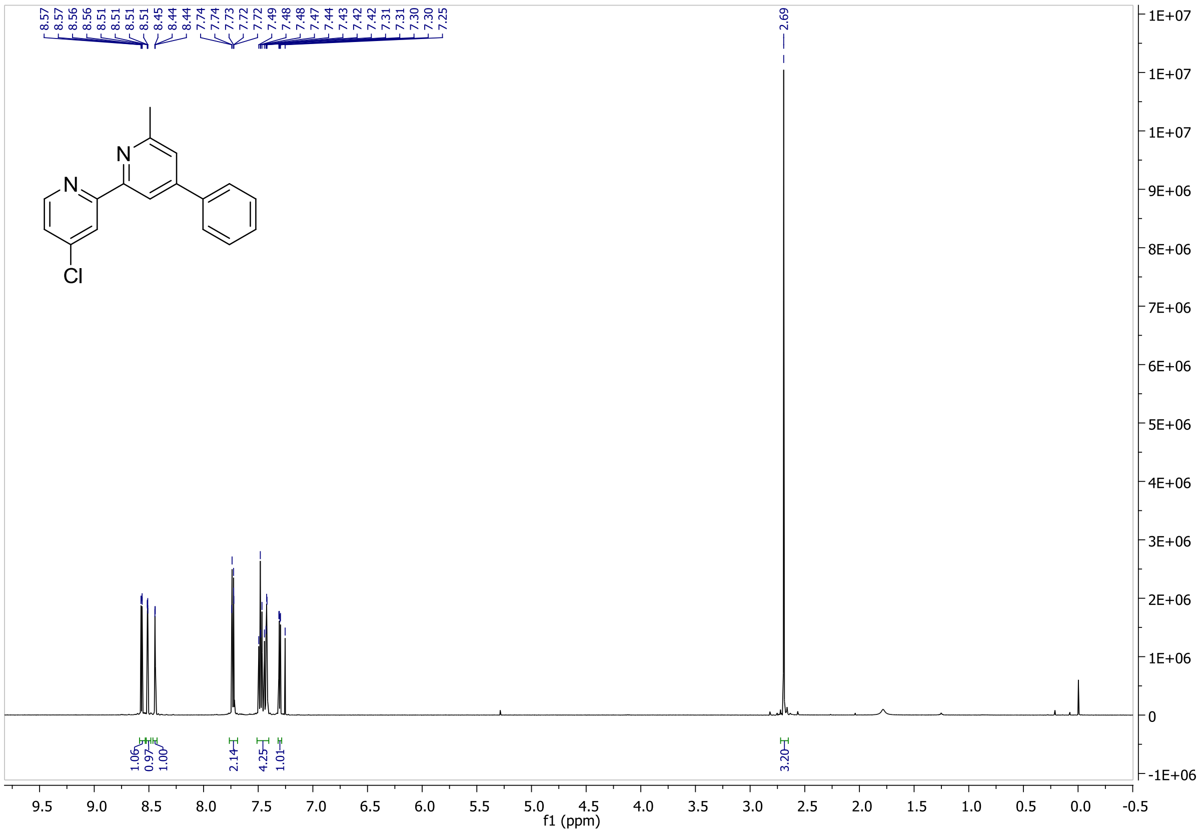

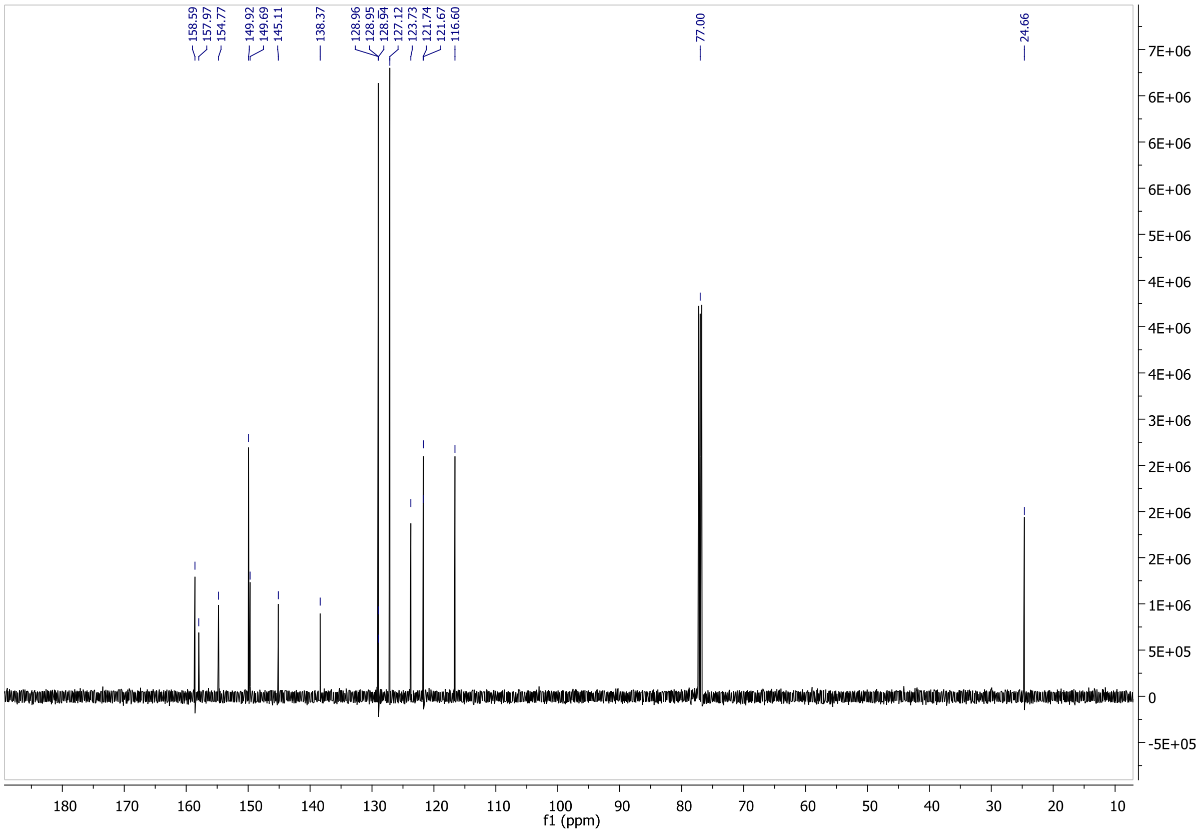

^{1}H and ^{13}C NMR of **12**

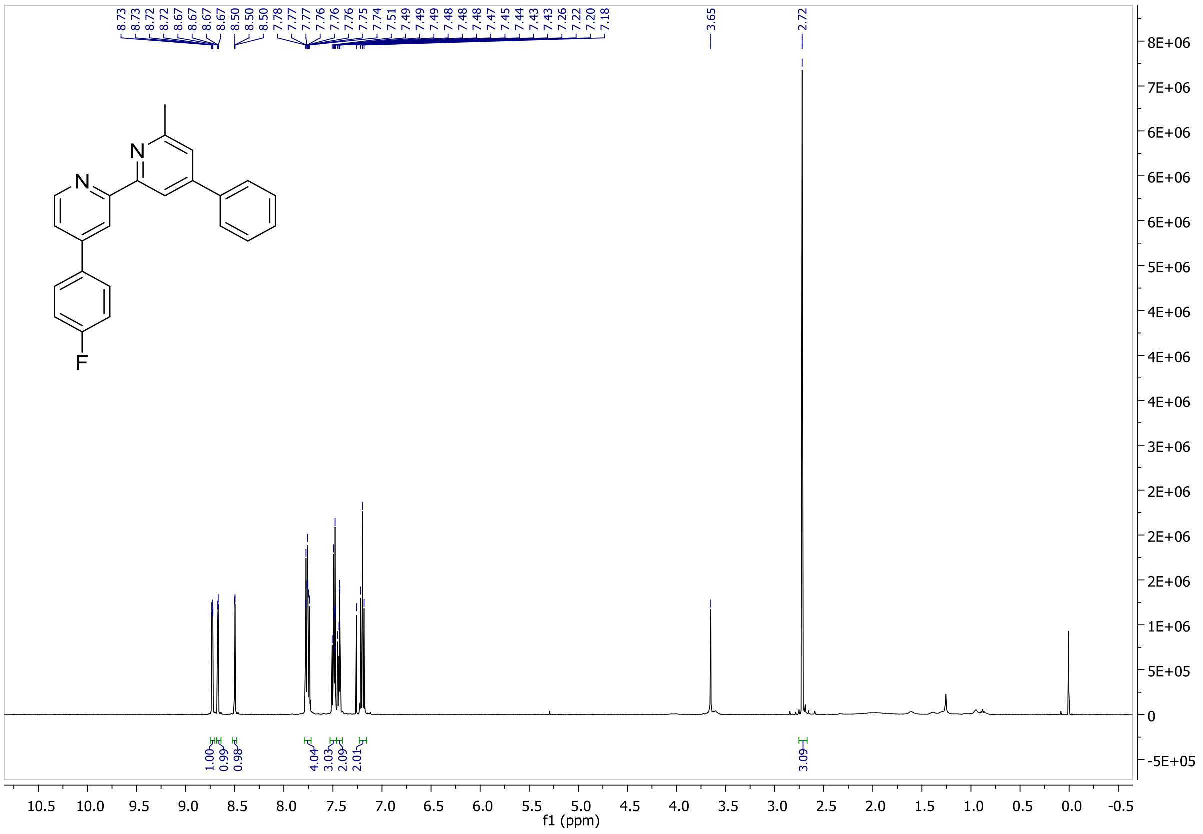

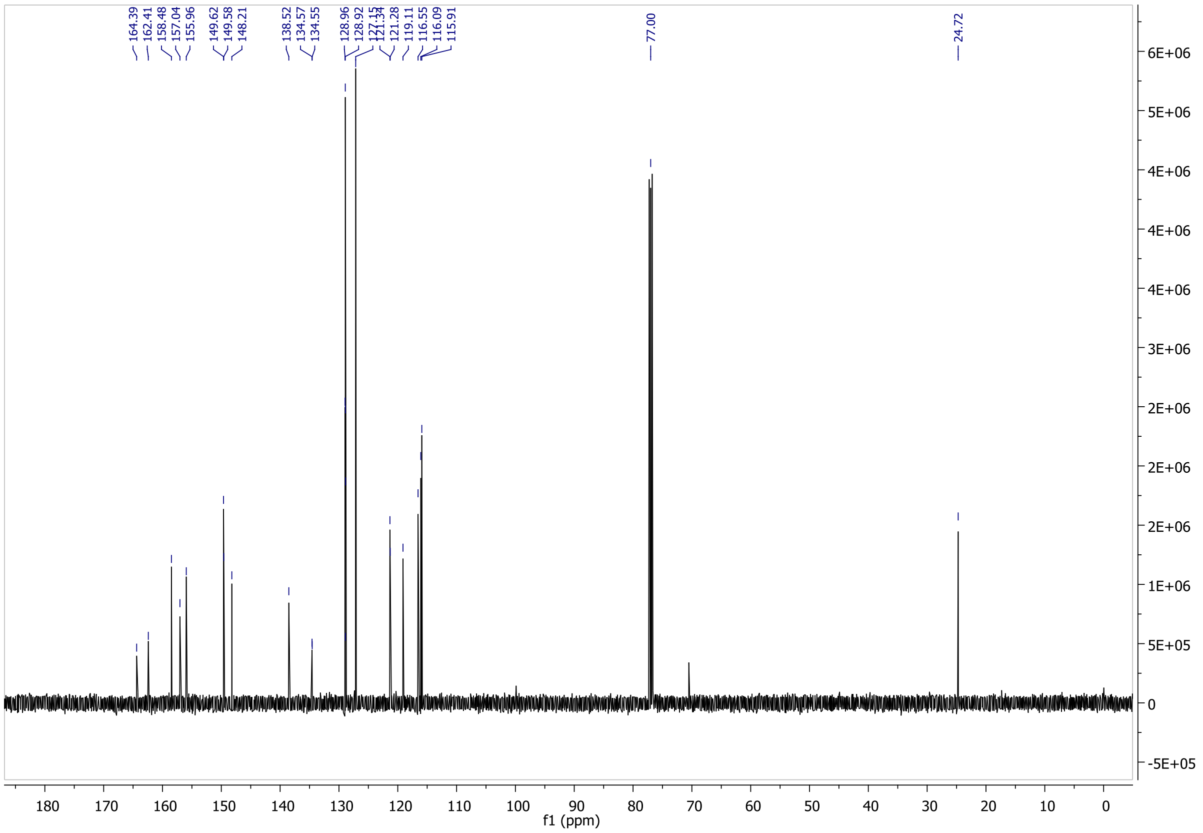

^1H and ^{13}C NMR of **13**

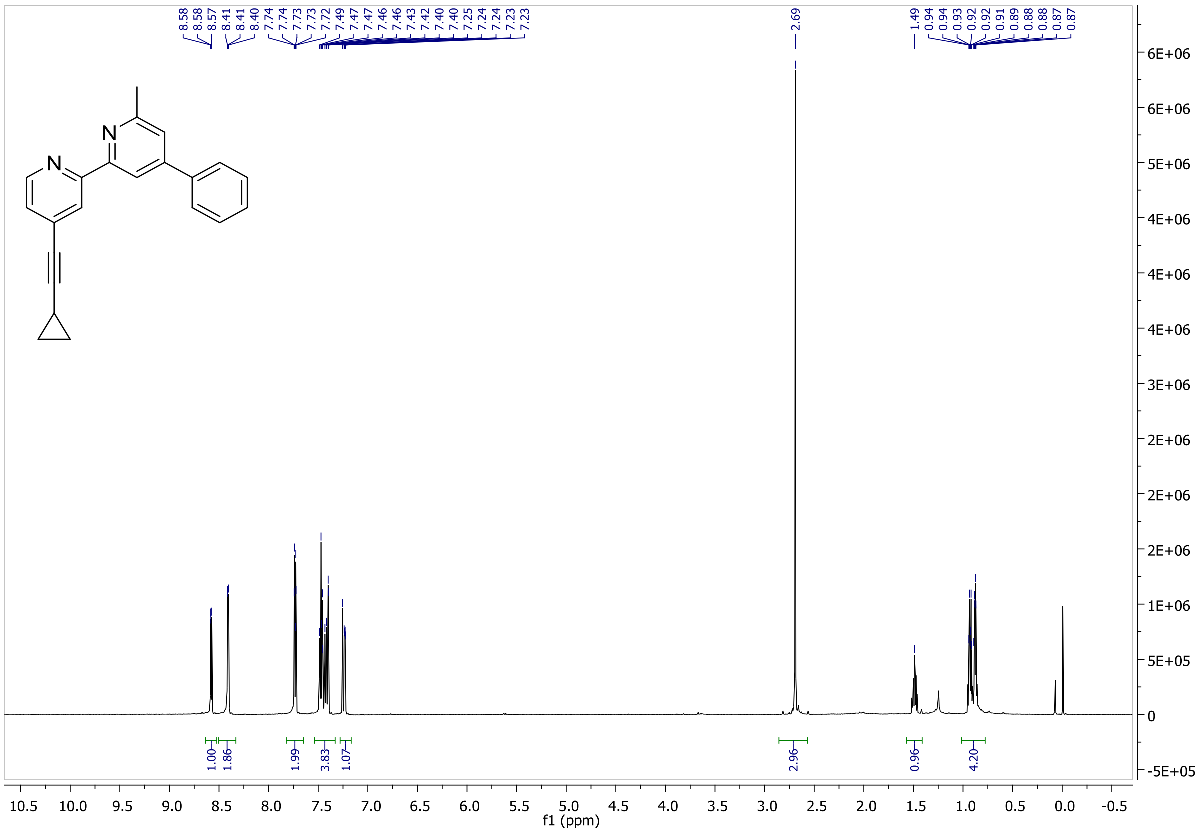

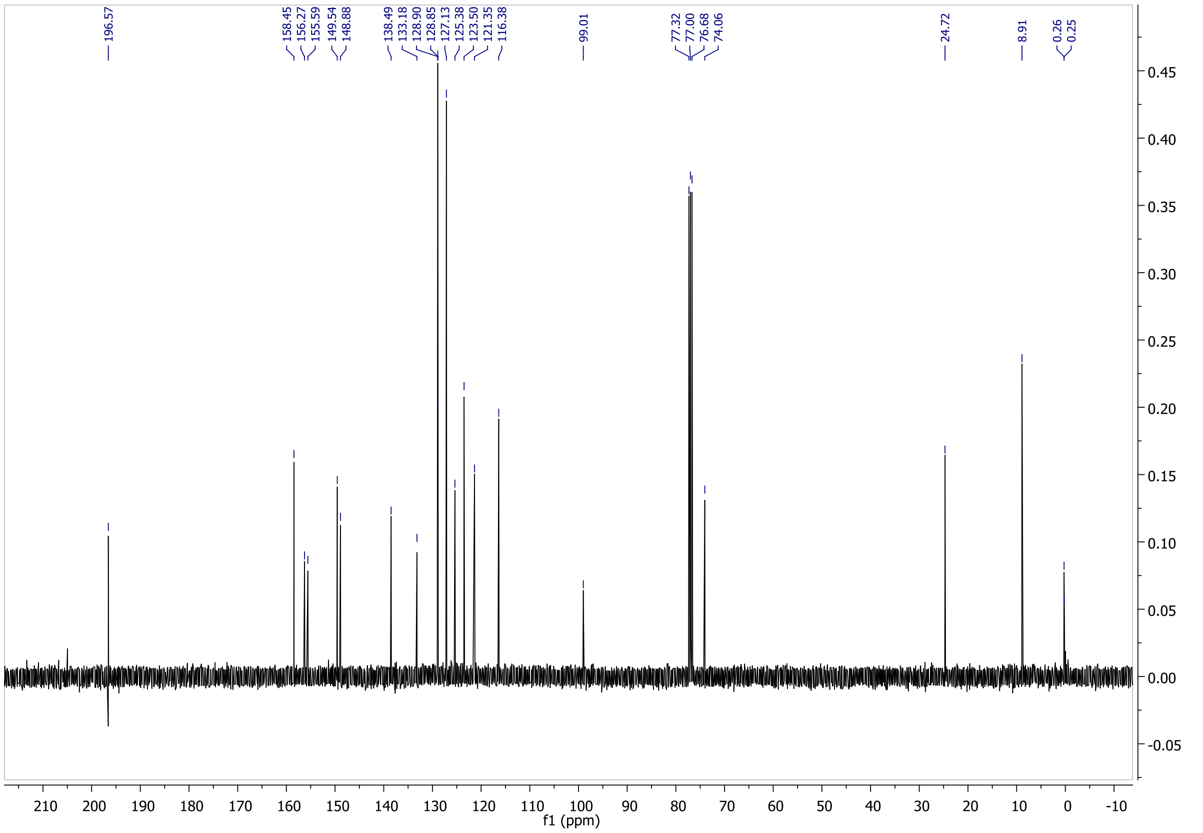

Supplement: File 1 — General information, all experimental procedures and analytical data as well as copies of NMR spectra of all compounds. [file Beilstein_J_Org_Chem-12-1170-s001.pdf]
